# Supplementary material for: Multimodal single cell analyses reveal gene networks of planarian stem cell differentiation
Source: Nat Commun. 2025 Nov 27;16:10683. doi: 10.1038/s41467-025-65712-0 (PMC12660999; doi:10.1038/s41467-025-65712-0)
Supplement: Supplementary file 1 — Supplementary Information [file 41467_2025_65712_MOESM1_ESM.pdf]

Multimodal single cell analyses reveal gene networks of planarian stem cell differentiation

Supplementary Information

Alberto Pérez-Posada [1,2,3,*], Helena García-Castro [1,2,3], Elena Emili [1,4], Anna Guixeras-Fontana [5], Virginia Vanni [1,2,3], David Salamanca-Diaz [1,2,3], Cirenia Arias-Baldrich [1], Siebren Frölich [6], Simon J. van Heeringen [6], Francesc Cebrià [5,7], Nathan Kenny [8], Jordi Solana [1,2,3,*]

[1] Department of Biological and Medical Sciences, Oxford Brookes University, Oxford, UK

[2] Living Systems Institute, University of Exeter, Exeter, UK

[3] Department of Biosciences, University of Exeter, Exeter, UK

[4] Department of Systems Medicine, University of Rome "Tor Vergata", Rome, Italy.

[5] Departament de Genètica, Microbiologia i Estadística, Facultat de Biologia, Universitat de Barcelona

[6] Department of Molecular Developmental Biology, Radboud University, Nijmegen, The Netherlands

[7] Institut de Biomedicina de la Universitat de Barcelona (IBUB)

[8] Department of Biochemistry, University of Otago, P.O. Box 56, Dunedin, Aotearoa New Zealand

[*] Corresponding authors

Supplementary Figure Legends

Supplementary Figure 1. Expression of cell markers from Emili *et al*., 2025 in our scRNA dataset.

Expression of markers from (Emili et al., 2023) in the scRNA-seq dataset from this study

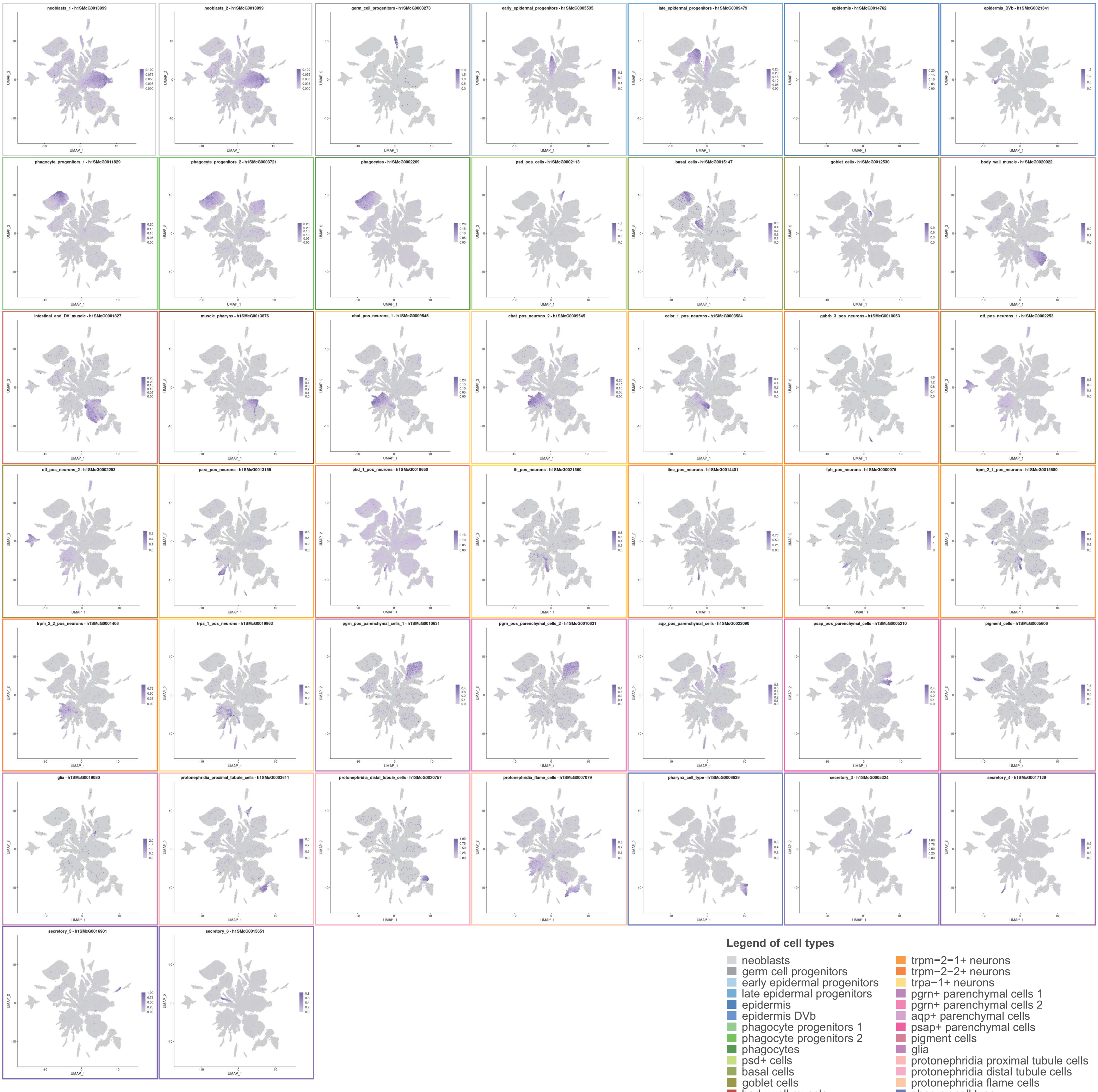

Legend of cell types

- neoblasts
- germ cell progenitors
- early epidermal progenitors
- late epidermal progenitors
- epidermis
- epidermis DVb
- phagocyte progenitors 1
- phagocyte progenitors 2
- phagocytes
- psd+ cells
- basal cells
- goblet cells
- body wall muscle
- intestinal and DV muscle
- muscle pharynx
- ChAT+ neurons
- celsr−1+ neurons
- gabrb−3+ neurons
- otf+ neurons 1
- otf+ neurons 2
- para+ neurons
- pkd−1+ neurons
- th+ neurons
- tinc+ neurons
- tph+ neurons
- trpm−2−1+ neurons
- trpm−2−2+ neurons
- trpa−1+ neurons
- pgrn+ parenchymal cells 1
- pgrn+ parenchymal cells 2
- aqp+ parenchymal cells
- psap+ parenchymal cells
- pigment cells
- glia
- protonephridia proximal tubule cells
- protonephridia distal tubule cells
- protonephridia flame cells
- pharynx cell type
- secretory 1
- secretory 2
- secretory 3
- secretory 4
- secretory 5
- secretory 6
- secretory 7

Expression of marker genes of cell types that did not cluster together in our analysis

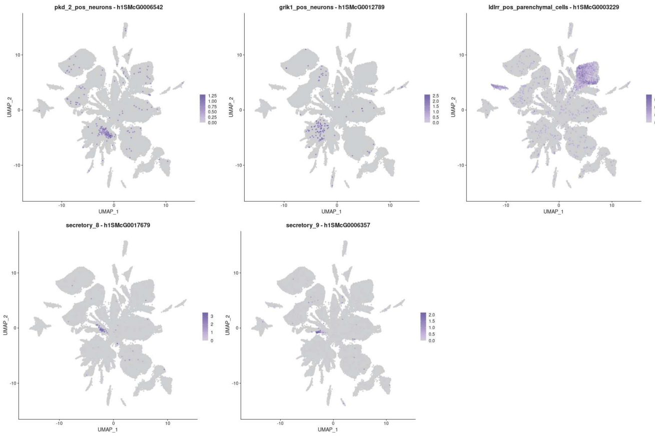

Supplementary Figure 2. A: Bipartite graph showing the transfer of labels between the reference dataset and our scRNA-seq dataset. B: Bipartite graph showing the transfer of labels from the scATAC-seq to our scRNA-seq dataset, after aligning to reference scRNA-seq dataset and transferring the cell type labels.

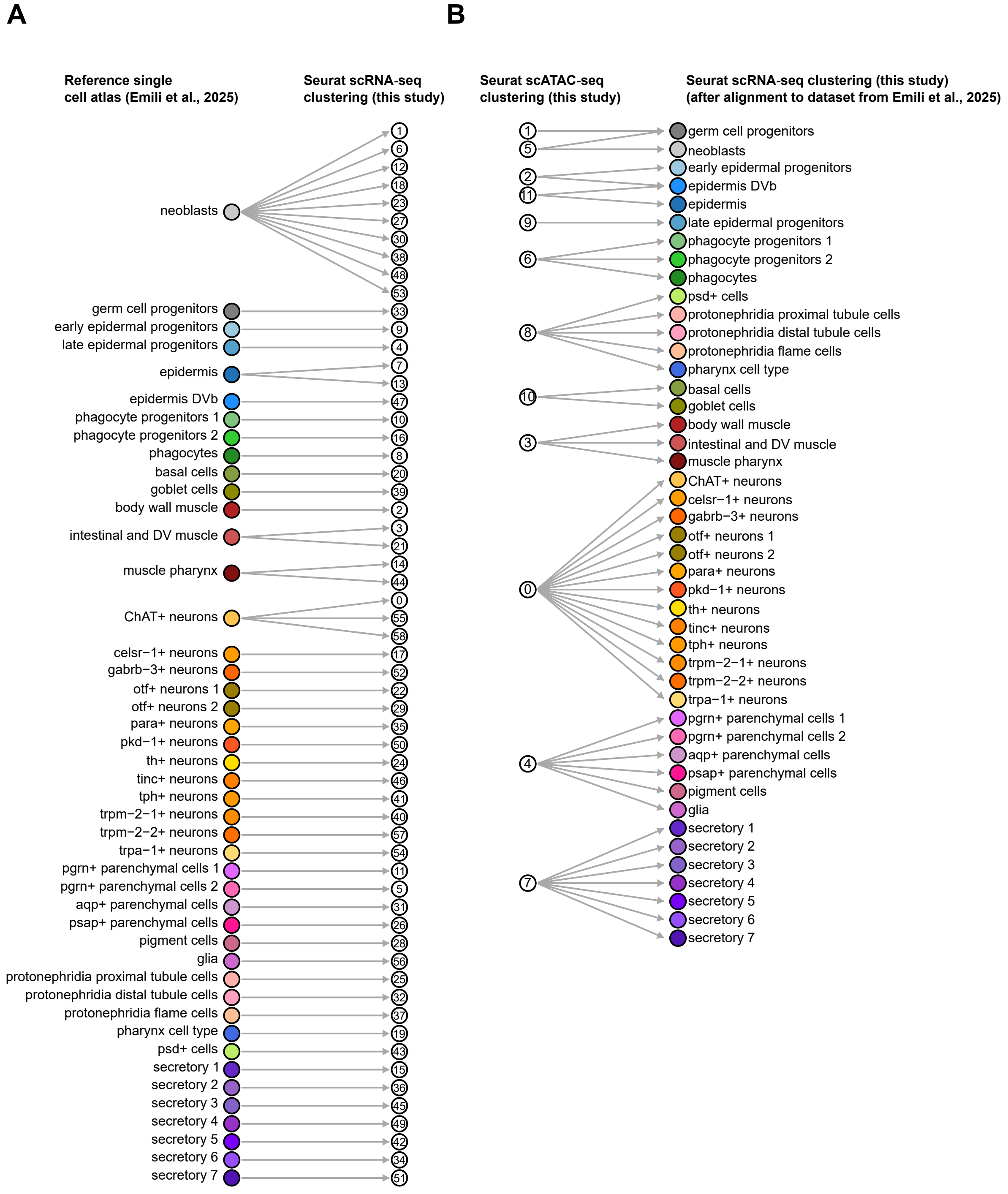

A
B
Reference single cell atlas (Emili et al., 2025)
Seurat scRNA-seq clustering (this study)
neoblasts
1
6
12
18
23
27
30
38
48
53
germ cell progenitors
33
early epidermal progenitors
9
late epidermal progenitors
4
epidermis
7
13
epidermis DVb
47
phagocyte progenitors 1
10
phagocyte progenitors 2
16
phagocytes
8
basal cells
20
goblet cells
39
body wall muscle
2
intestinal and DV muscle
3
21
muscle pharynx
14
44
ChAT+ neurons
0
55
58
celsr−1+ neurons
17
gabrb−3+ neurons
52
otf+ neurons 1
22
otf+ neurons 2
29
para+ neurons
35
pkd−1+ neurons
50
th+ neurons
24
tinc+ neurons
46
tph+ neurons
41
trpm−2−1+ neurons
40
trpm−2−2+ neurons
57
trpa−1+ neurons
54
pgrn+ parenchymal cells 1
11
pgrn+ parenchymal cells 2
5
aqp+ parenchymal cells
31
psap+ parenchymal cells
26
pigment cells
28
glia
56
protonephridia proximal tubule cells
25
protonephridia distal tubule cells
32
protonephridia flame cells
37
pharynx cell type
19
psd+ cells
43
secretory 1
15
secretory 2
36
secretory 3
45
secretory 4
49
secretory 5
42
secretory 6
34
secretory 7
51
Seurat scATAC-seq clustering (this study)
Seurat scRNA-seq clustering (this study) (after alignment to dataset from Emili et al., 2025)
1
5
germ cell progenitors
neoblasts
2
11
early epidermal progenitors
epidermis DVb
epidermis
9
late epidermal progenitors
6
phagocyte progenitors 1
phagocyte progenitors 2
phagocytes
8
psd+ cells
protonephridia proximal tubule cells
protonephridia distal tubule cells
protonephridia flame cells
pharynx cell type
10
basal cells
goblet cells
3
body wall muscle
intestinal and DV muscle
muscle pharynx
0
ChAT+ neurons
celsr−1+ neurons
gabrb−3+ neurons
otf+ neurons 1
otf+ neurons 2
para+ neurons
pkd−1+ neurons
th+ neurons
tinc+ neurons
tph+ neurons
trpm−2−1+ neurons
trpm−2−2+ neurons
trpa−1+ neurons
4
pgrn+ parenchymal cells 1
pgrn+ parenchymal cells 2
aqp+ parenchymal cells
psap+ parenchymal cells
pigment cells
glia
7
secretory 1
secretory 2
secretory 3
secretory 4
secretory 5
secretory 6
secretory 7

Supplementary Figure 3. A: Violin plots showing (top) number of genes and (bottom) number of counts per cell on each of the detected cell types in our dataset. B: From top to bottom: violin plots showing the number of OCRs and number of counts per cell, and the number of genes and Gene Activity counts per cell on the scATAC-seq dataset. C: Bar plot showing number of genes quantified on each cluster (above 5 counts). In all panels: points, data points.

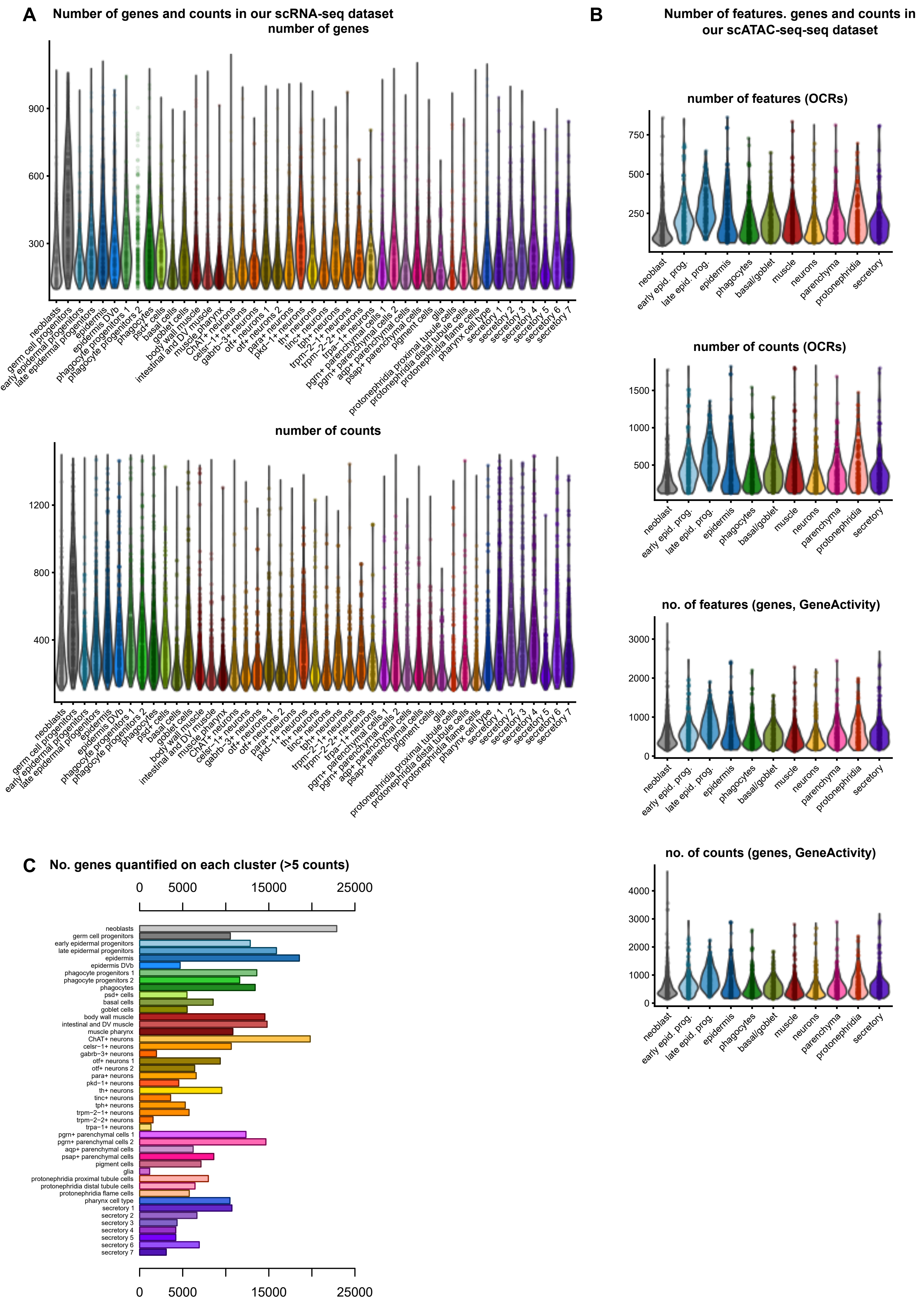
A
Number of genes and counts in our scRNA-seq dataset
number of genes
number of counts
neoblasts
germ cell progenitors
early epidermal progenitors
late epidermal progenitors
epidermis
epidermis DVb
phagocyte progenitors 1
phagocyte progenitors 2
phagocytes
psd+ cells
basal cells
goblet cells
body wall muscle
intestinal and DV muscle
muscle pharynx
ChAT+ neurons
celsr−1+ neurons
gabrb−3+ neurons
otf+ neurons 1
otf+ neurons 2
para+ neurons
pkd−1+ neurons
th+ neurons
tinc+ neurons
tph+ neurons
trpm−2−1+ neurons
trpm−2−2+ neurons
trpa−1+ neurons
pgrn+ parenchymal cells 1
pgrn+ parenchymal cells 2
aqp+ parenchymal cells
psap+ parenchymal cells
pigment cells
glia
protonephridia proximal tubule cells
protonephridia distal tubule cells
protonephridia flame cells
pharynx cell type
secretory 1
secretory 2
secretory 3
secretory 4
secretory 5
secretory 6
secretory 7
B
Number of features. genes and counts in our scATAC-seq-seq dataset
number of features (OCRs)
number of counts (OCRs)
no. of features (genes, GeneActivity)
no. of counts (genes, GeneActivity)
neoblast
early epid. prog.
late epid. prog.
epidermis
phagocytes
basal/goblet
muscle
neurons
parenchyma
protonephridia
secretory
C
No. genes quantified on each cluster (>5 counts)
0
5000
15000
25000

Supplementary Figure 4. A: Basic features of the scATAC-seq dataset. (Left) percentage of reads in OCRs, (middle) OCR region fragments, and (right) nucleosome signal per cell. B: Heatmap showing the scATAC-seq markers of all the broad cell types in the scATAC-seq data. C: Feature plots of the gene activity of the top thirty markers of neoblasts from the scRNA-seq dataset, in the scATAC-seq dataset. D-L: Boxplots showing gene activity score of the scRNA-seq markers on each of the broad cell types of the scATAC-seq dataset. Centre line, median; box limits, upper and lower quartiles; whiskers, 1.5x interquartile range; points, outliers. M-U: Chromatin accessibility profile of scRNA-seq markers on each of the broad cell types of the scATAC-seq dataset.

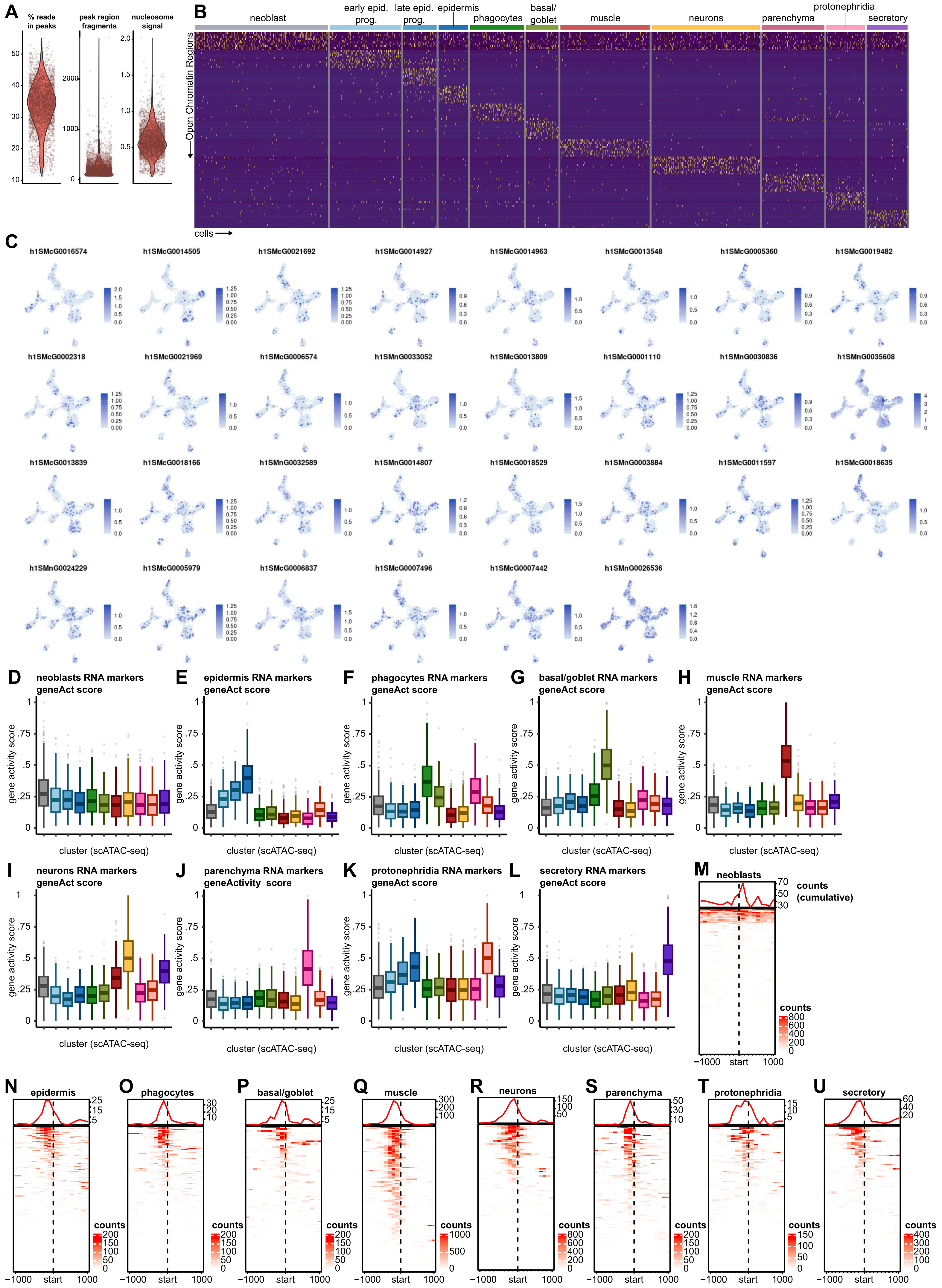

A
% reads in peaks
peak region fragments
nucleosome signal
B
neoblast
early epid. prog.
late epid. prog.
epidermis
phagocytes
basal/ goblet
muscle
neurons
parenchyma
protonephridia
secretory
Open Chromatin Regions
cells
C
h1SMcG0016574
h1SMcG0014505
h1SMcG0021692
h1SMcG0014927
h1SMcG0014963
h1SMcG0013548
h1SMcG0005360
h1SMcG0019482
h1SMcG0002318
h1SMcG0021969
h1SMcG0006574
h1SMnG0033052
h1SMcG0013809
h1SMcG0001110
h1SMnG0030836
h1SMnG0035608
h1SMcG0013839
h1SMcG0018166
h1SMnG0032589
h1SMnG0014807
h1SMcG0018529
h1SMnG0003884
h1SMcG0011597
h1SMcG0018635
h1SMnG0024229
h1SMcG0005979
h1SMcG0006837
h1SMcG0007496
h1SMcG0007442
h1SMnG0026536
D neoblasts RNA markers geneAct score
E epidermis RNA markers geneAct score
F phagocytes RNA markers geneAct score
G basal/goblet RNA markers geneAct score
H muscle RNA markers geneAct score
I neurons RNA markers geneAct score
J parenchyma RNA markers geneActivity score
K protonephridia RNA markers geneAct score
L secretory RNA markers geneAct score
gene activity score
cluster (scATAC-seq)
M neoblasts
counts (cumulative)
counts
N epidermis
O phagocytes
P basal/goblet
Q muscle
R neurons
S parenchyma
T protonephridia
U secretory
−1000
start
1000

Supplementary Figure 5. A: Overview of specific gene expression and chromatin accessibility of each broad cell type, for the same features as in Figure 1D and their respective gene annotation and open chromatin regions detected in bulk and single cell ATAC-seq. B: genomic tracks of scATAC-seq of each cell type, showing that the associated chromatin regions of the selected genes are accessible in one specific broad differentiated type and mostly inaccessible in all other broad cell type categories. C: genomic tracks of scRNA-seq of each cell type, showing that the selected genes are transcribed in one specific broad differentiated type and are expressed at much lower levels in all other broad cell type categories.

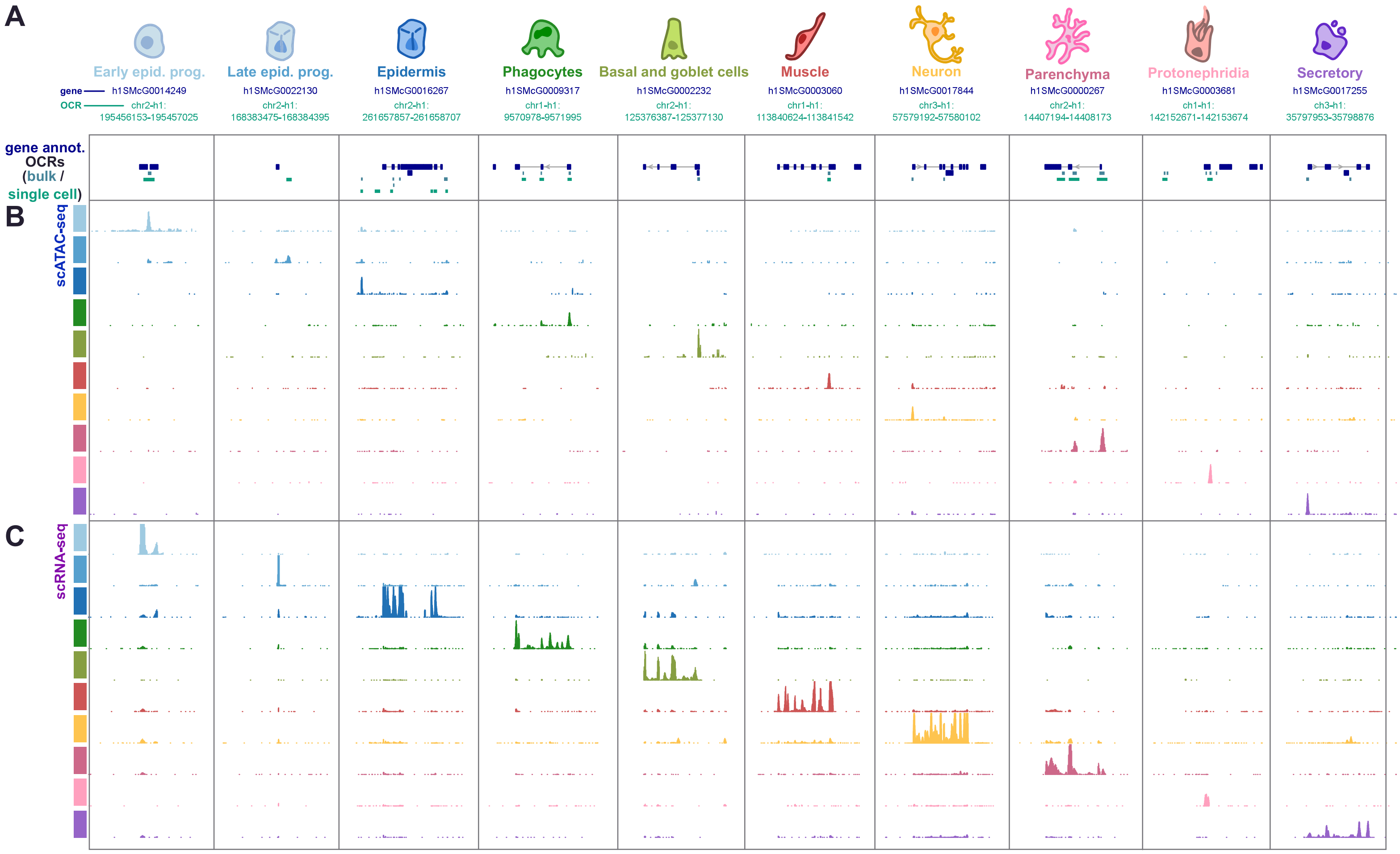
A
Early epid. prog.
Late epid. prog.
Epidermis
Phagocytes
Basal and goblet cells
Muscle
Neuron
Parenchyma
Protonephridia
Secretory
gene
OCR
h1SMcG0014249
chr2-h1:
195456153-195457025
h1SMcG0022130
chr2-h1:
168383475-168384395
h1SMcG0016267
chr2-h1:
261657857-261658707
h1SMcG0009317
chr1-h1:
9570978-9571995
h1SMcG0002232
chr2-h1:
125376387-125377130
h1SMcG0003060
chr1-h1:
113840624-113841542
h1SMcG0017844
chr3-h1:
57579192-57580102
h1SMcG0000267
chr2-h1:
14407194-14408173
h1SMcG0003681
ch1-h1:
142152671-142153674
h1SMcG0017255
ch3-h1:
35797953-35798876
gene annot.
OCRs
(bulk /
single cell)
B
scATAC-seq
C
scRNA-seq

Supplementary Figure 6. A: Chromatin profile of the OCRs detected in bulk ATAC seq using the mapping data from the scATAC-seq. B: Chromatin profile of the OCRs detected in scATAC-seq using the mapping data from the bulk ATAC-seq.

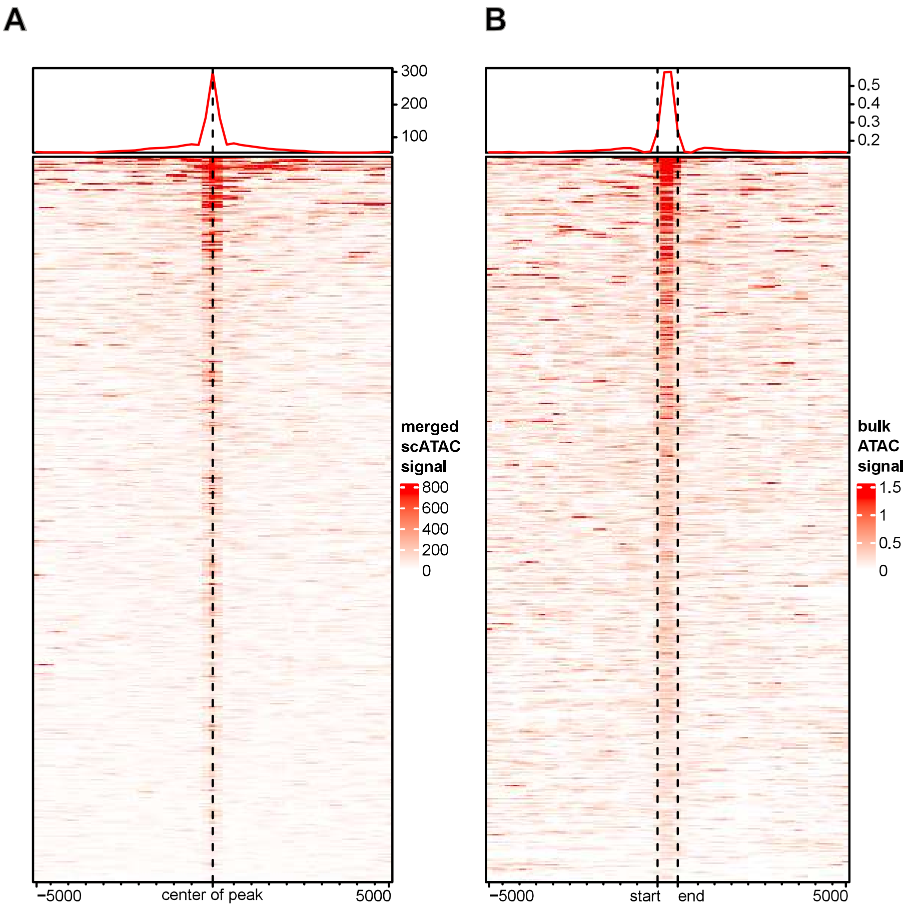

A
B
300
200
100
merged
scAATAC
signal
800
600
400
200
0
−5000
center of peak
5000
0.5
0.4
0.3
0.2
bulk
ATAC
signal
1.5
1
0.5
0
−5000
start
end
5000

Supplementary Figure 7. A: Schematic of the pseudobulk computational dissection without replicates or conditions. B: Scale-free topology model fit of different networks obtained from raising the gene-wise correlations of the pseudobulk data raised to fifteen different soft power thresholds. C: Mean connectivity of different networks obtained from raising the gene-wise correlations of the pseudobulk data to fifteen different soft power thresholds. D: (Top) gene tree of topology overlap and (bottom) module association. E: Schematics of the rationale for sorting modules based on upper quartile values of expression across cell types. F: Distribution of relativised upper quartiles of normalised expression on each cell type per module. G: (left) scatter plot showing the relationship between the coefficient of variation and the Tau specificity metric; (right) box plot showing the distribution of tau coefficients for genes that did not pass the filter to enter WGCNA (left, grey), genes classified as belonging to 'm' modules of co-expression (centre, blue), and genes classified as belonging to 's' modules of co-expression (right, red).

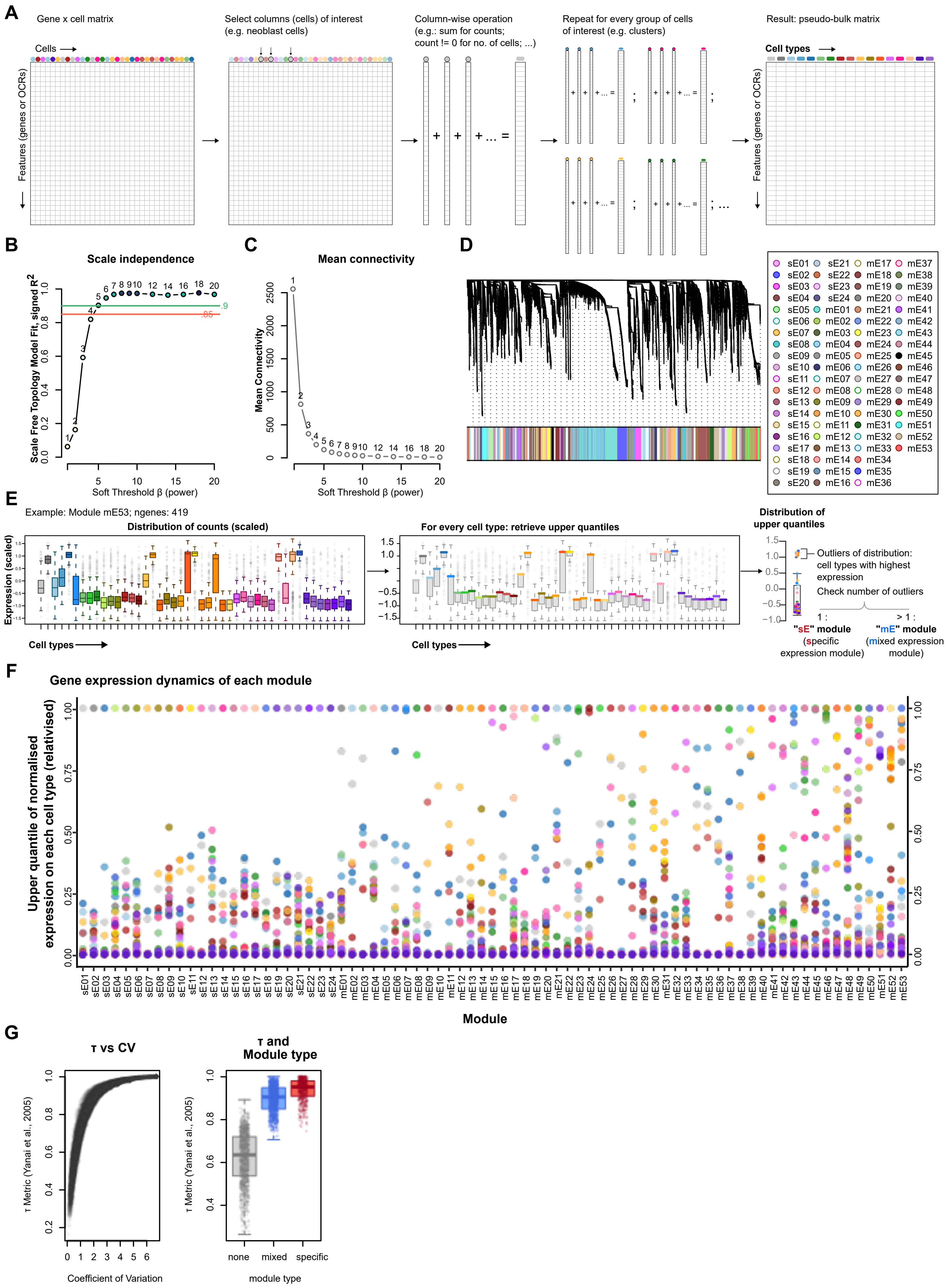
A
Gene x cell matrix
Cells
Features (genes or OCRs)
Select columns (cells) of interest
(e.g. neoblast cells)
Column-wise operation
(e.g.: sum for counts;
count != 0 for no. of cells; ...)
Repeat for every group of cells
of interest (e.g. clusters)
Result: pseudo-bulk matrix
Cell types
B
Scale independence
Scale Free Topology Model Fit, signed R2
Soft Threshold β (power)
.9
.85
C
Mean connectivity
Mean Connectivity
Soft Threshold β (power)
D
sE01 sE02 sE03 sE04 sE05 sE06 sE07 sE08 sE09 sE10 sE11 sE12 sE13 sE14 sE15 sE16 sE17 sE18 sE19 sE20
sE21 sE22 sE23 sE24 mE01 mE02 mE03 mE04 mE05 mE06 mE07 mE08 mE09 mE10 mE11 mE12 mE13 mE14 mE15 mE16
mE17 mE18 mE19 mE20 mE21 mE22 mE23 mE24 mE25 mE26 mE27 mE28 mE29 mE30 mE31 mE32 mE33 mE34 mE35 mE36
mE37 mE38 mE39 mE40 mE41 mE42 mE43 mE44 mE45 mE46 mE47 mE48 mE49 mE50 mE51 mE52 mE53
E
Example: Module mE53; ngenes: 419
Distribution of counts (scaled)
Expression (scaled)
Cell types
For every cell type: retrieve upper quantiles
Cell types
Distribution of upper quantiles
Outliers of distribution: cell types with highest expression
Check number of outliers
1 :
"sE" module
(specific expression module)
> 1 :
"mE" module
(mixed expression module)
F
Gene expression dynamics of each module
Upper quantile of normalised expression on each cell type (relativised)
Module
G
τ vs CV
τ Metric (Yanai et al., 2005)
Coefficient of Variation
τ and Module type
τ Metric (Yanai et al., 2005)
none
mixed
specific
module type

Supplementary Figure 8. A: Heatmap of gene expression of genes from different WGCNA co-expression modules across cell types in *S. mediterranea*. Genes in rows, cell types in columns. A random subset of thirty genes per co-expression module is shown. Module identity labels on the left side. On the right side, stacked bar plot of average gene expression of each co-expression module in each cell type (see Methods; Supplementary Note 1). On the right-most side, bar plot showing log number of genes per co-expression module, and values of Tau specificity metric of the average expression profile of the module. B: (left) Heatmap of Transcription Factor (TF) connectivity to each co-expression module in *S. mediterranea*. TFs in rows, modules in columns. TFs have been sorted by highest connectivity value. On the top, stacked bar plot of average gene expression as explained in A. (right) Heatmap of gene expression fold change of the same TFs on each cell type. TFs in rows, cell types in columns. TFs follow the same order as in the heatmap to the left. Several TFs known in the literature are highlighted. C: Heatmap showing the correlation between TF connectivity with co-expression modules, and motif enrichment in gene promoters of co-expression modules, of several TFs and DNA motifs. D: from left to right, top to bottom: scatter plots showing the connectivity of the TFs *egr-1*, *soxP-1*, *foxF-1*, *mitfl-1*, *coe*, *dll-1*, *ascl-2*, *atoh8-2*, and *foxa1*, to modules of co-accessibility, in relation to the motif enrichment of the same TF class in gene promoters of co-expression modules. Dot size indicates significance of hypergeometric test (q-value < 0.1).

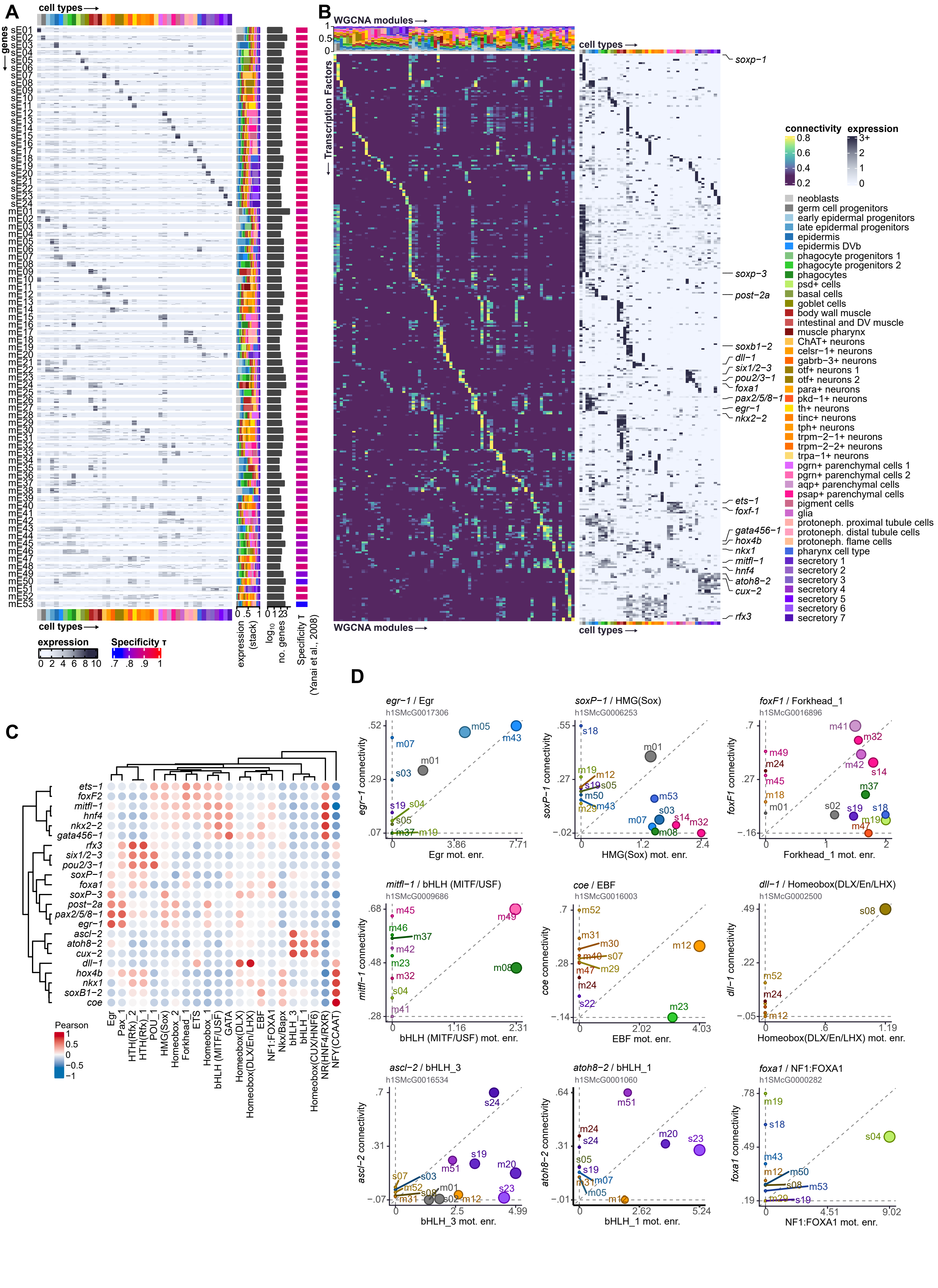

A
cell types
genes
expression
Specificity τ
0 2 4 6 8 10
.7 .8 .9 1
expression (stack)
log10 no. genes
Specificity τ (Yanai et al., 2008)
B
WGCNA modules
Transcription Factors
cell types
connectivity
expression
soxp−1
soxp−3
post−2a
soxb1−2
dll−1
six1/2−3
pou2/3−1
foxa1
pax2/5/8−1
egr−1
nkx2−2
ets−1
foxf−1
gata456−1
hox4b
nkx1
mitfl−1
hnf4
atoh8−2
cux−2
rfx3
neoblasts
germ cell progenitors
early epidermal progenitors
late epidermal progenitors
epidermis
epidermis DVb
phagocyte progenitors 1
phagocyte progenitors 2
phagocytes
psd+ cells
basal cells
goblet cells
body wall muscle
intestinal and DV muscle
muscle pharynx
ChAT+ neurons
celsr−1+ neurons
gabrb−3+ neurons
otf+ neurons 1
otf+ neurons 2
para+ neurons
pkd−1+ neurons
th+ neurons
tinc+ neurons
tph+ neurons
trpm−2−1+ neurons
trpm−2−2+ neurons
trpa−1+ neurons
pgrn+ parenchymal cells 1
pgrn+ parenchymal cells 2
aqp+ parenchymal cells
psap+ parenchymal cells
pigment cells
glia
protoneph. proximal tubule cells
protoneph. distal tubule cells
protoneph. flame cells
pharynx cell type
secretory 1
secretory 2
secretory 3
secretory 4
secretory 5
secretory 6
secretory 7
C
Pearson
Egr
Pax_1
HTH(Rfx)_2
HTH(Rfx)_1
POU_1
HMG(Sox)
Homeobox_2
Forkhead_1
ETS
Homeobox_1
bHLH (MITF/USF)
GATA
Homeobox(DLX)
Homeobox(DLX/En/LHX)
EBF
NF1:FOXA1
Nkx/Bapx
bHLH_3
bHLH_1
Homeobox(CUX/HNF6)
NR(HNF4/RXR)
NFY(CCAAT)
D
egr−1 / Egr
h1SMcG0017306
egr−1 connectivity
Egr mot. enr.
soxP−1 / HMG(Sox)
h1SMcG0006253
soxP−1 connectivity
HMG(Sox) mot. enr.
foxF1 / Forkhead_1
h1SMcG0016896
foxF1 connectivity
Forkhead_1 mot. enr.
mitfl−1 / bHLH (MITF/USF)
h1SMcG0009686
mitfl−1 connectivity
bHLH (MITF/USF) mot. enr.
coe / EBF
h1SMcG0016003
coe connectivity
EBF mot. enr.
dll−1 / Homeobox(DLX/En/LHX)
h1SMcG0002500
dll−1 connectivity
Homeobox(DLX/En/LHX) mot. enr.
ascl−2 / bHLH_3
h1SMcG0016534
ascl−2 connectivity
bHLH_3 mot. enr.
atoh8−2 / bHLH_1
h1SMcG0001060
atoh8−2 connectivity
bHLH_1 mot. enr.
foxa1 / NF1:FOXA1
h1SMcG0000282
foxa1 connectivity
NF1:FOXA1 mot. enr.

Supplementary Figure 9. A: Schematics of graph analysis. B: Distribution of gene-gene weighted correlation values of the whole co-expression WGCNA data. X axis, values of TOM matrix. Y axis, number of edges. Y axis is floored to 10,000. C: Distribution of gene-gene weighted correlation values above 0.1. Coloured lines indicate candidate thresholds for downstream graph analysis. D: Number of connected components per thresholds. E: Median degree (median number of neighbours per gene) per thresholds. F: Median number of genes in each connected component per threshold. G: Boxplot of number of genes in each connected component per threshold. Centre line, median; box limits, upper and lower quartiles; whiskers, 1.5x interquartile range; points, all data points. H: Number of genes on each graph per graph threshold. I: Stacked bar plot of fractions of genes from each WGCNA module per connected component. J: Correlation between TF intra-modular connectivity and TF centrality. (Left) scatter plot of relative TF intra-modular connectivity and relative TF centrality. Every dot is a TF. Colour code indicates module membership. (Right) jitter plot of Pearson correlation between TF connectivity and TF centrality all together without considering modules, or module-wise. Every dot is a module (or the whole network, in the case of the dot classified as “whole network”). K: Module-wise graph connecting modules (nodes) based on the number of genes between the two with high weighted correlation. Edge thickness indicates the number of cross connections. L: Module-wise graph connecting modules based on Spearman correlation of motif enrichment. M: Module-wise graph connecting modules based on similarity of functional category (COG) enrichment. N: Module-wise graph connecting modules based on similarity of TF connectivity. O: Aggregated module-wise graph, where edge thickness indicates number of times a pair of modules was connected in the aforementioned module-wise graphs. P: Fruchterman-Reingold projection of the WGCNA graph, values >0.35. Every dot is a gene. Colour indicates module membership.

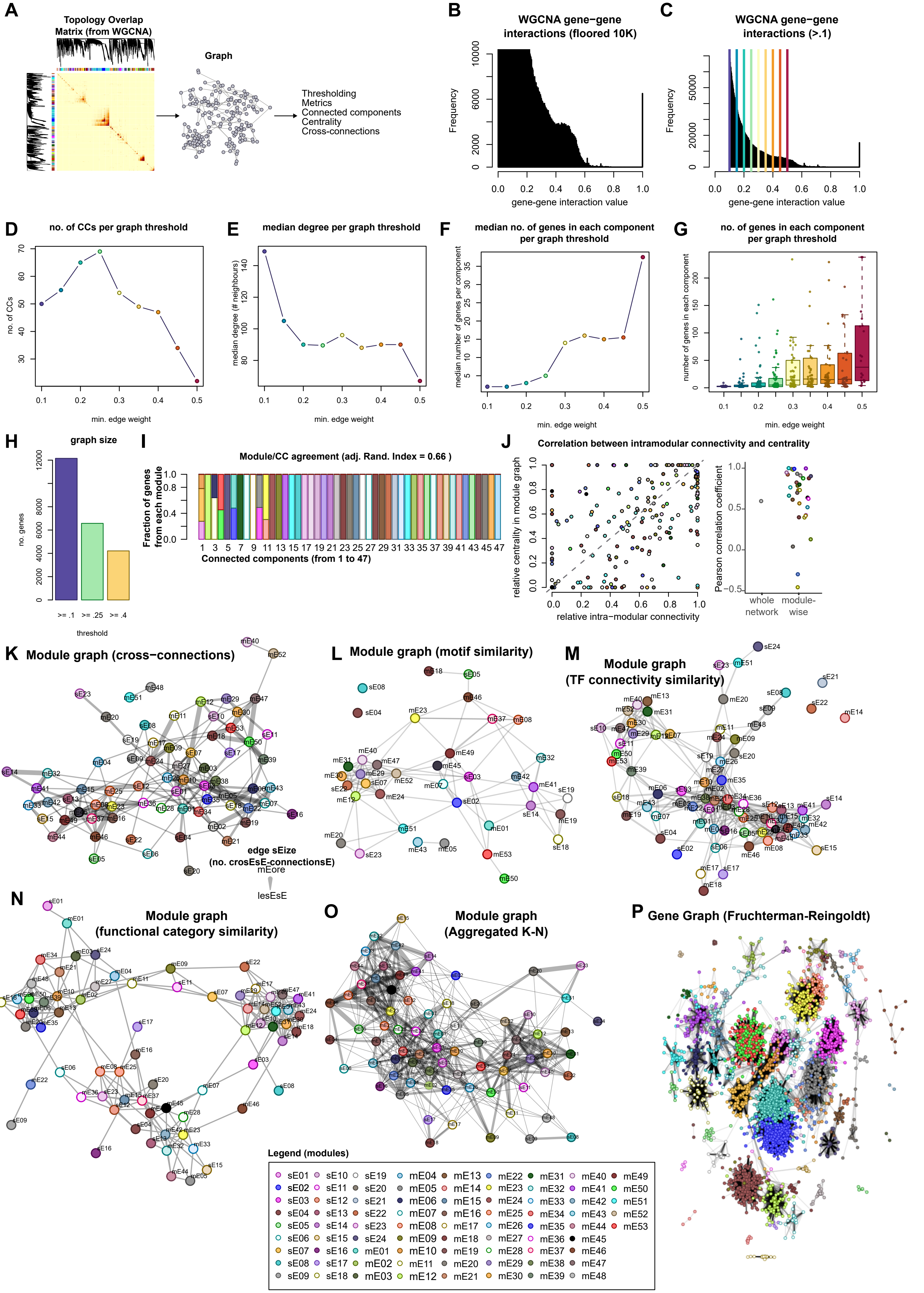

A
Topology Overlap Matrix (from WGCNA)
Graph
Thresholding
Metrics
Connected components
Centrality
Cross-connections
B
WGCNA gene−gene interactions (floored 10K)
Frequency
gene-gene interaction value
C
WGCNA gene−gene interactions (>.1)
Frequency
gene-gene interaction value
D
no. of CCs per graph threshold
no. of CCs
min. edge weight
E
median degree per graph threshold
median degree (# neighbours)
min. edge weight
F
median no. of genes in each component per graph threshold
median number of genes per component
min. edge weight
G
no. of genes in each component per graph threshold
number of genes in each component
min. edge weight
H
graph size
no. genes
>= .1
>= .25
>= .4
threshold
I
Module/CC agreement (adj. Rand. Index = 0.66)
Fraction of genes from each module
Connected components (from 1 to 47)
J
Correlation between intramodular connectivity and centrality
relative centrality in module graph
relative intra−modular connectivity
Pearson correlation coefficient
whole network
module-wise
K
Module graph (cross−connections)
edge sEize
(no. crosEsE-connectionsE)
mEore
lesEsE
L
Module graph (motif similarity)
M
Module graph
(TF connectivity similarity)
N
Module graph
(functional category similarity)
O
Module graph
(Aggregated K-N)
P
Gene Graph (Fruchterman-Reingoldt)
Legend (modules)
sE01 sE02 sE03 sE04 sE05 sE06 sE07 sE08 sE09
sE10 sE11 sE12 sE13 sE14 sE15 sE16 sE17 sE18
sE19 sE20 sE21 sE22 sE23 sE24 mE01 mE02 mE03
mE04 mE05 mE06 mE07 mE08 mE09 mE10 mE11 mE12
mE13 mE14 mE15 mE16 mE17 mE18 mE19 mE20 mE21
mE22 mE23 mE24 mE25 mE26 mE27 mE28 mE29 mE30
mE31 mE32 mE33 mE34 mE35 mE36 mE37 mE38 mE39
mE40 mE41 mE42 mE43 mE44 mE45 mE46 mE47 mE48
mE49 mE50 mE51 mE52 mE53

Supplementary Figure 10. A: Cell type similarity based on co-occurrency (Pearson correlation with bootstrapping) of OCR accessibility across all differentiated cell type (one-vs-neoblast) differential OCRs. B: (Left) Scale-free topology model fit of different networks obtained from raising the gene-wise correlations of the pseudobulk scATAC-seq data to fifteen different soft power thresholds. (Right) Mean connectivity of different networks obtained from raising the gene-wise correlations of the pseudobulk scATAC-seq data (one-vs-neoblast OCRs) to fifteen different soft power thresholds. C: Dendrogram of modules of co-accessibility. D: Heatmap of chromatin accessibility of OCRs from all the WGCNA co-accessibility modules across broad cell types in *S. mediterranea*. On the left side, stacked bar plot of average chromatin accessibility of each co-accessibility module in each broad cell type (see Methods; Supplementary Note 1), and bar plot showing number of OCRs per co-accessibility module. OCRs in rows, cell types in columns. A random subset of twenty OCRs per module is shown. On the right of this heatmap, a heatmap of gene expression of genes associated to OCRs from the co-accessibility modules. Genes in rows, cell types in columns. A subset of top-correlating genes is shown (see Supplementary Note 1 for details). Columns in both heatmaps clustered following the co-occurrence tree as in A. E: Box plot showing correlation of genes to the co-accessibility profile of modules from their associated OCRs. Every box corresponds to a co-accessibility module. Every point is a gene. Genes labelled in grey do not appear in D (nor Figure 3A). Genes labelled in red do. Centre line, median; box limits, upper and lower quartiles; whiskers, 1.5x interquartile range; points, data points. F: graph of association between co-expression modules (Figure 2) and co-accessibility modules (Figure 3) based on module membership of OCRs and module membership of their closest associated genes. Edge width indicates number of OCR-gene pairs in common. G: graph of association between co-expression modules (Figure 2) and co-accessibility modules (Figure 3) based on module membership enrichment of OCRs and module membership of their closest associated genes. Edge width indicates p-value of upper-tail hypergeometric test ($p < 0.05$, observed versus expected). H-P: Scatter plot showing the connectivity of different TFs to modules of co-accessibility, in relation to the motif enrichment of the same TF class in co-accessibility modules. Dot size indicates significance of hypergeometric test (q-value < 0.1). H: *p53*, I: *soxP-3*, J: *nkx2-2*, K: *gata456-1*, L: *meis-2*, M: *pou2/3-1*, N: *ets-1*, O: *cux-2*, P: *nf-YB-2*.

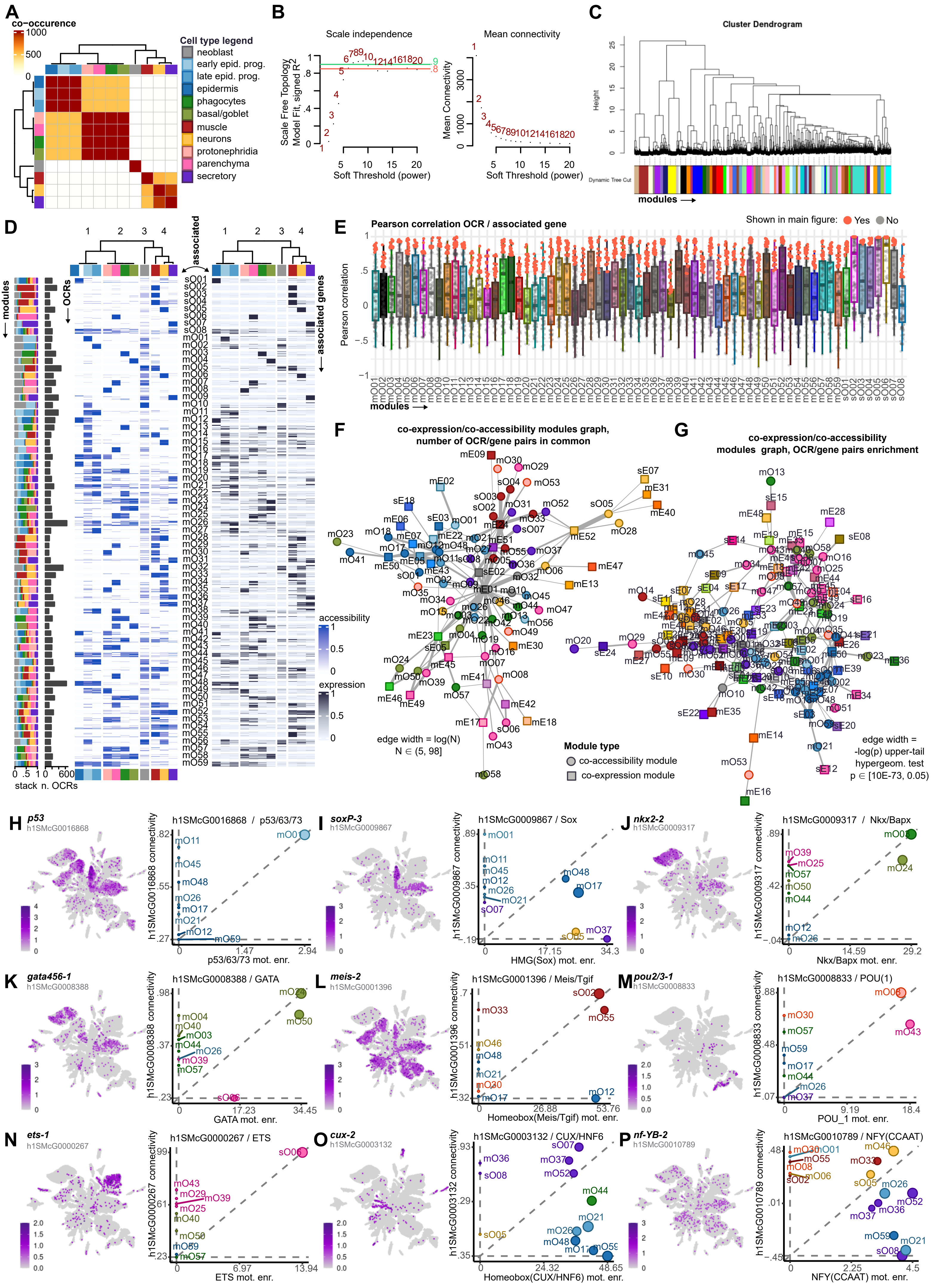
A
co−occurence
Cell type legend
neoblast
early epid. prog.
late epid. prog.
epidermis
phagocytes
basal/goblet
muscle
neurons
protonephridia
parenchyma
secretory
B
Scale independence
Scale Free Topology Model Fit, signed R^2
Soft Threshold (power)
Mean connectivity
Mean Connectivity
C
Cluster Dendrogram
Height
Dynamic Tree Cut
modules
D
modules
OCRs
associated
associated genes
accessibility
expression
stack n. OCRs
E
Pearson correlation OCR / associated gene
Shown in main figure: Yes No
Pearson correlation
modules
F
co-expression/co-accessibility modules graph, number of OCR/gene pairs in common
edge width = log(N)
N ∈ (5, 98]
Module type
co-accessibility module
co-expression module
G
co-expression/co-accessibility modules graph, OCR/gene pairs enrichment
edge width = -log(p) upper-tail hypergeom. test
p ∈ [10E-73, 0.05)
H
p53
h1SMcG0016868
h1SMcG0016868 / p53/63/73
h1SMcG0016868 connectivity
p53/63/73 mot. enr.
I
soxP-3
h1SMcG0009867
h1SMcG0009867 / Sox
h1SMcG0009867 connectivity
HMG(Sox) mot. enr.
J
nkx2-2
h1SMcG0009317
h1SMcG0009317 / Nkx/Bapx
h1SMcG0009317 connectivity
Nkx/Bapx mot. enr.
K
gata456-1
h1SMcG0008388
h1SMcG0008388 / GATA
h1SMcG0008388 connectivity
GATA mot. enr.
L
meis-2
h1SMcG0001396
h1SMcG0001396 / Meis/Tgif
h1SMcG0001396 connectivity
Homeobox(Meis/Tgif) mot. enr.
M
pou2/3-1
h1SMcG0008833
h1SMcG0008833 / POU(1)
h1SMcG0008833 connectivity
POU_1 mot. enr.
N
ets-1
h1SMcG0000267
h1SMcG0000267 / ETS
h1SMcG0000267 connectivity
ETS mot. enr.
O
cux-2
h1SMcG0003132
h1SMcG0003132 / CUX/HNF6
h1SMcG0003132 connectivity
Homeobox(CUX/HNF6) mot. enr.
P
nf-YB-2
h1SMcG0010789
h1SMcG0010789 / NFY(CCAAT)
h1SMcG0010789 connectivity
NFY(CCAAT) mot. enr.

Supplementary Figure 11. Whole motif enrichment analysis on the OCRs from each WGCNA module of OCR co-accessibility.

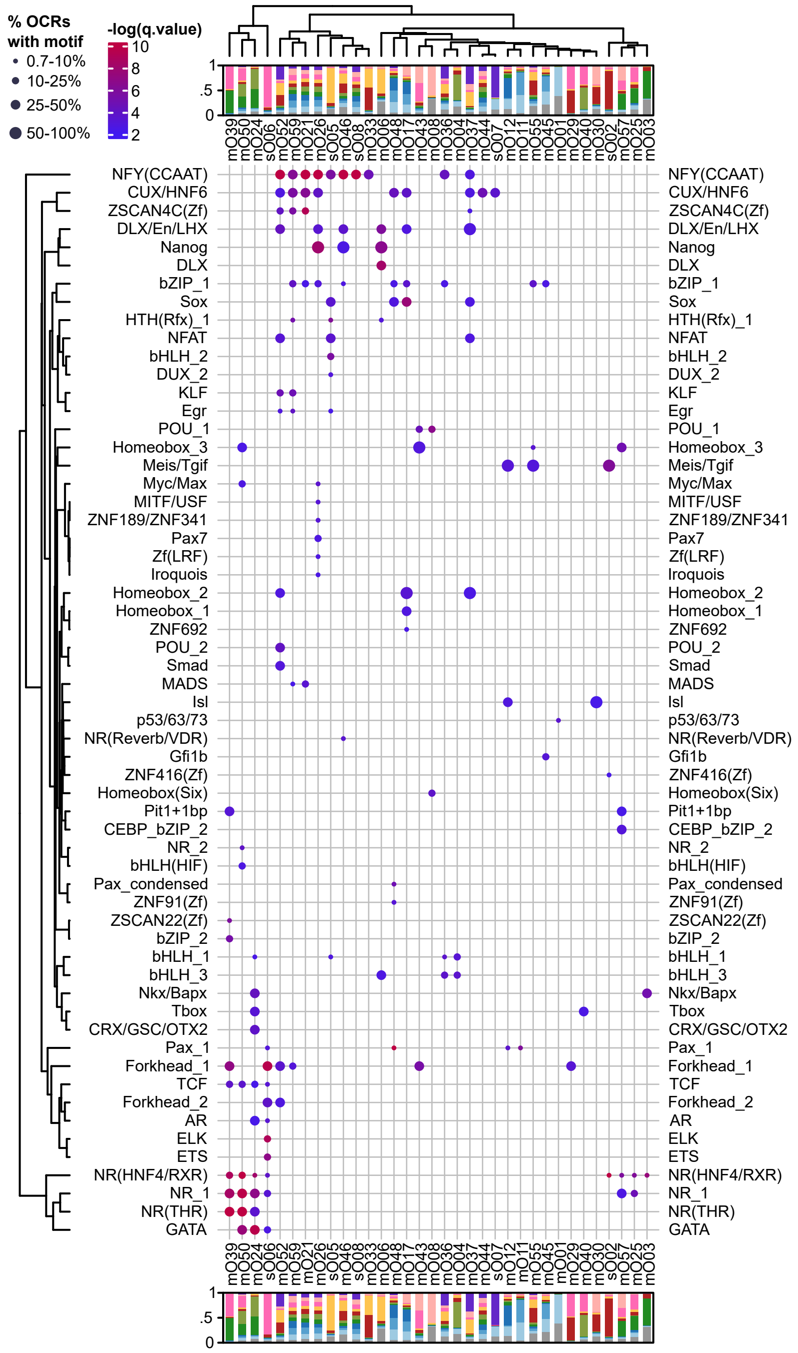

% OCRs with motif
0.7-10%
10-25%
25-50%
50-100%
-log(q.value)
10
8
6
4
2
mO39 mO50 mO24 sO06 mO52 mO59 mO21 mO26 sO05 mO46 sO08 mO33 mO06 mO48 mO17 mO43 mO08 mO36 mO04 mO37 mO44 sO07 mO12 mO11 mO55 mO45 mO01 mO29 mO40 mO30 sO02 mO57 mO25 mO03
NFY(CCAAT)
CUX/HNF6
ZSCAN4C(Zf)
DLX/En/LHX
Nanog
DLX
bZIP_1
Sox
HTH(Rfx)_1
NFAT
bHLH_2
DUX_2
KLF
Egr
POU_1
Homeobox_3
Meis/Tgif
Myc/Max
MITF/USF
ZNF189/ZNF341
Pax7
Zf(LRF)
Iroquois
Homeobox_2
Homeobox_1
ZNF692
POU_2
Smad
MADS
Isl
p53/63/73
NR(Reverb/VDR)
Gfi1b
ZNF416(Zf)
Homeobox(Six)
Pit1+1bp
CEBP_bZIP_2
NR_2
bHLH(HIF)
Pax_condensed
ZNF91(Zf)
ZSCAN22(Zf)
bZIP_2
bHLH_1
bHLH_3
Nkx/Bapx
Tbox
CRX/GSC/OTX2
Pax_1
Forkhead_1
TCF
Forkhead_2
AR
ELK
ETS
NR(HNF4/RXR)
NR_1
NR(THR)
GATA

Supplementary Figure 12. A: Tree of species used to transfer TF and motif annotation to *S. mediterranea* (bold) from a set of reference species (red) using automated orthology. B: Number of active TFs in each of the constructed graphs. C: Schematics of TF centrality and correlation. D: Heatmap of TF centrality in each of the graphs. TFs in columns, cell type graphs in rows. TFs have been sorted by highest value of centrality. E: Pearson correlation of cell types based on profile of TF centrality across networks.

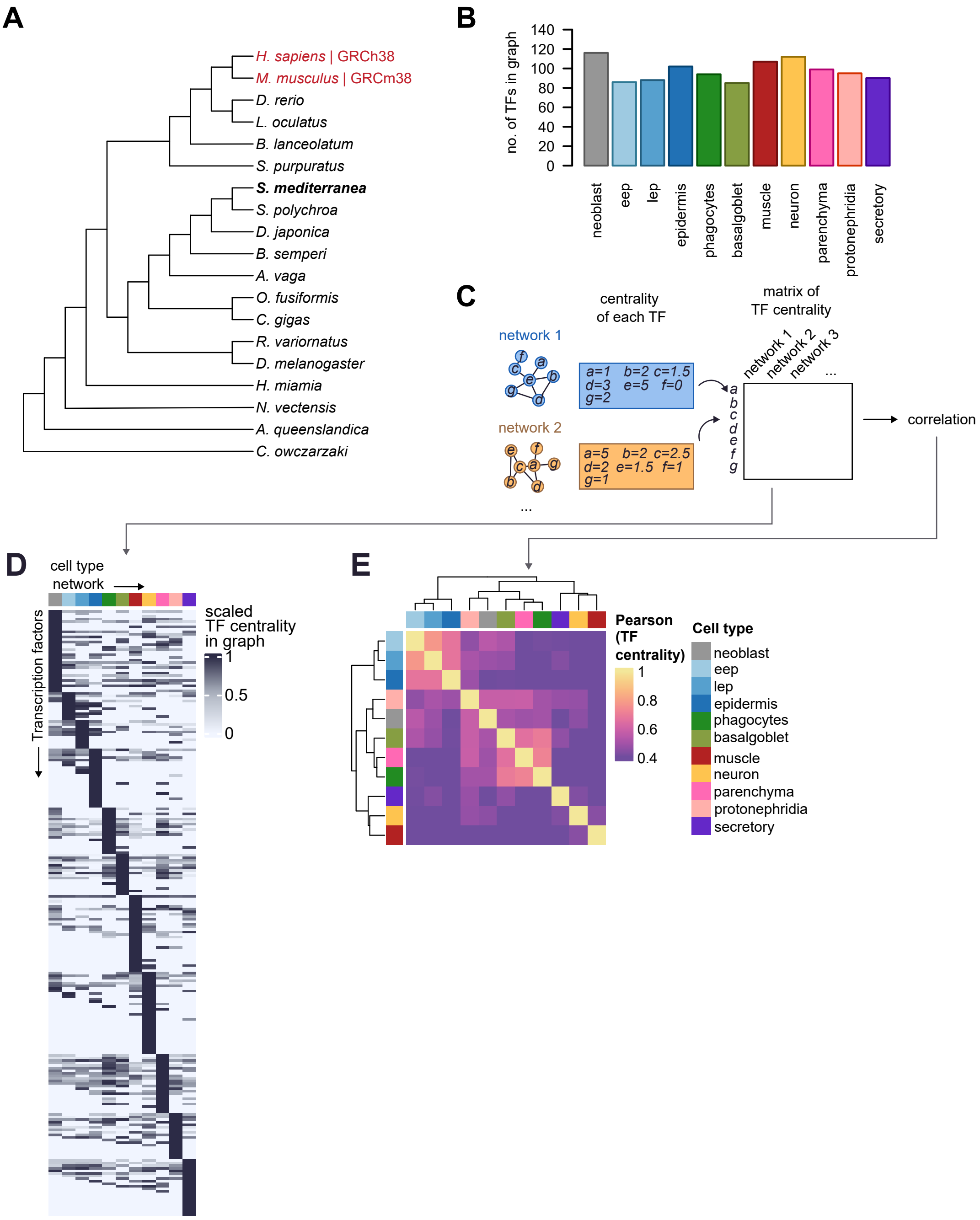

A
H. sapiens | GRCh38
M. musculus | GRCm38
D. rerio
L. oculatus
B. lanceolatum
S. purpuratus
S. mediterranea
S. polychroa
D. japonica
B. semperi
A. vaga
O. fusiformis
C. gigas
R. variornatus
D. melanogaster
H. miamia
N. vectensis
A. queenslandica
C. owczarzaki
B
no. of TFs in graph
140
120
100
80
60
40
20
0
neoblast
eep
lep
epidermis
phagocytes
basalgoblet
muscle
neuron
parenchyma
protonephridia
secretory
C
centrality of each TF
matrix of TF centrality
network 1
a=1 b=2 c=1.5 d=3 e=5 f=0 g=2
network 2
a=5 b=2 c=2.5 d=2 e=1.5 f=1 g=1
network 1 network 2 network 3 ...
a b c d e f g
correlation
D
cell type network
Transcription factors
scaled TF centrality in graph
1
0.5
0
E
Pearson (TF centrality)
1
0.8
0.6
0.4
Cell type
neoblast
eep
lep
epidermis
phagocytes
basalgoblet
muscle
neuron
parenchyma
protonephridia
secretory

Supplementary Figure 13. A: Volcano plots showing log fold change (x-axis) and -log p. adjusted (y axis) of each of the differentiated cell type (one-vs-neoblasts) differential gene expression analysis, used as input for ANANSE influence. B-J: Scatter plot of TF fold change and influence score for the top factors of each transition from neoblast to a non-pluripotent cell type: (B) early epidermal progenitors, (C) epidermis, (D) phagocytes, (E) basal/goblet, (F) muscle, (G) neurons, (H) parenchyma, (I) protonephridia, (J) secretory. Labels shown for the 20 TFs with highest influence score. K: Heatmap of TF influence score across cell fates. TFs in columns, cell fates in rows. Top tree: hierarchical clustering of TFs based on their co-influence across cell fates. L: Boxplots of influence score profiles of each of the clusters of co-influential TFs. Centre line, median; box limits, upper and lower quartiles; whiskers, 1.5x interquartile range; points, outliers. M: Graph of Pearson correlation between co-influential TFs.

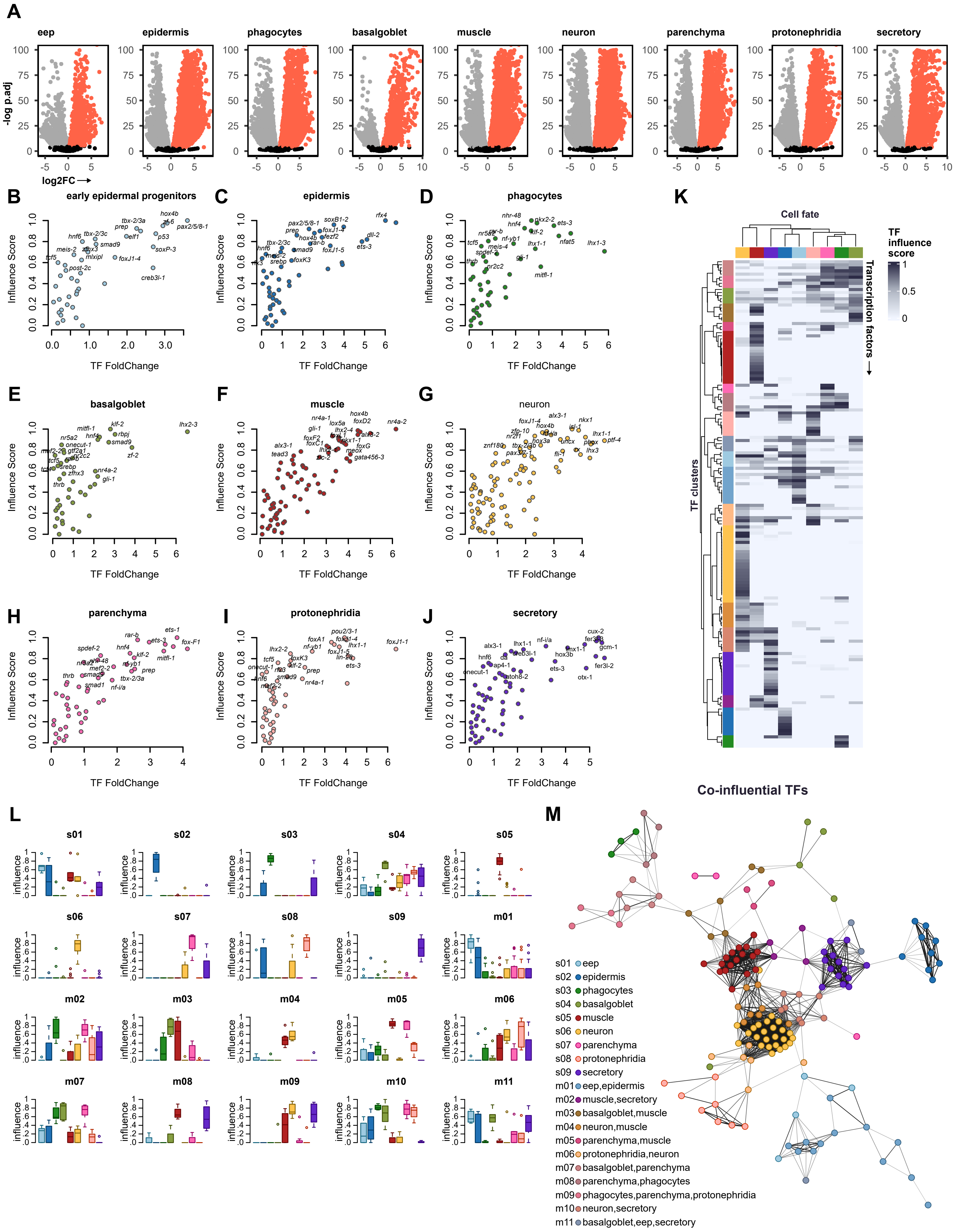
A
eep
epidermis
phagocytes
basalgoblet
muscle
neuron
parenchyma
protonephridia
secretory
-log p.adj
log2FC
B
early epidermal progenitors
C
epidermis
D
phagocytes
E
basalgoblet
F
muscle
G
neuron
H
parenchyma
I
protonephridia
J
secretory
Influence Score
TF FoldChange
K
Cell fate
TF influence score
Transcription factors
TF clusters
L
s01
s02
s03
s04
s05
s06
s07
s08
s09
m01
m02
m03
m04
m05
m06
m07
m08
m09
m10
m11
influence
Co-influential TFs
M
s01 eep
s02 epidermis
s03 phagocytes
s04 basalgoblet
s05 muscle
s06 neuron
s07 parenchyma
s08 protonephridia
s09 secretory
m01 eep,epidermis
m02 muscle,secretory
m03 basalgoblet,muscle
m04 neuron,muscle
m05 parenchyma,muscle
m06 protonephridia,neuron
m07 basalgoblet,parenchyma
m08 parenchyma,phagocytes
m09 phagocytes,parenchyma,protonephridia
m10 neuron,secretory
m11 basalgoblet,eep,secretory

Supplementary Figure 14. A-I: (left) differential network and (right) top target genes (top 5% interaction score values) of two example TFs of the differential network when comparing neoblasts to (A) early epidermal progenitors, (B) epidermis, (C) phagocytes, (D) basal/goblet cells, (E) muscle cells, (F) neurons, (G) parenchymal cells, (H) protonephridia cells, and (I) secretory cells. Colour intensity of TFs in networks indicates relative outdegree (fraction of emitting connections). Outlined dots in strip charts indicate the position of the labelled genes J: Scatter plot showing influence scores of different TFs for the transitions from neoblast to phagocytes and from neoblasts to parenchyma, showing some TFs have high influence scores in more than one cell type.

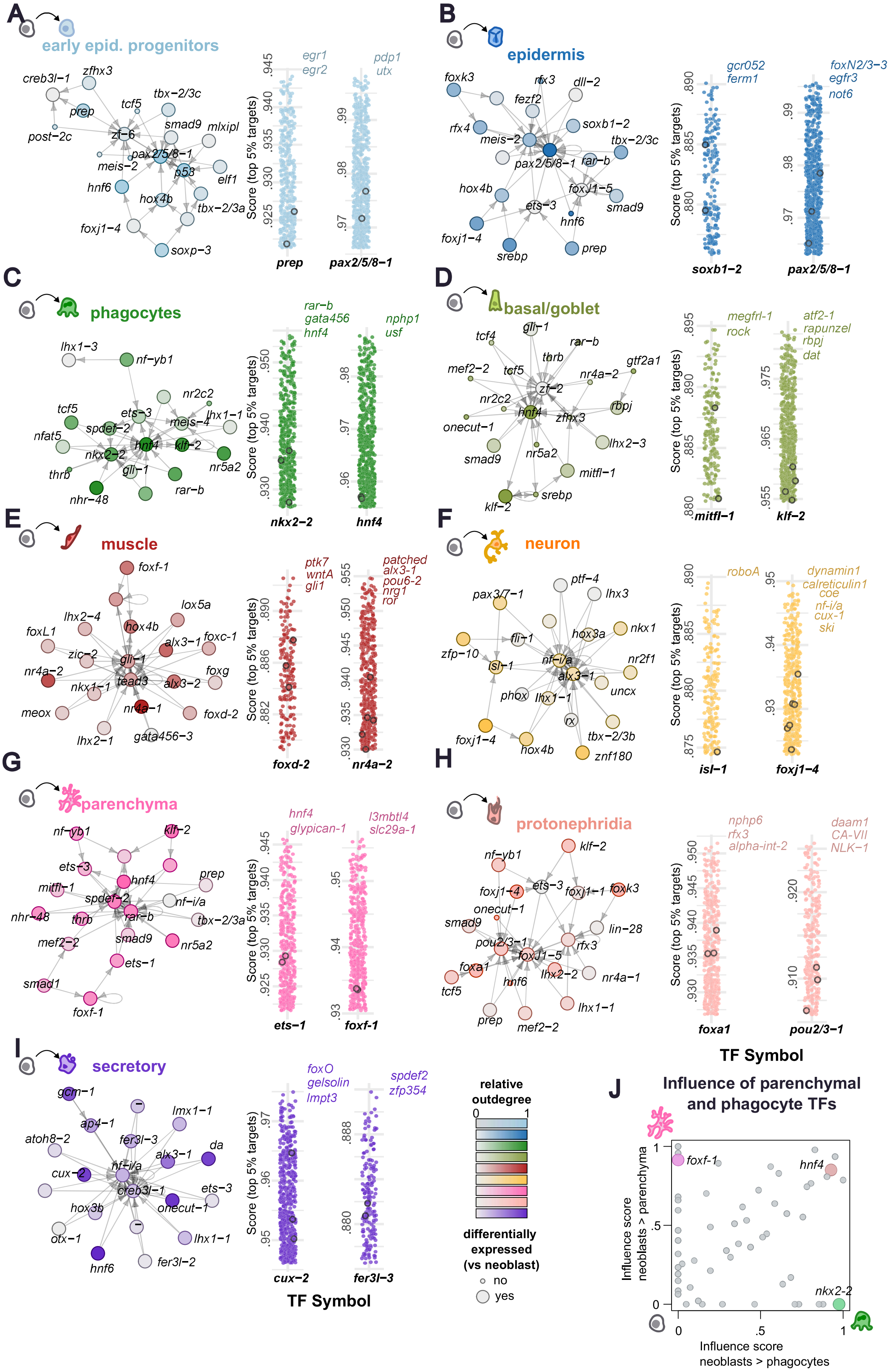

A
early epid. progenitors
prep
pax2/5/8-1
B
epidermis
soxb1-2
pax2/5/8-1
C
phagocytes
nkx2-2
hnf4
D
basal/goblet
mitfl-1
klf-2
E
muscle
foxd-2
nr4a-2
F
neuron
isl-1
foxj1-4
G
parenchyma
ets-1
foxf-1
H
protonephridia
foxa1
pou2/3-1
TF Symbol
I
secretory
cux-2
fer3l-3
TF Symbol
relative outdegree
differentially expressed (vs neoblast)
no
yes
J
Influence of parenchymal and phagocyte TFs
Influence score neoblasts > parenchyma
Influence score neoblasts > phagocytes

Supplementary Figure 15. A: Heatmap showing expression of differentially expressed genes in the *pax2/5/8-1* knock-down animals, re-analysis of data from Cheng *et al*., 2018. B: Heatmap showing expression of differentially expressed genes in the *p53* knock-down animals, re-analysis of data from Tu *et al*., 2015. C: Heatmap showing expression of differentially expressed genes in the *soxP-3* knock-down animals, re-analysis of data from Cheng *et al*., 2018. D: normalised gene score of *pax2/5/8-1(RNAi)* downregulated genes across all broad cell types in our single cell dataset. Centre line, median; box limits, upper and lower quartiles; whiskers, 1.5x interquartile range; points, subsample of data points for visualisation. E: normalised gene score of *p53(RNAi)* downregulated genes across all broad cell types in our single cell dataset. Centre line, median; box limits, upper and lower quartiles; whiskers, 1.5x interquartile range; points, subsample of data points for visualisation. F: normalised gene score of *soxP-3(RNAi)* downregulated genes across all broad cell types in our single cell dataset. Centre line, median; box limits, upper and lower quartiles; whiskers, 1.5x interquartile range; points, subsample of data points for visualisation. G: Box plots showing the predicted ANANSE weighted binding between *pax2/5/8-1* and target genes. Centre line, median; box limits, upper and lower quartiles; whiskers, 1.5x interquartile range; points, data points. H: Box plots showing the predicted ANANSE weighted binding between *p53* and target genes. Centre line, median; box limits, upper and lower quartiles; whiskers, 1.5x interquartile range; points, data points. I: Box plots showing the predicted ANANSE weighted binding between *soxP-3* and target genes. Centre line, median; box limits, upper and lower quartiles; whiskers, 1.5x interquartile range; points, data points.

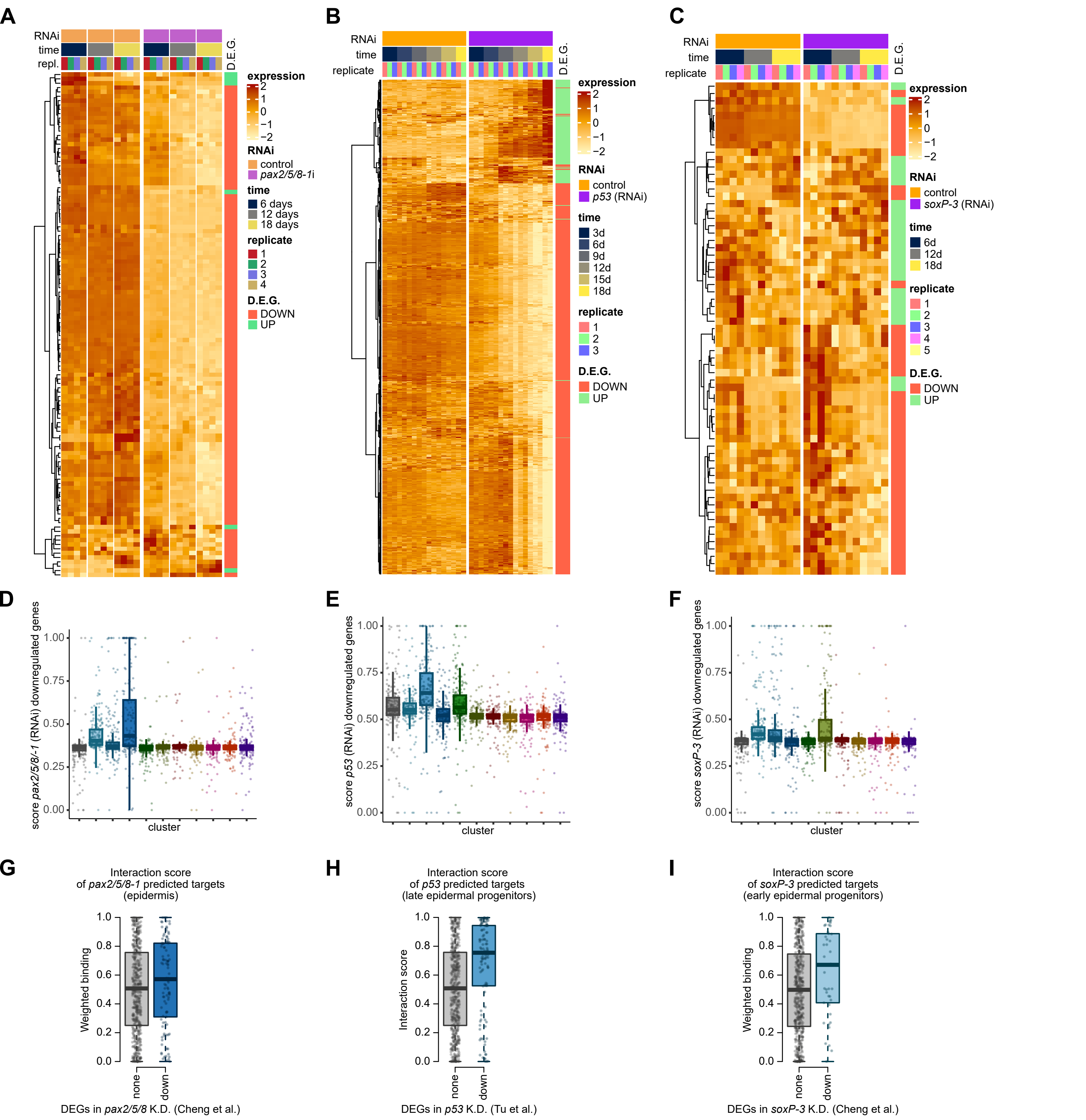
A
RNAi
time
repl.
D.E.G.
expression
RNAi
control
pax2/5/8-1i
time
6 days
12 days
18 days
replicate
D.E.G.
DOWN
UP
B
RNAi
time
replicate
control
p53 (RNAi)
3d
6d
9d
12d
15d
18d
C
control
soxP-3 (RNAi)
6d
12d
18d
D
score pax2/5/8-1 (RNAi) downregulated genes
cluster
E
score p53 (RNAi) downregulated genes
F
score soxP-3 (RNAi) downregulated genes
G
Interaction score
of pax2/5/8-1 predicted targets
(epidermis)
Weighted binding
none
down
DEGs in pax2/5/8 K.D. (Cheng et al.)
H
Interaction score
of p53 predicted targets
(late epidermal progenitors)
Interaction score
DEGs in p53 K.D. (Tu et al.)
I
Interaction score
of soxP-3 predicted targets
(early epidermal progenitors)
DEGs in soxP-3 K.D. (Cheng et al.)

Supplementary Figure 16. A: heatmap of correlation between samples. B: Volcano plot showing differentially expressed genes (re-analysis of data from Cowles *et al*., 2014). Dashed lines indicate fold change (vertical) and adjusted p-value (horizontal) thresholds for significance (Wald test). C: Heatmap showing expression of differentially expressed genes across samples. D: Heatmap showing anteroposterior expression of differentially expressed genes. E: Normalised gene score of *coe(RNAi)* downregulated genes across all cell types in our single cell dataset. Centre line, median; box limits, upper and lower quartiles; whiskers, 1.5x interquartile range; points, subsample of data points for visualisation. F: Box plots showing the predicted ANANSE interaction score and weighted binding between *coe* and target genes in the epidermis network. Centre line, median; box limits, upper and lower quartiles; whiskers, 1.5x interquartile range; points, data points. G: Box plots showing the predicted ANANSE weighted binding between *coe* and target genes in the neurons network. Centre line, median; box limits, upper and lower quartiles; whiskers, 1.5x interquartile range; points, data points.

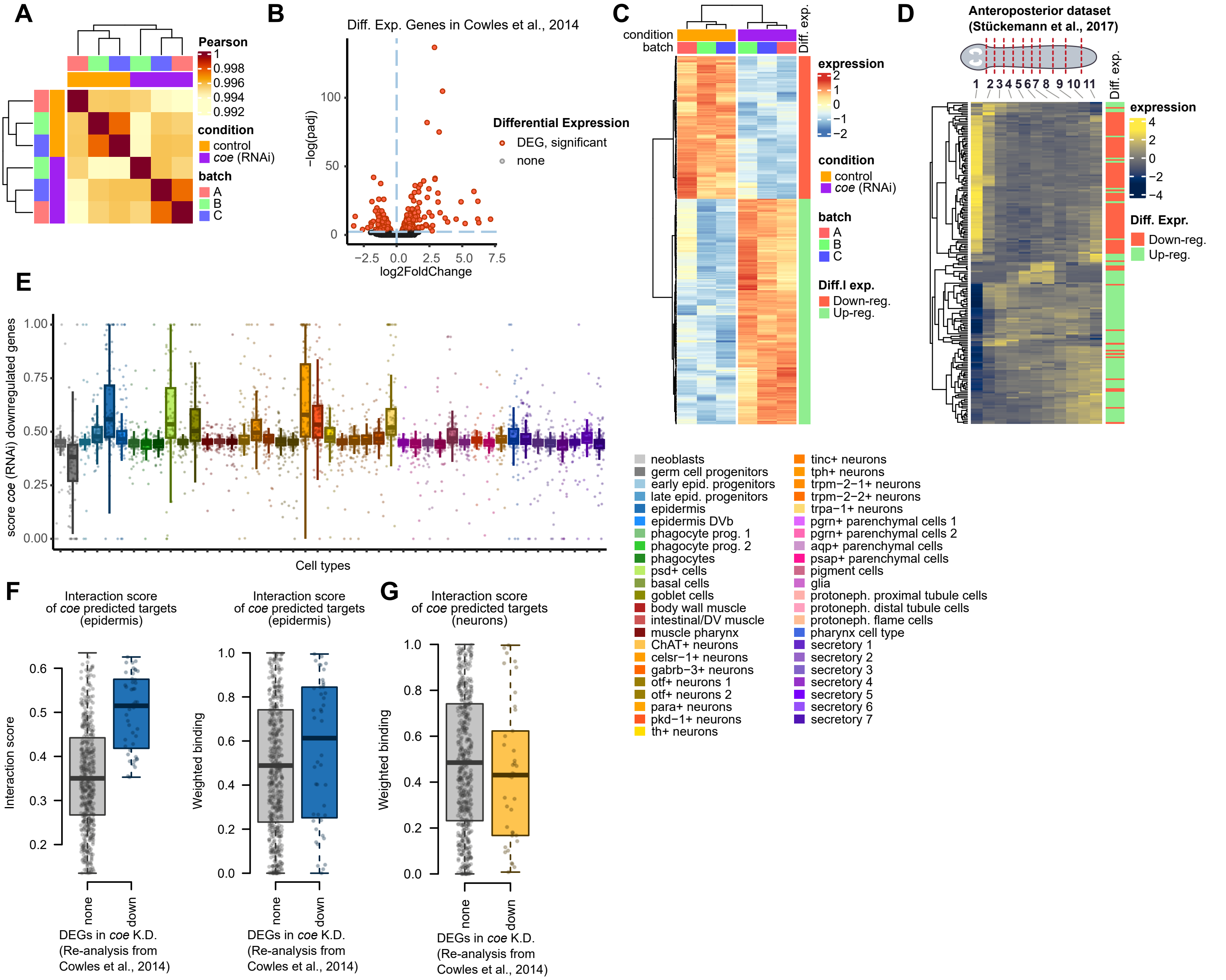
A
Pearson
1
0.998
0.996
0.994
0.992
condition
control
coe (RNAi)
batch
A
B
C
B
Diff. Exp. Genes in Cowles et al., 2014
−log(padj)
log2FoldChange
Differential Expression
DEG, significant
none
C
condition
batch
Diff. exp.
expression
condition
control
coe (RNAi)
batch
A
B
C
Diff.l exp.
Down-reg.
Up-reg.
D
Anteroposterior dataset
(Stückemann et al., 2017)
1 2 3 4 5 6 7 8 9 10 11
Diff. exp.
expression
Diff. Expr.
Down-reg.
Up-reg.
E
score coe (RNAi) downregulated genes
Cell types
neoblasts
germ cell progenitors
early epid. progenitors
late epid. progenitors
epidermis
epidermis DVb
phagocyte prog. 1
phagocyte prog. 2
phagocytes
psd+ cells
basal cells
goblet cells
body wall muscle
intestinal/DV muscle
muscle pharynx
ChAT+ neurons
celsr−1+ neurons
gabrb−3+ neurons
otf+ neurons 1
otf+ neurons 2
para+ neurons
pkd−1+ neurons
th+ neurons
tinc+ neurons
tph+ neurons
trpm−2−1+ neurons
trpm−2−2+ neurons
trpa−1+ neurons
pgrn+ parenchymal cells 1
pgrn+ parenchymal cells 2
aqp+ parenchymal cells
psap+ parenchymal cells
pigment cells
glia
protoneph. proximal tubule cells
protoneph. distal tubule cells
protoneph. flame cells
pharynx cell type
secretory 1
secretory 2
secretory 3
secretory 4
secretory 5
secretory 6
secretory 7
F
Interaction score of coe predicted targets (epidermis)
Interaction score
Weighted binding
none
down
DEGs in coe K.D. (Re-analysis from Cowles et al., 2014)
G
Interaction score of coe predicted targets (neurons)
Weighted binding
none
down
DEGs in coe K.D. (Re-analysis from Cowles et al., 2014)

Supplementary Figure 17. A: (Top) Scatter plot showing the quantile-normalised, log-transformed gene expression of control animals (x axis) and *prep* knockdown animals (y axis). (bottom) Fold change of gene expression between *prep* knockdown animals and control animals. Dashed lines indicate thresholds to define up- or down-regulation. B: Heatmap of gene expression showing antero-posterior expression of genes detected as up- or down-regulated in A. C: Normalised gene score of *prep(RNAi)* downregulated genes across all broad cell types in our single cell dataset. Centre line, median; box limits, upper and lower quartiles; whiskers, 1.5x interquartile range; points, subsample of data points for visualisation. D: Heatmap of gene expression showing antero-posterior expression of genes detected as top target of *prep* in epidermis according to our ANANSE network analysis. E: Box plots showing the predicted ANANSE interaction score between *prep* and target genes on the networks of cell types with high gene scores for *prep(RNAi)* down-regulated genes. Centre line, median; box limits, upper and lower quartiles; whiskers, 1.5x interquartile range; points, data points.

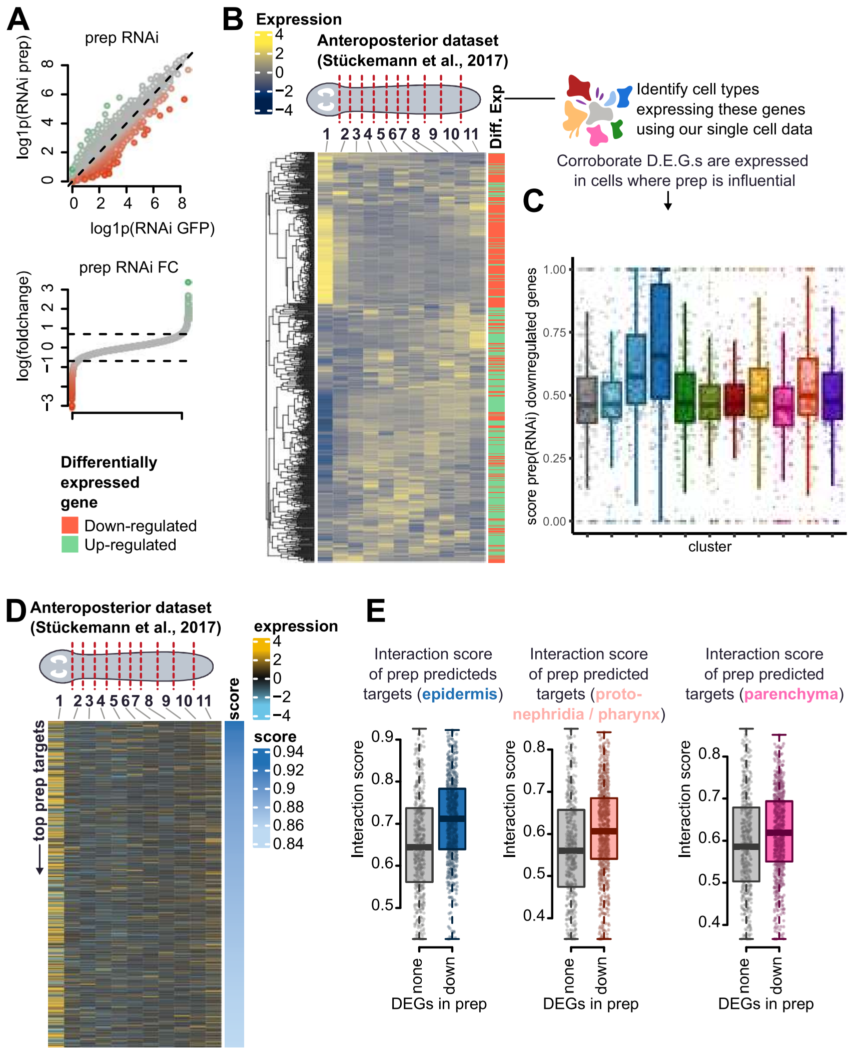
A
prep RNAi
log1p(RNAi prep)
log1p(RNAi GFP)
prep RNAi FC
log(foldchange)
Differentially expressed gene
Down-regulated
Up-regulated
B
Expression
Anteroposterior dataset (Stückemann et al., 2017)
1 2 3 4 5 6 7 8 9 10 11
Diff. Exp
Identify cell types expressing these genes using our single cell data
Corroborate D.E.G.s are expressed in cells where prep is influential
C
score prep(RNAi) downregulated genes
cluster
D
Anteroposterior dataset (Stückemann et al., 2017)
expression
score
top prep targets
E
Interaction score of prep predicteds targets (epidermis)
Interaction score of prep predicted targets (proto-nephridia / pharynx)
Interaction score of prep predicted targets (parenchyma)
Interaction score
none
down
DEGs in prep

Supplementary Figure 18. A: Feature plot showing expression of *alx3-1* in our single cell transcriptomics dataset. B: Heatmap of correlation of samples. C: Normalised gene score of *alx3-1(RNAi)* down-regulated genes across all broad cell types of our single cell dataset. Centre line, median; box limits, upper and lower quartiles; whiskers, 1.5x interquartile range; points, subsample of data points for visualisation. D: Box plots showing the predicted ANANSE weighted binding between *alx3-1* and target genes in the secretory, neurons, and muscle network. Centre line, median; box limits, upper and lower quartiles; whiskers, 1.5x interquartile range; points, data points. E: whole mount *in situ* hybridisation (WISH) of the gene h1SMcG0000140 in regenerating regions of control planarians and *alx3-1* knockdown planarians. Scale bar = 0.2 mm.

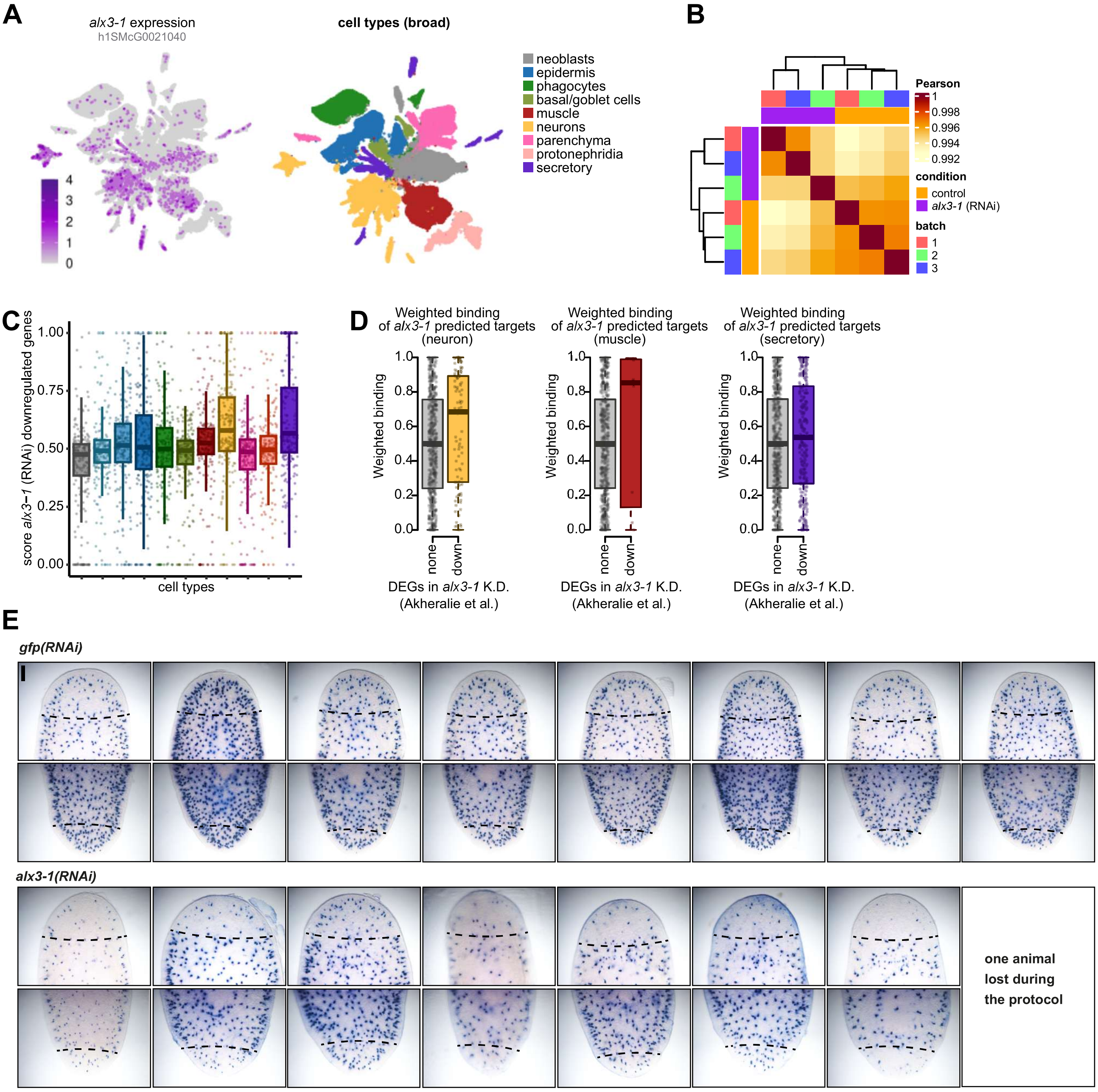
A
alx3-1 expression
h1SMcG0021040
4
3
2
1
0
cell types (broad)
neoblasts
epidermis
phagocytes
basal/goblet cells
muscle
neurons
parenchyma
protonephridia
secretory
B
Pearson
1
0.998
0.996
0.994
0.992
condition
control
alx3-1 (RNAi)
batch
1
2
3
C
score alx3-1 (RNAi) downregulated genes
1.00
0.75
0.50
0.25
0.00
cell types
D
Weighted binding of alx3-1 predicted targets (neuron)
Weighted binding of alx3-1 predicted targets (muscle)
Weighted binding of alx3-1 predicted targets (secretory)
Weighted binding
1.0
0.8
0.6
0.4
0.2
0.0
none
down
DEGs in alx3-1 K.D. (Akheralie et al.)
E
gfp(RNAi)
alx3-1(RNAi)
one animal lost during the protocol

Supplementary Figure 19. Phenotype progression of the *hnf4*i RNAi knock-down in *Schmidtea mediterranea*.

***hnf4*i RNAi phenotype progression**

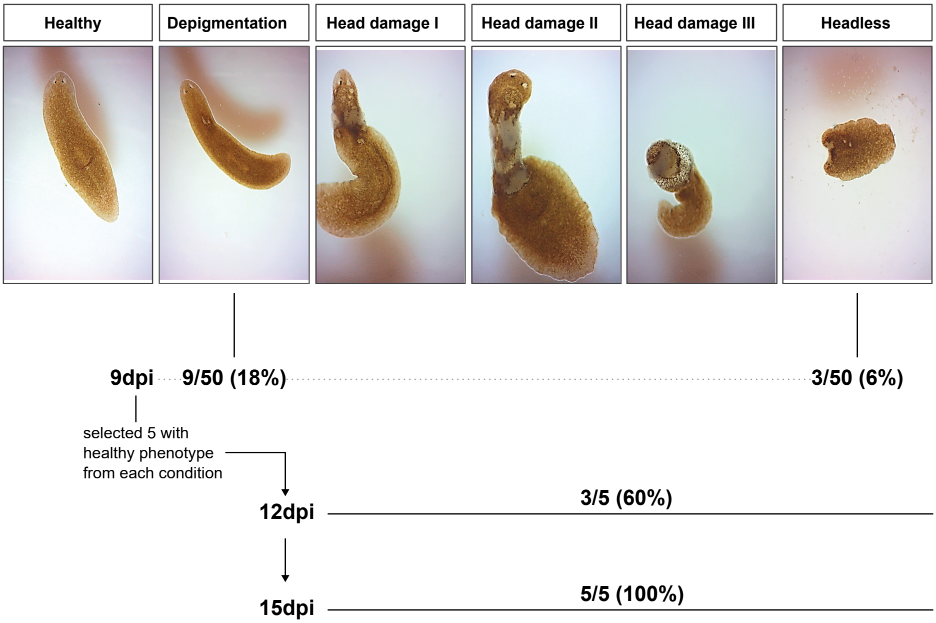

Supplementary Figure 20. A: Bipartite graph showing the transfer of labels from our whole scRNA-seq dataset to the *hnf4*i dataset. B: Graph showing which labels of our whole scRNA-seq dataset are received by more than one cluster from the *hnf4* knockdown single cell dataset. Of these, only cluster 14 contain cells that receive labels from more than one broad cell type (neoblasts and phagocyte progenitors). C: Violin plot of number of genes per cell on each cell type of the *hnf4* knockdown single cell dataset. D: Violin plot of number of counts per cell on each cell type of the *hnf4* knockdown single cell dataset. Points, data points. E: UMAP feature plots showing expression of *hnf4* in our *hnf4* knockdown single cell dataset. F: Wilcoxon p-values when testing for significant differences in the gene score of the markers of several cell types between cells from control and *hnf4(RNAi)* animals, for different cell types.

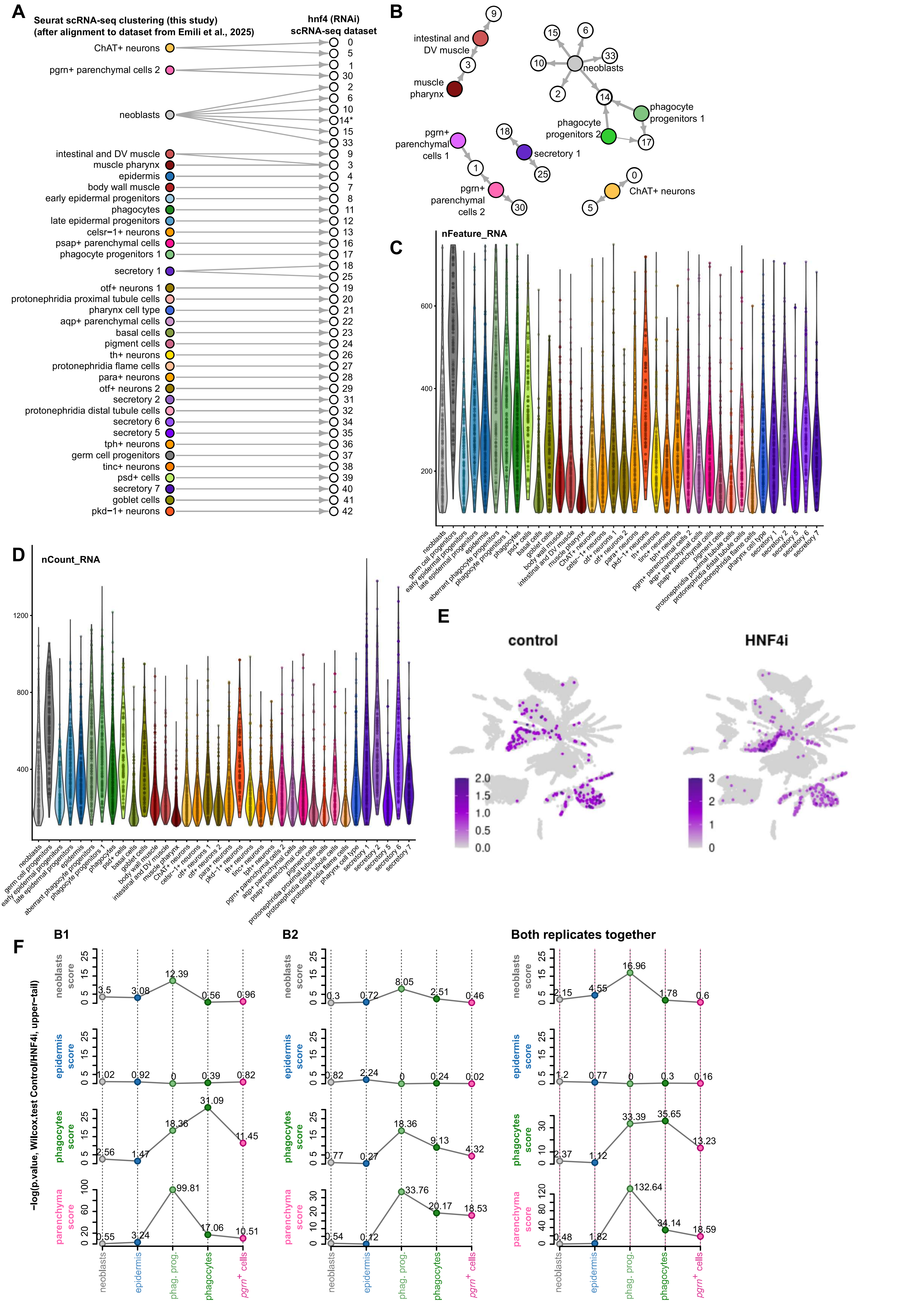

A
Seurat scRNA-seq clustering (this study)
(after alignment to dataset from Emili et al., 2025)
hnf4 (RNAi)
scRNA-seq dataset
ChAT+ neurons
pgrn+ parenchymal cells 2
neoblasts
intestinal and DV muscle
muscle pharynx
epidermis
body wall muscle
early epidermal progenitors
phagocytes
late epidermal progenitors
celsr−1+ neurons
psap+ parenchymal cells
phagocyte progenitors 1
secretory 1
otf+ neurons 1
protonephridia proximal tubule cells
pharynx cell type
aqp+ parenchymal cells
basal cells
pigment cells
th+ neurons
protonephridia flame cells
para+ neurons
otf+ neurons 2
secretory 2
protonephridia distal tubule cells
secretory 6
secretory 5
tph+ neurons
germ cell progenitors
tinc+ neurons
psd+ cells
secretory 7
goblet cells
pkd−1+ neurons
0
5
1
30
2
6
10
14*
15
33
9
3
4
7
8
11
12
13
16
17
18
25
19
20
21
22
23
24
26
27
28
29
31
32
34
35
36
37
38
39
40
41
42
B
intestinal and DV muscle
muscle pharynx
neoblasts
phagocyte progenitors 1
phagocyte progenitors 2
pgrn+ parenchymal cells 1
secretory 1
pgrn+ parenchymal cells 2
ChAT+ neurons
C
nFeature_RNA
D
nCount_RNA
E
control
HNF4i
F
B1
B2
Both replicates together
−log(p.value, Wilcox.test Control/HNF4i, upper−tail)
neoblasts score
epidermis score
phagocytes score
parenchyma score
neoblasts
epidermis
phag. prog.
phagocytes
pgrn+ cells

Supplementary Figure 21. A: Scatter plot showing the number of differentially expressed genes on each cell type (DEGs) in relation to cell type cluster size (number of cells). B: Scatter plot showing the number of DEGs on each cell type in relation to the level of expression of *hnf4* on each cell type. C-F: Bar plots of Gene Ontology Enrichment for the phagocytes-exclusive (C), parenchyma-exclusive (D), common (E) or all (F) DEGs. G: Logistic regression models of predicting a gene as down-regulated based on ANANSE interaction scores of *hnf4* and predicted targets, and the downregulation by knockdown of *hnf4*, for phagocytes (left) and parenchyma (right) networks. H: Feature plots of the expression of *nkx2-2*, *hnf4*, and *foxF-1* in our whole scRNA dataset. I: Connectivity of *nkx2-2*, *hnf4*, and *foxF-1* to the different WGCNA co-expression modules (see Figure 2).

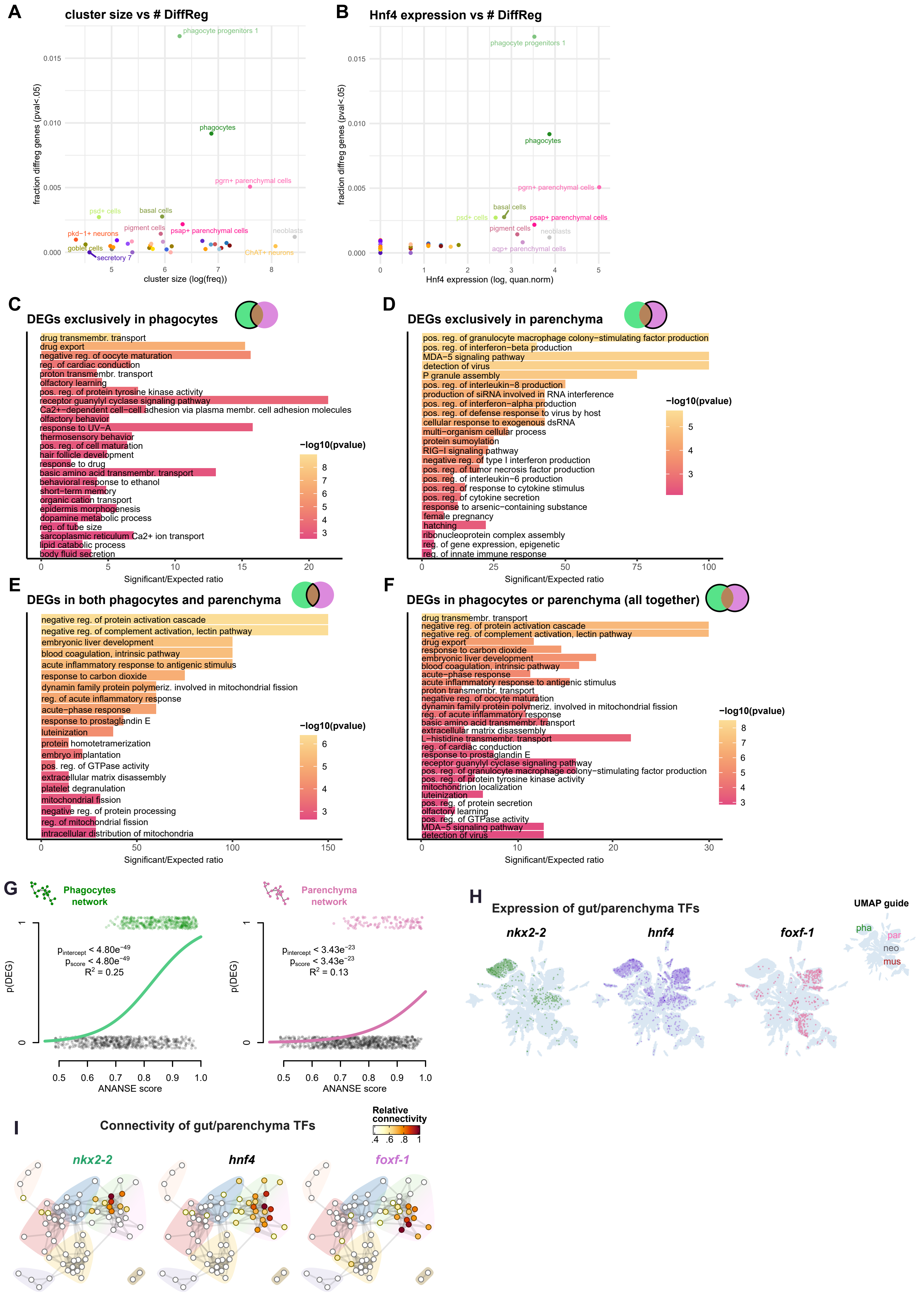

A
cluster size vs # DiffReg
fraction diffreg genes (pval<.05)
cluster size (log(freq))
phagocyte progenitors 1
phagocytes
pgrn+ parenchymal cells
psd+ cells
basal cells
pigment cells
psap+ parenchymal cells
neoblasts
pkd−1+ neurons
goblet cells
secretory 7
ChAT+ neurons
B
Hnf4 expression vs # DiffReg
fraction diffreg genes (pval<.05)
Hnf4 expression (log, quan.norm)
phagocyte progenitors 1
phagocytes
pgrn+ parenchymal cells
basal cells
psd+ cells
psap+ parenchymal cells
pigment cells
neoblasts
aqp+ parenchymal cells
C
DEGs exclusively in phagocytes
drug transmembr. transport
drug export
negative reg. of oocyte maturation
reg. of cardiac conduction
proton transmembr. transport
olfactory learning
pos. reg. of protein tyrosine kinase activity
receptor guanylyl cyclase signaling pathway
Ca2+−dependent cell−cell adhesion via plasma membr. cell adhesion molecules
olfactory behavior
response to UV−A
thermosensory behavior
pos. reg. of cell maturation
hair follicle development
response to drug
basic amino acid transmembr. transport
behavioral response to ethanol
short−term memory
organic cation transport
epidermis morphogenesis
dopamine metabolic process
reg. of tube size
sarcoplasmic reticulum Ca2+ ion transport
lipid catabolic process
body fluid secretion
−log10(pvalue)
Significant/Expected ratio
D
DEGs exclusively in parenchyma
pos. reg. of granulocyte macrophage colony−stimulating factor production
pos. reg. of interferon−beta production
MDA−5 signaling pathway
detection of virus
P granule assembly
pos. reg. of interleukin−8 production
production of siRNA involved in RNA interference
pos. reg. of interferon−alpha production
pos. reg. of defense response to virus by host
cellular response to exogenous dsRNA
multi−organism cellular process
protein sumoylation
RIG−I signaling pathway
negative reg. of type I interferon production
pos. reg. of tumor necrosis factor production
pos. reg. of interleukin−6 production
pos. reg. of response to cytokine stimulus
pos. reg. of cytokine secretion
response to arsenic−containing substance
female pregnancy
hatching
ribonucleoprotein complex assembly
reg. of gene expression, epigenetic
reg. of innate immune response
−log10(pvalue)
Significant/Expected ratio
E
DEGs in both phagocytes and parenchyma
negative reg. of protein activation cascade
negative reg. of complement activation, lectin pathway
embryonic liver development
blood coagulation, intrinsic pathway
acute inflammatory response to antigenic stimulus
response to carbon dioxide
dynamin family protein polymeriz. involved in mitochondrial fission
reg. of acute inflammatory response
acute−phase response
response to prostaglandin E
luteinization
protein homotetramerization
embryo implantation
pos. reg. of GTPase activity
extracellular matrix disassembly
platelet degranulation
mitochondrial fission
negative reg. of protein processing
reg. of mitochondrial fission
intracellular distribution of mitochondria
−log10(pvalue)
Significant/Expected ratio
F
DEGs in phagocytes or parenchyma (all together)
drug transmembr. transport
negative reg. of protein activation cascade
negative reg. of complement activation, lectin pathway
drug export
response to carbon dioxide
embryonic liver development
blood coagulation, intrinsic pathway
acute−phase response
acute inflammatory response to antigenic stimulus
proton transmembr. transport
negative reg. of oocyte maturation
dynamin family protein polymeriz. involved in mitochondrial fission
reg. of acute inflammatory response
basic amino acid transmembr. transport
extracellular matrix disassembly
L−histidine transmembr. transport
reg. of cardiac conduction
response to prostaglandin E
receptor guanylyl cyclase signaling pathway
pos. reg. of granulocyte macrophage colony−stimulating factor production
pos. reg. of protein tyrosine kinase activity
mitochondrion localization
luteinization
pos. reg. of protein secretion
olfactory learning
pos. reg. of GTPase activity
MDA−5 signaling pathway
detection of virus
−log10(pvalue)
Significant/Expected ratio
G
Phagocytes network
$p_{intercept} < 4.80e^{-49}$
$p_{score} < 4.80e^{-49}$
$R^2 = 0.25$
p(DEG)
ANANSE score
Parenchyma network
$p_{intercept} < 3.43e^{-23}$
$p_{score} < 3.43e^{-23}$
$R^2 = 0.13$
p(DEG)
ANANSE score
H
Expression of gut/parenchyma TFs
nkx2-2
hnf4
foxf-1
UMAP guide
pha
par
neo
mus
I
Connectivity of gut/parenchyma TFs
Relative connectivity
nkx2-2
hnf4
foxf-1

Supplementary Figure 22. A: phenotype curve of knock-down animals. Scale bar = 0.2mm. B: Phenotype of knockdown animals at twelve days post-injection. Scale bar = 0.2mm. C-U: normalised gene scores of the different sets of differentially expressed genes, across all the broad cell types in our single cell dataset. Centre line, median; box limits, upper and lower quartiles; whiskers, 1.5x interquartile range; points, subsample of data points for visualisation. V: Bar plot showing the number of low dose response genes detected in each set of overlapping DEGs across conditions. W: Bar plot showing the number of high dose response genes detected in each set of overlapping DEGs across conditions. X: Boxplot showing the ANANSE predicted weighted binding of nkx2-2 predicted targets in the phagocytes network. Centre line, median; box limits, upper and lower quartiles; whiskers, 1.5x interquartile range; points, data points. Y: Boxplot showing the ANANSE predicted weighted binding of foxF-1 predicted targets in the parenchyma network. Centre line, median; box limits, upper and lower quartiles; whiskers, 1.5x interquartile range; points, data points.

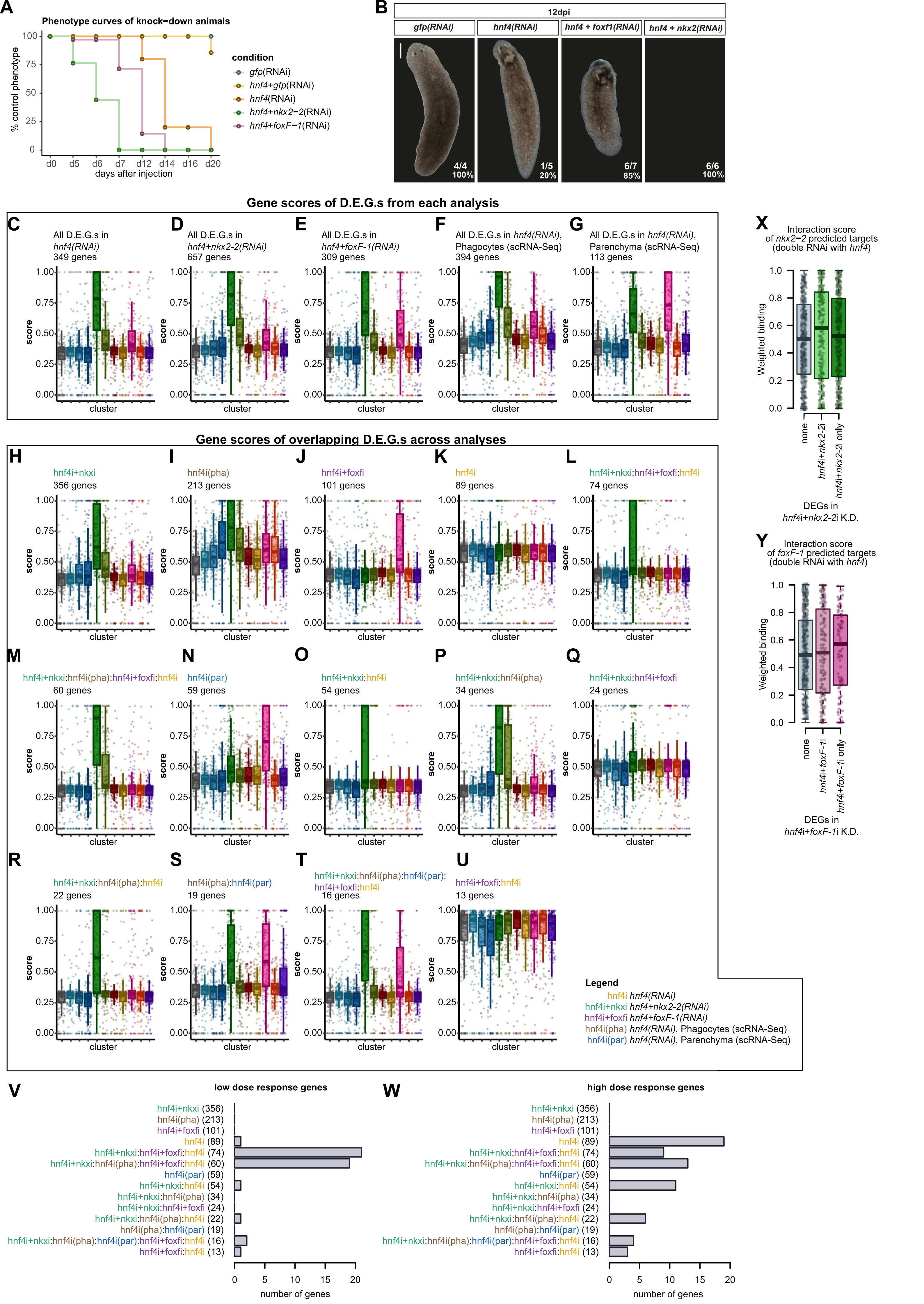
A
Phenotype curves of knock−down animals
% control phenotype
days after injection
d0 d5 d6 d7 d12 d14 d16 d20
condition
gfp(RNAi)
hnf4+gfp(RNAi)
hnf4(RNAi)
hnf4+nkx2−2(RNAi)
hnf4+foxF−1(RNAi)
B
12dpi
gfp(RNAi)
hnf4(RNAi)
hnf4 + foxf1(RNAi)
hnf4 + nkx2(RNAi)
4/4 100%
1/5 20%
6/7 85%
6/6 100%
Gene scores of D.E.G.s from each analysis
C All D.E.G.s in hnf4(RNAi) 349 genes
D All D.E.G.s in hnf4+nkx2-2(RNAi) 657 genes
E All D.E.G.s in hnf4+foxF-1(RNAi) 309 genes
F All D.E.G.s in hnf4(RNAi), Phagocytes (scRNA-Seq) 394 genes
G All D.E.G.s in hnf4(RNAi), Parenchyma (scRNA-Seq) 113 genes
score
cluster
X
Interaction score of nkx2−2 predicted targets (double RNAi with hnf4)
Weighted binding
none
hnf4i+nkx2-2i
hnf4i+nkx2-2i only
DEGs in hnf4i+nkx2-2i K.D.
Gene scores of overlapping D.E.G.s across analyses
H hnf4i+nkxi 356 genes
I hnf4i(pha) 213 genes
J hnf4i+foxfi 101 genes
K hnf4i 89 genes
L hnf4i+nkxi:hnf4i+foxfi:hnf4i 74 genes
M hnf4i+nkxi:hnf4i(pha):hnf4i+foxfi:hnf4i 60 genes
N hnf4i(par) 59 genes
O hnf4i+nkxi:hnf4i 54 genes
P hnf4i+nkxi:hnf4i(pha) 34 genes
Q hnf4i+nkxi:hnf4i+foxfi 24 genes
R hnf4i+nkxi:hnf4i(pha):hnf4i 22 genes
S hnf4i(pha):hnf4i(par) 19 genes
T hnf4i+nkxi:hnf4i(pha):hnf4i(par):hnf4i+foxfi:hnf4i 16 genes
U hnf4i+foxfi:hnf4i 13 genes
Y
Interaction score of foxF-1 predicted targets (double RNAi with hnf4)
Weighted binding
none
hnf4i+foxF-1i
hnf4i+foxF-1i only
DEGs in hnf4i+foxF-1i K.D.
Legend
hnf4i hnf4(RNAi)
hnf4i+nkxi hnf4+nkx2-2(RNAi)
hnf4i+foxfi hnf4+foxF-1(RNAi)
hnf4i(pha) hnf4(RNAi), Phagocytes (scRNA-Seq)
hnf4i(par) hnf4(RNAi), Parenchyma (scRNA-Seq)
V
low dose response genes
W
high dose response genes
hnf4i+nkxi (356)
hnf4i(pha) (213)
hnf4i+foxfi (101)
hnf4i (89)
hnf4i+nkxi:hnf4i+foxfi:hnf4i (74)
hnf4i+nkxi:hnf4i(pha):hnf4i+foxfi:hnf4i (60)
hnf4i(par) (59)
hnf4i+nkxi:hnf4i (54)
hnf4i+nkxi:hnf4i(pha) (34)
hnf4i+nkxi:hnf4i+foxfi (24)
hnf4i+nkxi:hnf4i(pha):hnf4i (22)
hnf4i(pha):hnf4i(par) (19)
hnf4i+nkxi:hnf4i(pha):hnf4i(par):hnf4i+foxfi:hnf4i (16)
hnf4i+foxfi:hnf4i (13)
number of genes

Supplementary Figure 23. A: Volcano plot showing differentially expressed genes. Dashed lines indicate fold change (vertical) and adjusted p-value (horizontal) thresholds for significance (Wald test). B: Heatmap showing expression of 'dose-dependent', differentially expressed genes across samples. Genes in rows, samples in columns. Genes have been grouped based on their dynamics as low dose dependent, mid dose dependent, and high dose dependent. C: Normalised gene score of low, mid, and high dose-dependent genes across all cell types in our single cell dataset. Centre line, median; box limits, upper and lower quartiles; whiskers, 1.5x interquartile range; points, subsample of data points for visualisation.

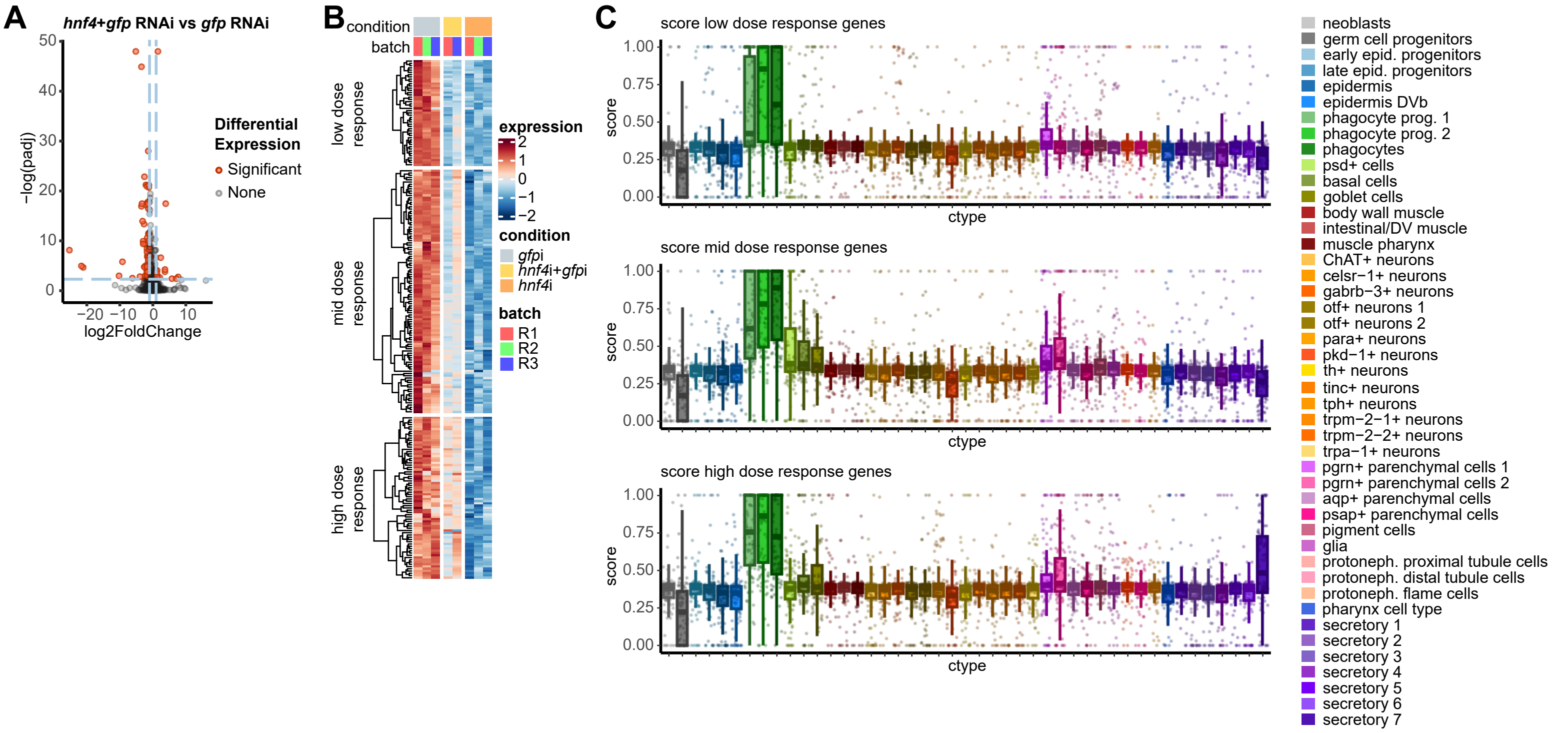
A
hnf4+gfp RNAi vs gfp RNAi
−log(padj)
log2FoldChange
Differential Expression
Significant
None
B
condition
batch
low dose response
mid dose response
high dose response
expression
condition
gfpi
hnf4i+gfpi
hnf4i
batch
R1
R2
R3
C
score low dose response genes
score mid dose response genes
score high dose response genes
score
ctype
neoblasts
germ cell progenitors
early epid. progenitors
late epid. progenitors
epidermis
epidermis DVb
phagocyte prog. 1
phagocyte prog. 2
phagocytes
psd+ cells
basal cells
goblet cells
body wall muscle
intestinal/DV muscle
muscle pharynx
ChAT+ neurons
celsr−1+ neurons
gabrb−3+ neurons
otf+ neurons 1
otf+ neurons 2
para+ neurons
pkd−1+ neurons
th+ neurons
tinc+ neurons
tph+ neurons
trpm−2−1+ neurons
trpm−2−2+ neurons
trpa−1+ neurons
pgrn+ parenchymal cells 1
pgrn+ parenchymal cells 2
aqp+ parenchymal cells
psap+ parenchymal cells
pigment cells
glia
protoneph. proximal tubule cells
protoneph. distal tubule cells
protoneph. flame cells
pharynx cell type
secretory 1
secretory 2
secretory 3
secretory 4
secretory 5
secretory 6
secretory 7

Supplementary Notes

Supplementary Note 1: On pseudo-bulk data and Weighted Gene Correlation Network Analysis (WGCNA)

Single cell analyses have generally focused on the one-versus-all approach for finding markers of cell types. We sought to investigate gene expression with a focus on the gene space. In other words, after having found groups of cells that express similar sets of genes with a one-versus-all approach (standard procedure with tools like Scanpy or Seurat), we sought to find groups of genes that express in similar ways across one or more of the cell types detected in our dataset. For this, we focused on the use of well-established tools in the field of bulk-sequencing such as Weighted Gene Correlation Network analysis (WGCNA) [1].

Briefly, WGCNA detects groups of genes with similar expression profiles across samples (where a sample can be anything from a tissue, experimental condition, or in our case, cell clusters), using:

(i) weighted correlation to detect connections between genes based on expression;

(ii) a topology overlap algorithm of the connections detected in step (i) to further refine connections between genes sharing many neighbours;

and (iii) detecting gene modules based on dynamic cutting of a clustering of genes in a tree of similarity using the data from step (ii).

WGCNA has been implemented in several platforms and for different kinds of data. In this study, we used the latest version of the R package `WGCNA` [1]. We refer to Zhang and Horvath [2] for a fully detailed explanation and application of the algorithm. Of note is that the original algorithm of WGCNA uses absolute values of correlations in the adjacency step, meaning it can potentially group genes with high positive correlation together with genes to which they correlate strongly but negatively. In practice, data derived from single cell methods tends to attract genes with high positive correlations only. This is because there are more cases of genes very highly expressed in one or few cell types than cases of genes with an exact inverse pattern, where a gene is generally expressed in all but one or few cell types.

We opted for a pseudobulk approach in order to compensate for the sparsity of the single cell data, as previously done in Álvarez-Campos *et al.* [3]. By aggregating counts of cells for each cluster, one can retrieve a matrix of 'genes x clusters' like those used in bulk sequencing data. In preliminary analyses, we normalised the pseudobulk data based on the size of each "library" (each pseudobulk cluster of cells) in a similar fashion to quantile normalisation, as implemented in packages such as DESeq2 [4]; this had the benefit of equating the dynamic range of variability between clusters, allowing for comparisons between clusters of small and large numbers of cells. However, we found that this normalisation had the disadvantage of inflating counts of spurious or lowly expressed genes in relation to the respective normalisation applied to that same gene(s) on large clusters. For example, a housekeeping gene found in three cells of a large cluster (thousands of cells) might have its expression dampened compared to their expression in two cells of a small (dozens or few hundreds) of cells. This led, in our experience, to genes with artificially enlarged expression values in small clusters, making them indistinguishable from (or at least, at comparable orders of magnitude to those of) truly cell type-specific genes. To our understanding, library size normalisation on pseudobulk data does not fully account for the bias of detection due to the low abundance of several cell types. We sought to investigate alternative ways to account for small clusters.

From a methodological standpoint, it is safe to assume that not every gene that is being expressed in a cell is truly indicative of the function and the dynamics of a given cell type. Firstly, there are a number of genes essential to the function of any cell that will be expressed at basal levels or when required, and this can be retrieved by the sampling. By this we mean both basal housekeeping genes (genes with very few counts per cell, in many cells of any cell type) and genes with occasional expression in any cell type (genes with moderate or high counts in few cells of any given cell type). Secondly, different sources of background signal and experimental artefacts must be considered, such as the likelihood of finding doublets, errors in the barcoding, or artefacts of the k-NN graph and clustering [5, 6]. We reasoned that the likelihood of capturing expression of a gene that is truly part of the functional machinery of a given cell type relative to the rest of the organism, is directly proportional to the fraction of cells of a given cell type (or in a cell cluster) expressing that gene relative to the rest of the organism. Thus, for a gene to be considered reliably expressed on a cell type, one must consider the dynamics of that gene in the whole dataset. One possibility is to take into account the fraction of cells expressing that gene in that cell type, in relation to the fraction of cells expressing that gene in the rest of the dataset. The relation between these two fractions can potentially be used as a dynamic weight of gene expression on each cell type, in order to better model a representative transcriptomic snapshot of each cell type.

For this approach, we computed another pseudobulk matrix, this time counting not the number of counts of each gene in each cluster, but the number of cells expressing each gene in each cluster. Then, for every gene *i* and every cluster *j* and using this matrix of number of cells, we calculated:

(i) The fraction of cells from cluster *j* expressing gene *i* (we called this number a_{ij}). This number is calculated by dividing the number of cells from cluster *j* expressing gene *i* divided by the number of cells in cluster *j*, and it can range between 0 (none of the cells in that cluster) and 1 (every cell in that cluster);

$$a_{ij} = \frac{n_{ij}}{N_j}$$

(ii) The fraction of cells not from cluster *j* expressing gene *i* (we called this number b_{ij}). This number is calculated by dividing the number of cells that do not belong to cluster *j* expressing gene *i* divided by the number of cells in the dataset that do not belong to cluster *j*, and it can range between 0 (none of the cells in the rest of the dataset) and 1 (every cell in the rest of the dataset);

$$b_{ij} = \frac{n_{i\bar{j}}}{N_{\bar{j}}}$$

(iii) The fraction between a_{ij} and b_{ij}, which we call C_{ij} . This number can be as low as 0 (no cells in cluster *j* expressing the gene compared to the rest of the dataset) but the range of values has no upper boundary (meaning a large fraction of cells in cluster *j* is expressing that gene compared to the rest of the data, which can be disproportionate if few or no cells are expressing that gene outside cluster *j*).

$$C_{ij} = \frac{a_{ij}}{b_{ij}}$$

(iv) This number, whose values can range from zero to infinite, is mapped to a range [0,1] using the formula `*1 - exp(- C*$_{ij}$*)*`, in order to be used as a weight. We call this number w_{ij}.

$$w_{ij} = 1 - e^{-C_{ij}} = 1 - e^{\frac{-a_{ij}}{b_{ij}}}$$

The resulting count matrix from normalising by library size can be adjusted using this matrix of weights. In practice, this dampens the spurious counts of lowly-expressed genes in small clusters.

We performed some preliminary comparisons between different normalisations, using the code present in the repository of this project. We ran WGCNA with each of them using broadly similar parameters and we observed that, compared to just library size normalisation or log(library size normalisation), using cell weights on top of log transformation of library size-normalisation performed better at detecting smaller modules for small cell clusters. With this method we did not observe large modules specific to small clusters that upon inspection corresponded to genes expressed broadly in the dataset, as it happened with normalisation by library size only. Therefore, we decided to adjust the library size normalisation by log transforming and using these weights for adjustment. Following WGCNA standard practices as well as previous works [7], we filtered out genes with less than thirty counts across the dataset (<30) and genes with low coefficient of variation too. As examples, lowly expressed genes expressed in infrequent cell types such as *pitx* (h1SMcG0012776) [8, 9] and *estrella* (h1SMcG0019080) [10] had 89 and 178 counts respectively.

WGCNA posits the idea that biological networks such as those of gene-gene interactions follow a scale-free model of graph topology [11], whereby the degree (number of connections per gene) distribution of the graph follows a power law. In other words, a scale-free network is expected to have many genes with few neighbours and few genes with many neighbours, these few genes acting as "hubs". Despite correlation alone (such as Pearson or Spearman) can coalesce genes in groups of similar expression profiles, weighing these correlation values using a soft threshold (or soft power) parameter is able to highlight strong correlations relative to weaker ones. This soft thresholding method can effectively yield a scale-free graph from correlation data, hence its use.

In order to detect the best-suited soft power, we ran the `pickSoftThreshold()` function from the WGCNA R package on our weighted, normalised data, and we chose soft power 8 as it showed a high scale free topology fit (based on Zhang and Horvath [2]) as well as a small median connectivity in order to detect smaller modules (Supplementary Figure 7B,C). After this, we ran the `adjacency()` and `TOMsimilarity()` functions to generate a topology overlap matrix (TOM), the opposite of which (1 - TOM matrix) can be used as a distance matrix for clustering the genes (Supplementary Figure 7D). We clustered the distance matrix derived from the TOM similarity matrix, and we finally ran the `cutreedynamic()` function of WGCNA. We set the parameter `deepSplit = 3` to retrieve small modules and set a minimum module size to 50 genes. We explored these modules by checking their overall expression dynamics (Supplementary File 5), their gene ontology enrichments (Supplementary File 7), and individual expression patterns on our scRNA original dataset (Supplementary File 6).

We sorted these modules in a semi-automated way by leveraging the dynamics of expression patterns of the genes constituting each module. We reasoned that modules could be mainly distributed in two groups: those which are highly specific for a given cell type (which we called "s" modules), and those whose genes are expressed in two or more cell types (which we called "m" modules). One way to tell these apart can be by checking in which cell type, or cell types, are their genes most often, and most highly, expressed. There are several ways to define what "highly expressed" means; for example, it can be measured as outlier values on the distribution of means, medians, or upper quartiles of expression on each cell type. These mean, median and upper quartile values can be derived from all of the genes of a module, or from those with intra-modular connectivity (==correlation with the average expression profile of the module) above a threshold value.

In this study, for every module *i*, we calculated the upper quartiles of the distributions of normalised counts from every gene of module *i* on every cell type. We defined outliers of this distribution of upper

quartiles as those values higher than the sum of the mean and 1.5 times the standard deviation of that distribution (Supplementary Figure 7E). Modules with only one outlier value of upper quartiles were defined as “s”; and those with two or more outlier upper quartiles were defined as “m”. After defining them as “s” or “m”, we reordered these modules following a similar pattern to the one chosen for listing the different cell clusters (i.e. neoblast first, epidermis second, etc, and then, neoblast and epidermis first, neoblast and phagocytes second, etc) (Supplementary File 4). Alternatively, there exist other methods such as the Tau metric as originally described in Yanai *et al* [12] and used more recently by Mantica *et al*. [13]. Indeed, we observed an agreement between the Tau metric and our WGCNA classification, as ‘sE’ genes had higher Tau values than ‘mE’ genes, whose Tau values were still higher than the rest of the genes (Supplementary Figure 7G).

To visualise these modules we used heatmaps using the ComplexHeatmap R package [14]. (Figure 2A, Supplementary Figure 8A) To facilitate the visualisation of the modules, we created profiles of the relative amount of gene expression of each module. For this, we first calculate the average expression profile of every gene from module *i* on every cell type, and then we then divide every value by the sum of values. This results in the frequency of expression of each module *i* on every cell type. The resulting frequencies represent the average expression signal in each cluster, and these were visualised as stacked bar plots on the side of the heatmap (Figure 2A).

Gene expression is driven, among other factors, by Transcription Factors (TFs). TFs bind to genomic regions where certain nucleotide sequences (their DNA motifs) can be found, such as promoters and enhancer regions. Therefore, if a group of genes shares a pattern of co-expression across certain cell types, there is a possibility that these genes form a module and are being co-regulated by the same TF (or groups of TFs). If that were the case, we would expect two conditions to be met:

(i) First, that TF must be expressed in those cells for that regulation to take place; thus, we may find one or more TFs whose profile of expression matches, at least to a certain degree, with the expression profile of the module. To evaluate this, we calculated the connectivity of every planarian TF with every module, by calculating the Pearson correlation between a given TF and the average expression profile of a given module (Supplementary Figure 8B).

(ii) Second, if that TF indeed regulates those genes, we may expect to find sequences of DNA that match the DNA motif of that TF in areas such as gene promoters. To evaluate this, we performed motif enrichment analyses for every set of gene promoters from each module, defined as 200bp up- and down-stream of the transcription start site (TSS) of every gene.

To evaluate the agreement between both, we calculated the correlation between a given TF connectivity profile and the motif enrichment profile of the motif corresponding to the TF of the same class and visualised the results on a heatmap (Supplementary Figure 8C). This allowed us to detect matching pairs of TF/motifs on certain modules, which we visualised as scatter plots (in Figure 2C, Supplementary Figure 8D). For example, for *hnf4*, we calculated the correlation of the motif enrichment of the nuclear receptor (NR) across a given set of modules, and the connectivity of *hnf4* across those very same modules; modules were chosen if (i) the motif enrichment is non-zero; (ii) the connectivity of *hnf4* to those modules is equal or larger than a threshold value (such as the upper quartile of connectivity values to all modules). These are the modules shown in Figure 2C for *hnf4* and the nuclear receptor motif. This allowed us to see that indeed we could detect certain TFs that express in certain cell types and whose expression matches with the enrichment of DNA binding motifs in the promoters of genes expressed in those same cell types,

suggesting a link between those gene modules and the TFs that might regulate them. We followed the same approach when inferring a link between certain TFs and the modules of co-accessibility in Figure 3, except this time we correlated the broad cell type-level expression of TFs against the average broad type-level accessibility profile of a given co-accessibility module, and the enrichment of motifs within OCRs of a given co-accessibility module (Figure 3C).

To further investigate these modules of co-expression, we generated a graph out of the TOM matrix using `igraph` [15], for different analyses and visualisation (Supplementary Figure 9A). We removed the spurious connections (connections between genes with values below the second-lowest value, below 0.01) and further explored these connections, observing the gene-gene connectivity value has an exponential decay-like pattern that can be bi or multimodal (Supplementary Figure 9B). For this dataset this value was around 0.3 and 0.4, which is slightly similar but not equal to our previous analyses [3]. We suggest exploring this for each dataset. Based on inspection of the distribution, we chose nine threshold values (Supplementary Figure 9C) over which we iterated to generate nine graphs and measure several metrics, such as the number of connected components, median degree, and median and number of genes per connected component (Supplementary Figure 9D-G). This was instrumental to ascertain the appropriate threshold value, as e.g. the graph with edge values above threshold 0.25 showed a much higher number of connected components (Supplementary Figure 9D), but these were formed by much fewer genes (Supplementary Figure 9G). We decided to focus on the threshold value 0.35, which preserved interactions between roughly 6300 genes (Supplementary Figure 9H) and observed that the vast majority of all the connected components correspond to unique modules of those detected by the dynamic tree cutting of WGCNA (Supplementary Figure 9I).

Alternatively, since every gene in the graph belongs to a WGCNA module as computed earlier, one can isolate the different WGCNA modules from the graph which can be useful for intra-modular analyses, but this precludes further cross-module analyses as any connection between modules is lost. We followed this approach to study the relationship between TF connectivity and centrality. As suggested in our previous publication [3] we noticed TF intramodular connectivity is in agreement with TF centrality, both globally (when correlating the relative intramodular connectivity and the relative centrality of TFs from all modules) and module-wise (Supplementary Figure 9J). Therefore, this measure of connectivity and centrality can help identify potentially relevant TFs for regulating the expression of genes in a given module.

We then wondered what the structure of this network at the module level was like. Are genes from one module more connected to other modules? If so, are they functionally similar? Are they similar at the transcriptional regulatory landscape (TF and motifs) too? To answer these questions:

(i) We first generated a cross-connection graph as described previously [3], by counting how often a gene of a given module *i* is connected to genes from other modules. We normalised this number by module size, as large modules likely tend to have more cross-connections. We used this 'module x module' matrix to generate a module-wise graph of cross-connections (Supplementary Figure 9K).

(ii) Secondly, we used the motif enrichment analyses we did previously (see Methods) to calculate a motif x module matrix of percentage of enrichment in gene promoters, in turn used to create a module-wise graph of motif enrichment similarity (Supplementary Figure 9L).

(iii) Thirdly, for further exploration we did module connectivity of the TFs against the eigengene of every module (average profile of a module as done in WGCNA). We retrieved the profile of TF

connectivity for each module and correlated this to make another module-wise graph (Supplementary Figure 9M).

(iv) Next, we did a COG functional category enrichment analysis as described in Álvarez-Campos *et al.* [3] and we used the percentages of over- or under-representation to generate a functional category x module matrix, to create a module-wise graph for functional category enrichment (Supplementary Figure 9N).

(v) We finally aggregated these graphs together counting how often a pair of modules appears connected in each (Supplementary Figure 9O).

Using the aggregated module-wise graph from step (v), we detected communities of modules using the `cluster_label_prop()` function from igraph, which overall aligned with our previous observations and provided a broad view of the dynamics at the gene space level in *S. mediterranea* at the co-expression, functional, and potential regulatory level.

A full graph of all the genes can be also visualised in Supplementary Figure 9P.

Of note, we also followed a similar rationale when exploring the sets of co-accessible regions of open chromatin (Figure 3). We ran WGCNA and chose a soft threshold power, and we later sorted the modules of co-accessible regions in a similar fashion. Because our module sorting approach is actually agnostic from the source of the data (as long as these are groups of features such as genes or OCRs), we also followed this approach to group and sort the clusters of co-influential TFs.

To evaluate whether there is a broad agreement between the co-expression and co-accessibility modules, we pooled genes and OCRs in gene-OCR pairs based on relative genomic distance (in bp), by assigning every OCR to the closest nearby gene. Because genes are assigned to co-expression modules and OCRs are assigned to co-accessibility modules, this effectively allows us to categorise OCR-gene pairs in two ways.

We can, first and foremost, quantify how many OCR-gene pairs belong to every possible pair of co-expression and co-accessibility modules. This is done in Supplementary Figure 10F. By visualising every module with the colour of the cell type where these features (genes, OCRs) have maximal expression or co-accessibility, we see that genes from co-expression modules of a given cell type are found near OCRs belonging to co-accessibility modules of that very same cell type (at the broad level).

Secondly, we can evaluate whether these values are higher than expected. For that we performed an upper-tail hypergeometric test to compare these observed values against the expected values (that is, the cumulative probability of finding an OCR/gene pair from a given module of co-expression X and a given module of co-accessibility Y). These results can be observed in Supplementary Figure 10G.

Overall, this suggests that co-expression dynamics recapitulate co-accessibility dynamics.

Supplementary Note 2: Weighted correlation analysis on differentially accessible OCRs

Our weighted correlation network analysis of OCRs in planaria revealed patterns of co-accessibility that hint at some cell types sharing certain aspects of their regulatory logic. However, this scATAC-seq data is noticeably sparser than our scRNA-seq data, and we decided to perform a more conservative version of this analysis. We aimed at selecting a more rigorous subset of OCRs to perform WGCNA analysis. For this, we decided to perform differential accessibility analysis to select OCRs that appeared significantly accessible in one cell type in relation to others. Since our scATAC-seq data does not have replicates, we generated pseudoreplicates following the rationale from Hafemeister & Halbritter, 2023[16]. To this end, we split our sc-ATACseq data in two pseudoreplicates to perform differential chromatin accessibility analysis for each cell type (Supplementary File 11A, Supplementary Files 9, 10).

We first compared every cell type against the rest of cell types in order to identify cell type-specific open chromatin regions (OCRs) (Wald Test, two-sided, multiple hypothesis correction). Our method reliably detected differentially accessible OCRs for every broad cell type except for neoblasts (Supplementary File 11B). Moreover, marker analysis also revealed regions and motifs specific to each broad type, except for neoblast, which raised regions generally open in all cell types, as if they were constitutive regions (Supplementary Figure 4B). To further corroborate these points, we selected markers of neoblasts using the scRNA-seq dataset and observed their activity in the scATAC dataset. This analysis revealed that their signal was not neoblast-specific either (Supplementary Figure 4C). The OCRs derived by our differential chromatin accessibility analysis are distributed in chromatin accessibility peaks (Supplementary File 11C). These findings align with previous observations that neoblasts lack a specific chromatin signature [17].

In addition to this one-versus-all approach, we also compared chromatin accessibility of every cell type against neoblasts in order to retrieve differentiated cell type OCRs (Wald Test, two-sided, multiple hypothesis correction) (Supplementary File 11D,E). This allowed us to retrieve a second set of differentially accessible OCRs for every major cell type which partially agreed with our cell type-specific chromatin regions (Supplementary File 11F, Supplementary Files 9, 10), suggesting our second approach was detecting a different set of OCRs that may be accessible in other cell types and which we also observed by visual inspection of some of these OCRs (Supplementary File 11G).

We further explored this by performing a second WGCNA analysis using these differentiated cell type OCRs (those from our second D.C.A. analysis, Supplementary File 11D,E) as input, which successfully clustered the OCRs into modules of co-accessible regions in different cell types (Supplementary File 11H-J, Supplementary File 14). These modules contained OCRs predominantly open in one cell type, such as muscle (modules s01 and s02) or neuronal cells (modules s04 and s05), but also modules of accessibility specific to multiple cell types. For example, we detected three modules of OCRs accessible in parenchymal and gut cells (s06, m05, m08), and also modules of OCRs open in muscle, neuron, and secretory cells (m02, m03, m09, m10). These combinations of cell types match the profiles of genes with expression specific to multiple cell types we observed in previous analyses (Figure 2A), and these modules overall also correspond to the less strict analysis carried out in Figure 3. To ascertain if these patterns of accessibility specific to multiple cell types translated into expression in these multiple cell types, we associated each OCR to their closest gene model and analysed their gene expression patterns. We visualised as a heatmap the genes with highest correlation between accessibility and expression (Supplementary File 11K-M), which showed

high agreement. This analysis shows that many genes associated with these OCRs were also expressed in the same cell types.

Supplementary Note 3: details on ANANSE

Despite gene expression analysis and chromatin analysis providing valuable insights, these analyses are run separately from each other. For an integrated analysis of the two data modalities, we used ANANSE [18]. ANANSE is a tool that leverages TF/target gene annotation, gene expression, chromatin accessibility, and motif enrichment analysis to assign a probability to TF-target gene interactions based on a score from an additive model. If available, ANANSE can also leverage histone modification data such as H3K4me3 or H3K27Ac ChIP-Seq, although it is not strictly needed for network inference. Since we have transcriptomics and chromatin accessibility data, we can run ANANSE with our data. The resulting output is a list of TF-target gene interactions defining a graph, or network, of TF-target gene predicted interactions.

Because we can computationally dissect our single cell data to isolate signals coming from each cell type, it is feasible to construct such networks of TF/target gene interactions for every *S. mediterranea* broad cell type. In addition, ANANSE incorporates a feature (ANANSE influence) to compare pairs of networks using differential gene expression analysis, in order to e.g. identify the potential TFs driving the transition from one given cell state to another. This approach proves instrumental to study these networks from the perspective of pluripotent stem cells committing to different cell fates, such as is the case of *S. mediterranea* neoblasts. Therefore, we sought to investigate if our data is capable of recapitulating known dynamics related to cell type differentiation in *S. mediterranea*.

To create these networks, it becomes necessary to consider which genes in the dataset are TFs, and which motifs correspond to which TFs. ANANSE needs to tell TFs apart from non-TF genes, which can be achieved by providing a database of TF-motif association. There are several ways to do this, and we implemented a two-step solution:

(i) Firstly, the motif database of choice is relevant. ANANSE relies on gimmemotifs, a suite for motif enrichment analysis [19]. gimmemotifs incorporates motif databases from a number of projects, notably HOMER [20] and JASPAR [21]. The latter database provides, separately, a tool to infer the predicted motif of a TF based on sequence homology and DNA-binding protein domain prediction [22]. Therefore, choosing the JASPAR database has the advantage that this information can be provided with a tool specific for that database.

(ii) Secondly, since gimmemotifs was designed for human and mouse data, it also has a tool called `motif2factors` that relies on automated orthology inference to identify TFs based on sequence homology, using Orthofinder [23] to transfer TF (and motif) annotation from human and mouse to the species of interest.

As explained in Methods, we ran `motif2factors` to transfer motif annotation from the JASPAR database, using a set of model organisms complemented by several protostome metazoan species (Supplementary File 15, Supplementary Figure 12A) to provide additional phylogenetic signal for the automated transfer. Second to this, we observed that not every TF detected by our TF annotation workflow (see Methods) received a motif. For this we ran the JASPAR similarity prediction tool [22] on those TFs that did not get any transferred motif, using the JASPAR 2024 motif database which overlaps with the JASPAR 2020 database.

Finally, to ensure the vast majority of TFs from the literature had an annotated motif for ANANSE, we supplied this motif annotation with any remaining TF that had an associated motif as detected by Neiro *et al*., 2022 [24].

The resulting database of 401 (out of 665) TFs was adapted to a format compliant to gimmemotifs and subsequently used for running ANANSE.

Next, we took our database of OCRs (the output peak file from cellranger) and re-centred the coordinates of the OCRs around the summit of the ATAC-seq signal, for optimal results with gimmemotifs.

In parallel to these steps, the input data for ANANSE must be prepared. Briefly, ANANSE runs on two steps:

(i) the "binding" step where a TF x region matrix of predicted binding activity is computed using the TF/motif annotation and the BAM file, effectively generating a profile of TF binding activity on every region of open chromatin in the dataset;

(ii) the "network" step where the binding information generated in step 'i' is leveraged with gene expression data, to generate the TF-target gene predicted interactions.

To run these steps for each cell type, we computationally dissected the scRNA-seq and the scATAC-seq data to separate the signal from each cell type (Figure 4A). Since the lowest resolution of the data comes from the scATAC-seq, we used the eleven broad cell types as defined by the scATAC-seq data. These are neoblasts, early epidermal progenitors (also referred in the manuscript and figures as "early epid. prog.", or "eep"), late epidermal progenitors (also referred in the manuscript and figures as "late epid. prog.", or "lep"), epidermis, phagocytes, basal and goblet cells (together as a single group), muscle, neurons, parenchyma, protonephridia, and secretory.

(i) First, as explained in Methods, we split the scATAC-seq BAM file generated by `cellranger-atac` [25] using the software `sinto` (https://github.com/timoast/sinto) and the list of cell barcodes belonging to each of the eleven broad types (which we know because of our scATAC-seq analysis and the because of the alignment of the scATAC-seq and the scRNA-seq data) to generate independent BAM files for each of the eleven broad types. These BAM files were later used as input for ANANSE binding.

(ii) Second, we performed a pseudobulk computational dissection on the scRNA-seq dataset at the broad type level, to generate eleven tables of counts of RNA, one per broad cell type. These tables of counts were later used as input for ANANSE network.

We then proceeded to run ANANSE binding for each of the eleven broad types, as explained in Methods. The resulting binding profiles were used alongside the counts tables to generate eleven networks of interactions.

For a preliminary assessment of these networks, we ported these networks to R and igraph. Since ANANSE's workflow scores every putative TF-target gene interaction based on a rank of probability, one must prune the network from gene-gene interactions based on a threshold. For every network, we did a pre-processing by keeping TF-target gene interactions with score values above 0.8, and any gene without neighbours (degree == 0) was removed.

Next, as explained in Methods, we calculated centrality, out-centrality, in-degree, and out-degree for every gene on every network. Relative out-degree was calculated as in-degree divided by the sum of

in-degree and out-degree. We calculated the number of active TFs as the number of genes with outdegree above 0.

Then, for every network, we extracted the centrality values for all the TF genes in the network. We did so by running the function `closeness()` from igraph [15] with `method = "out"`. Lastly, we collapsed these values together in a TF x cell type network matrix of centrality values (Supplementary Figure 12C,D). To assess the degree of similarity of these cell types based on the profile of TF centrality, we clustered the columns of this matrix using the following parameters: `method = "ward.D2"` (Supplementary Figure 12D,E). With this we observed, as explained in Results, that the epidermis networks group together which provides support to our analyses. Interestingly, we also observed that gut and parenchyma networks group together, which aligns with some of our previous analyses. Overall, we interpreted these observations as confirming the validity of the methodology.

With this, we proceeded to run ANANSE influence, the third step. ANANSE influence takes as input two networks, A and B, and a list of differentially expressed genes between conditions A and B. By default, ANANSE network focuses on up-regulation from a condition to the other, which leaves out genes downregulated in B relative to A. To generate the list of differentially expressed genes (DEGs), we ran our pseudobulk DGE analysis comparing each of the ten non-neoblast broad types against neoblast (Supplementary Figure 13A), as explained in Methods. In particular, we excluded late epidermal progenitors as this is an intermediate state but decided to keep early epidermal progenitors to have a comparison between neoblasts and an immediately consecutive state. Thus, we retrieved nine lists of DEGs, one for each transition from neoblast to a non-pluripotent cell type. We called these transitions "fates" in the manuscript.

ANANSE influence allows to compare two networks, one from a source cell type (neoblasts, in our case) and another from a target cell type (for example, phagocytes). ANANSE calculates a score of influence for each TF in the network of the target cell type, that ranges from 0 to 1 by leveraging differential gene expression between the source and the target cell type. The higher the score, the more likely the network structure of the target cell type is due to the contribution of that TF to the network. For each fate, we provided ANANSE influence with the neoblast network, the relevant non-neoblast network, and the list of DEGs in the comparison of the relevant non-neoblast broad type against neoblasts. ANANSE influence focuses on a top number of interactions based on the probability score, which we set to top 250,000, and computes an influence score based on the differential gene expression analysis. The resulting output comprises a list of top influential TFs (visualised in Supplementary Figure 13B-J, Supplementary File 16) as well as a "differential network" –containing the influential TF-target gene interactions detected only in the differentiated cell type. For example, the "differential network" of phagocytes is the network made of TF/TG interactions from the phagocytes network that are only found in the phagocyte network and not in the neoblasts network.

We ported these differential networks to R and generated graphs out of them. For the sake of visualisation only, we pruned these networks and kept the top two interactions per TF (Supplementary Figure 14). In this same figure, we coloured the TFs based on the relative outdegree in their differential network. Relative outdegree means number of emitting connections compared to other TFs in that network. The brighter the colour intensity of a TF, the more connections it is emitting compared to other TFs in that network. For example, *soxP-3* from Figure 4C is quite blue because *soxP-3* is emitting a lot of connections to other genes in the differential network of early epidermal progenitors. We then crossed our differential network data with gene annotation from the planarian literature (using PlanMine [26] to label target genes, and visualised them using ggplot2 (Supplementary Figure 14, to the right of the network plots) (Supplementary Files 15, 16, 17). In these jitter plots, each data point

is a gene, and the value shown in the Y axis is the interaction score with the chosen TF. Some of these, which appear outlined, are known, well-described genes from the planarian literature. For example, we find *gcr052* and *ferm1* among the top targets of *soxb1-2* in the neoblast-to-epidermis cell fate network (Supplementary Figure 14B).

We also explored the structure of the networks from a functional perspective. We wondered if ANANSE was able to successfully predict the interaction between TFs and bona-fide predicted target genes. For this, we took a subset of TFs for which there is knock-down data in the literature [27-30] and checked their presence in the different cell fate networks. All *pax2/5/8-1*, *p53*, and *soxP-3*, were found influential throughout the epidermal lineage, and *coe* was found influential in the neuron network. To visualise and glimpse the subgraph centred around such TFs, we subsetted a given graph for the TF of interest and kept: (i) any direct neighbour that receive top highest interactions from our TF of interest, together with (ii) any interaction between those direct neighbours (Figures 4C-F). We then re-analysed the RNA-Seq data publicly available for these knockdowns, and we found that these genes tended to express in the same cell types where these TFs are influential (Supplementary Figure 15, Supplementary Figure 16, Supplementary Note 4, Supplementary Files 20, 21, 22, 23). Importantly, we queried the interaction and weighted binding scores between each TF and the down-regulated genes (Figure 4A), and we found a general trend for agreement -downregulated genes indeed have higher interaction and weighted binding scores than non-downregulated genes (Figures 4C-F, box plots). We also analysed the RNA-Seq knock-down data from *prep* (a TF expressed in the anterior pole of the planaria) and found a similar trend, with high scores and weighted binding in the networks of cell types where prep is highly influential (Supplementary Figure 17, Supplementary Files 22, 24). In addition, we found that the top predicted targets of prep also had high expression in the anterior pole of the animal. Overall, these analyses orthogonally validate the predictive power of ANANSE. We also found similar trends for other TFs such as *alx3-1*, as well as the TFs for which we performed single and double knockdowns (*hnf4*, *nkx2-2*, *foxF-1*) (Figure 6F, Figure 8K-L, Supplementary Figure 18, Supplementary Figure 22, Supplementary Files 26, 32).

We observed that several TFs appeared as influential in more than one cell type (Supplementary Figure 14, Supplementary File 18). To assess the degree of co-influence of each TF, we collapsed the influence scores of each TF on each fate, generating a TF x fate matrix of influence scores. We subjected this matrix to hierarchical clustering and cut the clustering tree very granularly to detect small groups of co-influential TFs (Supplementary Figure 13K). For each of these clusters, we applied sorting following the same approach as previously described, we computed the average influence profile across fates and correlated every TF of the same cluster against that average influence profile (Supplementary Figure 13L,M). We selected the top five highly correlating genes per cluster as the top co-influential per group (Figure 4B).

In addition to the probability score, ANANSE also reports the individual parameters estimated for each TF-target interaction, of which weighted binding is particularly interesting. In Figure 7, we show that differentially expressed genes of the *hnf4*i knock-down have higher weighted binding of the *hnf4* TF than non-DEGs (Figure 7E). We further evaluated the predictive power of ANANSE by modelling a logistic regression between the ANANSE interaction scores and the probability of successfully detecting a down-regulated gene (Supplementary Figure 21H).

Overall, these analyses show that our data recapitulates known dynamics related to cell type differentiation in *S. mediterranea*. Supported by further validation (Figure 6, Figure 7, see Results), we showed that analyses have the potential of generating effective hypotheses about interactions driving cell fate specification in *S. mediterranea*.

Caveats of this method:

The resolution of the scATAC limited our GRN reconstructions, as we could observe some TF/target scores were primarily driven by gene expression similarity and not by chromatin-related metrics such as weighted binding. Other sources of epigenetic information that contribute to regulatory logic, and that are not currently considered, include DNA methylation and epigenetic marks. Additional data modalities, such as chromatin immunoprecipitation and sequencing (ChIP-Seq) of histone marks for each broad cell type, will prove useful as ANANSE can incorporate them to the GRN modelling. These marks can also help better define the role of an OCR as a promoter, an enhancer, or a silencer, which can benefit the accuracy of the reconstruction. In addition, despite a high agreement between gene expression and chromatin accessibility as shown in our and similar works, and despite being the basis of methods such as RNA/ATAC integration[31, 32], chromatin state does not always equate to gene expression. There are temporal dynamics at play as chromatin needs to be made accessible prior to genes being expressed, and this latency might affect GRN reconstruction. Likewise, our approach relied on positive correlation, when it is known that some of this cis-regulatory elements might act as repressors. Finally, future GRN inferences could benefit from experimentally validated association of TFs and motifs, as our approach relied in electronic transference of motifs annotated in other species (which we tried to account for by adding a number of protostome species to increase the phylogenetic signal). Alternatively, *de novo* motif finding across the genome (helped by foot printing algorithms), followed by clustering-based annotation of the motifs, can likely improve the candidate motifs that could greatly enhance the detection. Despite these limitations, our joint approach of computational GRN inference and experimental validation illustrate that GRN reconstruction, thanks to these approaches, can be widely applied across multiple species and biological contexts.

Supplementary Note 4: Re-analysis of planarian data from the public literature

Anteroposterior transcriptomics dataset:

We mapped the anteroposterior data from Stückemann *et al.*, 2017 [33] against the latest version of the *S. mediterranea* genome. We used bowtie2 with parameters `-p 20 -X 75`. The resulting bam was ran through HTSeq with standard parameters `--nonunique all -f bam -r pos –type gene -s no -n 12` and the GTF annotation of the latest version of the *S. mediterranea* genome.

The resulting raw counts were loaded onto R and subjected to fpkm normalisation from the `countToFPKM` R package, using the gene body lengths from the latest annotation of the *S. mediterranea* genome as normalising factor, and afterwards we performed quantile normalisation to stabilise the range of expression. We visualised the majority of genes shown in Figure 2 of the original manuscript as a means to check the proper data retrieval and normalisation.

The resulting fpkms were processed as described in Stückemann *et al.* [33]. Briefly, we implemented the three filters from the original manuscript and we keep a gene if the following applies: (i) the gene expression profile shows at least three non-zero consecutive data points across the samples, (i) the ratio between the maximum and the minimum values of expression across all samples is equal to 2 or higher, and (iii) a ratio between the standard deviation about the smoothed curve (Savitzky-Golay filter from the `signal` R package) smaller than 8x the difference between minimum and maximum values of the original data. In addition, we applied a coefficient of variation filter to remove genes with low variance across the anteroposterior axis. This left us with 9078 genes for gene clustering.

For clustering, we used the R package `mfuzz`, with an m-estimate of `m = 1.26` (from the ` mestimate()` function of `mfuzz`). We estimated the number of clusters using the `Dmin()` function from `mfuzz`, and we observed the elbow of the curve lay between 15 and 20 clusters. After assigning all genes to any of these 20 clusters, we subsetted for high-affinity genes by keeping genes with an alpha-core value against its assigned cluster of 0.45 or higher (using the ` acore()` function). This left us with 4836 genes.

The resulting 20 clusters were re-arranged based on the maximum of average expression of all genes belonging to the cluster. Expression was visualised using the `ComplexHeatmap` R package, and data was saved for later use in other downstream analyses (Supplementary File 22, Supplementary Figures 16, 17).

Data from *soxP-3*, *p53*, and *pax2/5/8-1* knockdowns (Cheng *et al.*, 2018, Tu *et al.*, 2015):

We mapped the knockdown data from Cheng *et al.*, 2018 [27] and Tu *et al.*, 2015 [28] against the latest version of the *S. mediterranea* genome. We used bowtie2 with parameters `-p 20 -X 75`. The resulting bam was ran through HTSeq with standard parameters `--nonunique all -f bam -r pos –type gene -s no -n 12` and the GTF annotation of the latest version of the *S. mediterranea* genome. We filtered genes with less than ten counts in ten samples.

The resulting raw counts were loaded onto R and ported over to DESeq2 [4]. We performed Wald test, two-sided, with the matrix design formula ` ~ replicate + time + RNAi + RNAi:time`. To retrieve differentially expressed genes for each gene, we evaluated the p.adjusted (multiple comparisons correction) and log2foldchange of every comparison involving the variables `time` and `RNAi`, when the variable `RNAi` corresponded for the TF of interest. Genes were considered differentially expressed if the p.adjusted (Wald test) was less than 0.05 and the absolute log2Foldchange was greater than 1.5.

We visualised expression of differentially regulated genes using the `ComplexHeatmap` R package. For each set of differentially expressed genes, we checked the localised expression at the cellular level by calculating a gene score (see Methods) for these genes in all the cells of our single cell dataset. We noticed that cells from the epidermal lineage had higher scores for the differentially expressed genes in the knockdowns of all three TFs. Leveraging these genes with our network data revealed that the down-regulated genes in the knockdowns of these TFs had higher ANANSE interaction scores and weighted bindings than genes not detected as differentially expressed.

Data from *coe* knockdown (Cowles *et al*., 2014):

We mapped the knockdown data from Cowles *et al*., 2014 [34] against the latest version of the *S. mediterranea* genome. We used bowtie2 with parameters `-p 20 -X 75`. The resulting bam was ran through HTSeq with standard parameters `--nonunique all -f bam -r pos –type gene -s no -n 12` and the GTF annotation of the latest version of the *S. mediterranea* genome. We filtered genes with less than ten counts in ten samples.

The resulting raw counts were loaded onto R and ported over to DESeq2 [4]. We performed Wald test (two-sided) with the matrix design formula ` ~ batch + condition`. To retrieve differentially expressed genes for each gene, we evaluated the p.adjusted (multiple comparisons correction) and log2foldchange of the contrast `c("RNAi", "coe*(RNAi)*", "control")`. Genes were considered differentially expressed if the p.adjusted (Wald test) was less than 0.05 and the absolute log2Foldchange was greater than 1.5.

We visualised expression of differentially regulated genes using the `ComplexHeatmap` R package. We checked the localised expression at the cellular level by calculating a gene score (see Methods) for these genes in all the cells of our single cell dataset. We noticed that epidermal cells and certain neuronal cell types had higher scores. When leveraging these genes with our network data, we observed that the down-regulated genes in the *coe* knockdown had higher ANANSE interaction scores and weighted bindings than genes not detected as differentially expressed. The only exception was the weighted binding between *coe* and downregulated target genes in the neuronal network, which was no different from that of non-downregulated genes. We think this may be due to the low resolution of our scATAC-seq data, which is not enough to detect the signal of coe motif enrichment coming from the neuronal subpopulations where coe is expressed.

Data from *prep* knockdown (Kao *et al*., 2013):

We mapped the prep knock-down data from Kao *et al*., 2013 [30] against the latest version of the *S. mediterranea* genome. We used bowtie2 with parameters `-p 20 -X 75`. The resulting bam was ran through HTSeq with standard parameters `--nonunique all -f bam -r pos –type gene -s no -n 12` and the GTF annotation of the latest version of the *S. mediterranea* genome.

The resulting raw counts were loaded onto R and subjected to fpkm normalisation from the `countToFPKM` R package, using the gene body lengths from the latest annotation of the *S. mediterranea* genome as normalising factor. We then filtered out genes with less than 50 counts in either of the conditions, and afterwards we performed quantile and log- normalisation to stabilise the range of expression.

The original dataset does not have replicates. To detect "misregulated" genes, we proceeded as previously described in the original manuscript [30]. Briefly, we calculated the Fold Change of the *prep(RNAi)* gene expression data over the *gfp(RNAi)* gene expression data and defined "misregulated genes" as genes with a foldchange above 2 (for up-regulation) or below 0.5 (for down-regulation).

We checked the localised expression of these misregulated genes by visualising their expression pattern in the antero-posterior dataset [33] using the `ComplexHeatmap` R package, and we noticed that the majority of “down-regulated” genes were expressed in the anterior part of the animal. In addition, we also checked the localised expression of these genes at the cellular level by calculating a gene score (see Methods) for these genes in all the cells of our single cell dataset. We observed that epidermis, neurons, parenchyma and protonephridia (which also includes pharynx cells) had higher gene scores, which was indicative of the cell types where prep is expressed. We also observed that prep top targets (predicted by ANANSE) tend to express in the anterior part of the planaria.

Data from *alx3-1* knockdown (Akheralie *et al.*, 2023):

We mapped the knockdown and control data (intact, not wounded nor regenerating worms) from Akheralie *et al.*, 2023 [35] against the latest version of the *S. mediterranea* genome. We used salmon [36] with parameters ``. The resulting raw counts were loaded onto R and ported over to DESeq2 [4]. We performed Wald test (two-sided) with the matrix design formula `  ~ batch + condition`. To retrieve differentially expressed genes for each gene, we evaluated the p.adjusted (multiple comparison correction) and log2foldchange of the contrast `c(“RNAi”, ”alx3-1*(RNAi)*”, “control”)`. Genes were considered differentially expressed if the p.adjusted (Wald test) was less than 0.05 and the absolute log2Foldchange was greater than 1.5.

We visualised expression of differentially regulated genes using the `ComplexHeatmap` R package. We checked the localised expression at the cellular level by calculating a gene score (see Methods) for these genes in all the cells of our single cell dataset. We saw that neurons and secretory cells had higher scores, although not muscle cells. To leverage DEGs against our network data, for each cell type network, we marked genes as downregulated by *alx3-1* if they were detected as down-regulated in the DGE analysis and if their max value of gene expression (normalised pseudobulk, see Methods) was in any of the cell types of the same broad cell type as the network (e.g. a gene downregulated in the *alx3-1* knockdown with max expression in muscle was not queried for the neuronal network, but it was queried for the muscle network). Leveraging these genes with our network data revealed that down-regulated genes in the *alx3-1* knockdown had higher ANANSE interaction scores and weighted bindings in the networks of muscle, neuron, and secretory cells, relative to non-DEGs.

Target gene knockdown

Throughout our reanalyses, we found the transcription factor being targeted by RNAi among the DEG list in some cases (*alx3-1*, *coe*, and *p53*), but not always. In fact, *soxP-3*, *pax2/5/8-1*, and *prep* not significantly downregulated after their own knockdown treatment. This is an observation that is likely underreported in some publications, but our systematic reanalyses show that this expectation is not always met. Several lines of argumentation can potentially explain this observation. First, TFs are genes that regulate many other genes and are typically expressed at low levels. It is possible that relatively small, undetectable, changes in their levels cascade into much larger effects for their target genes. Second, it is unclear how long the products of the RNA targeting by the RNAi machinery last. It is possible that cleaved mRNAs persist, unable to render proteins, but still detectable by RNA sequencing approaches. Third, it is possible that the rate of knockdown varies across cell types, and with cell types themselves changing their proportions, this can lead to a complex dynamic. Fourth, it is unclear if the dsRNA itself can be detected as cDNA in an RNAi experiment. Finally, it is plausible that feedback loops upregulate the gene under knockdown treatment. It is also possible that this elevates the levels of pre-mRNA detectable in the nucleus, but since the RNAi machinery is thought to be cytoplasmic, these new copies are still degraded before being translated. Altogether, our results

show that this question deserves further attention and will contribute to the careful examination of mRNA levels after knockdown in future publications.

Supplementary Note 5: Notes on the double knock-down experiments

1. hnf4i+gfpi and dose dependence

As part of our double knockdown analyses, we considered the potential impact of combining more than one gene knockdown via dsRNA injection. We argued that mixing of two dsRNAs, while potentially driving a combined effect, may drive down the penetrance of each individual dsRNA treatment. Therefore, we decided to evaluate the effect of dsRNAi dosage for a single of our genes of interest, *hnf4*. For this, we treated animals either with only *gfp* dsRNA (*gfp(RNAi)*), with only hnf4 dsRNA (*hnf4(RNAi)*), or with a 1:1 combination of both (*gfp(RNAi)+hnf4(RNAi)*). The amount of dsRNA in each case was standardised, as we injected the same volume. Survival of *gfp(RNAi)+hnf4(RNAi)* animals was the same as control animals, as shown in Figure 8C, and the global transcriptomic profile of *gfp(RNAi)+hnf4(RNAi)* knockdown animals was similar to that of *hnf4(RNAi)* animals (Figure 8D), although one sample deviated from the general trend. We decided to discard this sample for downstream analyses. In a similar manner to that of the hnf4 knockdown analysed in Figure 8, we compared gene expression between *gfp(RNAi)+hnf4(RNAi)* and *gfp(RNAi)* animals. Differential gene expression analysis (Wald Test, two-sided, multiple comparison correction) revealed an overall pattern of down-regulation (Supplementary Figure 23A), aligning with the pattern of *hnf4(RNAi)* and other TF knockdowns.

To further inspect these differentially expressed genes, we pooled genes downregulated in either condition (*hnf4(RNAi)*, and *gfp(RNAi)+hnf4(RNAi)*) and clustered their expression profile across samples. Hierarchical clustering of average-replicated gene expression profiles unveiled three clusters of D.E.G.s that largely match a potential dose-dependent dynamic (Supplementary Figure 23B). One of the clusters, which we called low-dose-dependent genes, corresponded with genes whose expression was downregulated both in *gfp(RNAi)+hnf4(RNAi)* animals and in *hnf4(RNAi)* animals, suggesting regulation of these genes is more sensitive to smaller changes in the expression of *hnf4*. Another cluster, which we called mid-dose dependent, was comprised of genes downregulated in *hnf4(RNAi)* animals and had lower expression levels in *gfp(RNAi)+hnf4(RNAi)* animals than in *gfp(RNAi)* animals and included genes retrieved as down-regulated genes in either or both conditions. Lastly, we termed another cluster as high-dose-dependent genes, as these genes were only down-regulated in *hnf4(RNAi)* animals, suggesting these genes responded either to large-scale perturbations of *hnf4*, or were the result of large-scale effects in the whole organism.

Finally, to assess the expression of these genes within the whole organism, we scored the expression of the genes from each dose-dependent cluster on all the cells of our single cell dataset (Supplementary Figure 23C). While the low and mid dose dependent genes were expressed exclusively in cell types where *hnf4* is expressed, high-dose-dependent genes were also expressed by secretory 7 cells, a cell type where hnf4 is not expressed. It is likely, therefore, that high-dose-dependent genes are indirect effects of the treatment of *hnf4* knockdown.

Overall, our study of gene knock-down using different doses of dsRNA showcase the need to evaluate the effects of treatment dosage when designing knockdown experiments in planaria. We argue that performing knock-down experiments at several doses has a positive impact on evaluating the effect of a gene as it has the potential to tell apart direct effects from indirect effects, at the morphological and molecular level.

2. double knockdowns:

To further investigate the effects of *hnf4* in relation with *foxF-1* and *nkx2-2*, we performed single and double RNAi experiments in triplicates and assessed their phenotypes (Figure 8A). To analyse the

differentially regulated genes after each knockdown, we extracted and sequenced bulk RNA samples from each treatment and replicate (Figure 8A). We then compared DEGs (Wald test, two-sided, multiple comparison correction) between the single *hnf4* RNAi and the double *hnf4+nkx2−2* and *hnf4+foxF−1* (Figure 8E-G). We explored the DEGs that are reproducibly downregulated in several treatments (Figure 8H) and compared them with the DEGs in computationally dissected phagocytes and parenchyma of our single cell analysis (Figure 7C-E). We explored in detail the different groups of shared DEGs (Figure 8I) and scored their expression in our scRNA-seq data (Supplementary Figure 22C-U). Consistent with its stronger effects, the larger number of DEGs was found after hnf4 + nkx2-2 RNAi, with 356 genes unique to this treatment, and further 301 DEGs shared with other treatments (Figure 8I). Those genes had high scored expression in gut phagocytes as expected, and similar to the single *hnf4* RNAi. (Supplementary Figure 22D). Among the DEGs in the double *hnf4+nkx2-2*, two groups of 74 and 60 genes were downregulated in all bulk treatments with *hnf4* KD, with the latter also shared with the computationally dissected single-cell KD in phagocytes. Interestingly, these groups contained the previously mentioned low dose response genes (Supplementary Figure 23B-C, Supplementary Figure 22V). These results reveal a high overlap between the *hnf4* DEGs and the *nkx2-2* DEGs. In contrast, a group of 213 genes that was only discovered as downregulated in the gut phagocyte single-cell analysis displayed enrichment in gut phagocytes with broader expression in other cell types (Supplementary Figure 22I). This suggests that these genes are broadly expressed in more tissues but downregulated specifically in gut phagocytes after *hnf4* KD. Likely, their unchanged expression in other tissues averages out their downregulation in phagocytes in bulk conditions, therefore showing the greater specificity of the single-cell analysis. Another group of 89 genes were unique to the bulk *hnf*4 RNAi and displayed no enrichment in gut phagocytes, suggesting that these are the product of indirect responses. Indeed, this group of genes contained the largest amount of the previously mentioned high dose response genes (Supplementary Figure 23B,C, Supplementary Figure 22W), further suggesting that these are indirect DEGs. Importantly, none of these genes were found in neither of the single-cell analyses in computationally dissected phagocytes or parenchymal cells. These results indicate that the bulk double KD and the single-cell DGE analysis achieve similar results, with the single-cell analysis achieving a higher specificity, with less indirect effects. This results also show that *nkx2-2* and *hnf4* share many DEGs, suggesting that they synergize in their gene regulation activity.

We then focused on the genes differentially regulated after the double *hnf4* and *foxF-1* KD. Collectively, these genes showed high scored expression in both phagocytes and parenchymal cells (Supplementary Figure 22E), as expected. A group of 101 genes was unique to this treatment, with a high scored expression in parenchymal cells (Figure 8I, Supplementary Figure 22J), and further 208 DEGs shared with other treatments. Most of these genes were shared with *hnf4* RNAi treatments, and only a few genes were shared with the single-cell analysis in parenchymal cells (24, including the groups of 16, 3, 2, 2 and 1, Figure 8I). This indicates that, contrary to *nkx2-2* targets, *foxF-1* DEGs are largely independent from *hnf4*. Altogether, our analyses show that while bulk experiments may have more sensitivity, our computationally dissected parenchymal and phagocyte DGE achieve higher specificity. Interestingly, querying the interaction score of these gene sets in our networks revealed that genes downregulated only in the *hnf4(RNAi)+foxF-1(RNAi)* double knock-down had a higher interaction score with *foxF-1* than other DEGs (Figure 8J), which was not true for genes downregulated only in *hnf4(RNAi)+nkx2-2(RNAi)* (Figure 8J). This suggests that the independent response to the knockdown of a second TF is higher for *foxF-1* than for *nkx2-2*. Together with our analyses of overlapping gene sets, we interpret this as indicative of *nkx2-2* partnering with *hnf4* to drive differentiation of phagocytes, whereas *foxF-1* might share some downstream target genes with *hnf4* but drives parenchymal differentiation independently of *hnf4*. Overall, our computational

inference of GRNs followed by single and double knockdown provides new insights and formulates new hypotheses about how genome regulation drives planarian stem cell differentiation.

References

1. Langfelder, P. & Horvath, S. WGCNA: an R package for weighted correlation network analysis. *BMC Bioinformatics* **9**, 559 (2008).
2. Zhang, B. & Horvath, S. A general framework for weighted gene co-expression network analysis. *Stat Appl Genet Mol Biol* **4**, Article17 (2005).
3. Alvarez-Campos, P. *et al.* Annelid adult cell type diversity and their pluripotent cellular origins. *Nat Commun* **15**, 3194 (2024).
4. Love, M.I., Huber, W. & Anders, S. Moderated estimation of fold change and dispersion for RNA-seq data with DESeq2. *Genome Biol* **15**, 550 (2014).
5. Rosenberg, A.B. *et al.* Single-cell profiling of the developing mouse brain and spinal cord with split-pool barcoding. *Science* **360**, 176-182 (2018).
6. Macosko, E.Z. *et al.* Highly Parallel Genome-wide Expression Profiling of Individual Cells Using Nanoliter Droplets. *Cell* **161**, 1202-1214 (2015).
7. Marletaz, F. *et al.* Amphioxus functional genomics and the origins of vertebrate gene regulation. *Nature* **564**, 64-70 (2018).
8. Currie, K.W. & Pearson, B.J. Transcription factors lhx1/5-1 and pitx are required for the maintenance and regeneration of serotonergic neurons in planarians. *Development* **140**, 3577-3588 (2013).
9. Marz, M., Seebeck, F. & Bartscherer, K. A Pitx transcription factor controls the establishment and maintenance of the serotonergic lineage in planarians. *Development* **140**, 4499-4509 (2013).
10. Roberts-Galbraith, R.H., Brubacher, J.L. & Newmark, P.A. A functional genomics screen in planarians reveals regulators of whole-brain regeneration. *Elife* **5** (2016).
11. Barabasi, A.L. & Albert, R. Emergence of scaling in random networks. *Science* **286**, 509-512 (1999).
12. Yanai, I. *et al.* Genome-wide midrange transcription profiles reveal expression level relationships in human tissue specification. *Bioinformatics* **21**, 650-659 (2005).
13. Mantica, F. *et al.* Evolution of tissue-specific expression of ancestral genes across vertebrates and insects. *Nat Ecol Evol* **8**, 1140-1153 (2024).
14. Gu, Z., Eils, R. & Schlesner, M. Complex heatmaps reveal patterns and correlations in multidimensional genomic data. *Bioinformatics* **32**, 2847-2849 (2016).
15. Csardi, G. & Nepusz, T. The igraph software package for complex network research. *InterJournal* **Complex Systems**, 1695 (2006).
16. Hafemeister, C. & Halbritter, F. Single-cell RNA-seq differential expression tests within a sample should use pseudo-bulk data of pseudo-replicates. *bioRxiv*, 2023.2003.2028.534443 (2023).
17. Poulet, A., Kratkiewicz, A.J., Li, D. & van Wolfswinkel, J.C. Chromatin analysis of adult pluripotent stem cells reveals a unique stemness maintenance strategy. *Sci Adv* **9**, eadh4887 (2023).
18. Xu, Q. *et al.* ANANSE: an enhancer network-based computational approach for predicting key transcription factors in cell fate determination. *Nucleic Acids Res* **49**, 7966-7985 (2021).
19. van Heeringen, S.J. & Veenstra, G.J. GimmeMotifs: a de novo motif prediction pipeline for ChIP-sequencing experiments. *Bioinformatics* **27**, 270-271 (2011).
20. Heinz, S. *et al.* Simple combinations of lineage-determining transcription factors prime cis-regulatory elements required for macrophage and B cell identities. *Mol Cell* **38**, 576-589 (2010).
21. Rauluseviciute, I. *et al.* JASPAR 2024: 20th anniversary of the open-access database of transcription factor binding profiles. *Nucleic Acids Res* **52**, D174-D182 (2024).
22. Lambert, S.A. *et al.* Similarity regression predicts evolution of transcription factor sequence specificity. *Nat Genet* **51**, 981-989 (2019).

23. Emms, D.M. & Kelly, S. OrthoFinder: phylogenetic orthology inference for comparative genomics. *Genome Biol* **20**, 238 (2019).
24. Neiro, J., Sridhar, D., Dattani, A. & Aboobaker, A. Identification of putative enhancer-like elements predicts regulatory networks active in planarian adult stem cells. *Elife* **11** (2022).
25. Satpathy, A.T. *et al.* Massively parallel single-cell chromatin landscapes of human immune cell development and intratumoral T cell exhaustion. *Nat Biotechnol* **37**, 925-936 (2019).
26. Rozanski, A. *et al.* PlanMine 3.0-improvements to a mineable resource of flatworm biology and biodiversity. *Nucleic Acids Res* **47**, D812-D820 (2019).
27. Cheng, L.C. *et al.* Cellular, ultrastructural and molecular analyses of epidermal cell development in the planarian Schmidtea mediterranea. *Dev Biol* **433**, 357-373 (2018).
28. Tu, K.C. *et al.* Egr-5 is a post-mitotic regulator of planarian epidermal differentiation. *Elife* **4**, e10501 (2015).
29. Cowles, M.W. *et al.* Genome-wide analysis of the bHLH gene family in planarians identifies factors required for adult neurogenesis and neuronal regeneration. *Development* **140**, 4691-4702 (2013).
30. Kao, D., Felix, D. & Aboobaker, A. The planarian regeneration transcriptome reveals a shared but temporally shifted regulatory program between opposing head and tail scenarios. *BMC Genomics* **14**, 797 (2013).
31. Stuart, T. *et al.* Comprehensive Integration of Single-Cell Data. *Cell* **177**, 1888-1902 e1821 (2019).
32. Stuart, T. & Satija, R. Integrative single-cell analysis. *Nat Rev Genet* **20**, 257-272 (2019).
33. Stuckemann, T. *et al.* Antagonistic Self-Organizing Patterning Systems Control Maintenance and Regeneration of the Anteroposterior Axis in Planarians. *Dev Cell* **40**, 248-263 e244 (2017).
34. Cowles, M.W., Omuro, K.C., Stanley, B.N., Quintanilla, C.G. & Zayas, R.M. COE loss-of-function analysis reveals a genetic program underlying maintenance and regeneration of the nervous system in planarians. *PLoS Genet* **10**, e1004746 (2014).
35. Akheralie, Z., Scidmore, T.J. & Pearson, B.J. aristaless-like homeobox-3 is wound induced and promotes a low-Wnt environment required for planarian head regeneration. *Development* **150** (2023).
36. Patro, R., Duggal, G., Love, M.I., Irizarry, R.A. & Kingsford, C. Salmon provides fast and bias-aware quantification of transcript expression. *Nat Methods* **14**, 417-419 (2017).
