# Supplementary material for: Multimodal single cell analyses reveal gene networks of planarian stem cell differentiation
Source: Nat Commun. 2025 Nov 27;16:10683. doi: 10.1038/s41467-025-65712-0 (PMC12660999; doi:10.1038/s41467-025-65712-0)
Supplement: Supplementary file 3 — Description of Additional Supplementary Files [file 41467_2025_65712_MOESM3_ESM.pdf]

Supplementary Data Descriptions

Supplementary Data 1. Details on sequencing libraries used in this study.

Supplementary Data 2. Gene and functional annotation used in this study, including transcription factor annotation and WGCNA module membership.

Supplementary Data 3. Cell type annotation of Seurat clusters of the main scRNA-seq dataset in this study.

Supplementary Data 4. Table of co-expression modules detected by WGCNA in the main scRNA-seq dataset in this study.

Supplementary Data 5. Boxplots of normalised gene counts on each cell type, for each WGCNA module of gene co-expression. Centre line, median; box limits, upper and lower quartiles; whiskers, 1.5x interquartile range; points, outliers.

Supplementary Data 6. Feature plots of thirty genes per WGCNA module of gene co-expression, randomly sampled for each module.

Supplementary Data 7. Bar plots of Gene Ontology enrichment of each WGCNA module of gene co-expression.

Supplementary Data 8. Motif clustering and motif enrichment analysis on the promoters of the genes from each WGCNA module of gene co-expression.

Supplementary Data 9. Table of co-accessibility modules detected by WGCNA in the main scATAC-seq dataset in this study.

Supplementary Data 10. Table of OCRs and associated genes from the different co-accessibility and co-expression modules.

Supplementary Data 11. A: Graphical overview of the splitting of the scATAC-seq into pseudo-replicates and the differential chromatin accessibility analysis. B: Volcano plots showing log fold change (x-axis) and -log p. adjusted (y axis) of each cell type-specific (one-vs-all) differential chromatin accessibility analysis, one for each broad cell type. C: Chromatin profile of the cell type-specific (one-vs-all) differential OCRs. D: Volcano plots showing log fold change (x-axis) and -log p. adjusted (y axis) of each differentiated cell type (one-vs-neoblasts) differential chromatin accessibility analysis. E: Chromatin plot of the differentiated cell type (one-vs-neoblast) differentially accessible OCRs. F: (top) Example feature plots of cell type-specific (one-versus-all) differentially accessible OCRs; (bottom) Example feature plots of differentiated cell type (one-versus-neoblast) differentially accessible OCRs. G: Venn diagrams of the overlap between cell type-specific and differentiated cell type OCRs. H: Scale-free topology model fit of different networks obtained from raising the gene-wise correlations of the pseudobulk scATAC-seq data (one-vs-neoblast OCRs) to fifteen different soft power thresholds. I: Mean connectivity of different networks obtained from raising the gene-wise correlations of the pseudobulk scATAC-seq data (one-vs-neoblast OCRs) to fifteen different soft power thresholds. J: Heatmap of chromatin accessibility of differentiated cell type OCRs from different WGCNA co-accessibility modules across broad cell types in *S. mediterranea*.

OCRs in rows, cell types in columns. A random subset of twenty OCRs per module is shown. On the right side, stacked bar plot of average chromatin accessibility of each co-accessibility module in each broad cell type (see Methods; Supplementary Note 1). On the right-most side, bar plot showing number of OCRs per co-accessibility module. K: Heatmap showing gene expression profile of all the genes associated with the differentiated cell type (one-vs-neoblast) OCRs. Genes have been sorted and arranged based on the co-accessibility modules of their associated OCRs. Genes in rows, cell types in columns. Columns have been clustered as in G. L: Box plot showing correlation of genes to the co-accessibility profile of modules from their associated OCRs. Every box corresponds to a co-accessibility module. Every point is a gene. Genes labelled in grey do not appear in Figure 3G. Genes labelled in red do. Centre line, median; box limits, upper and lower quartiles; whiskers, 1.5x interquartile range; points, data points. M: Heatmap of gene expression of genes associated to OCRs from different co-accessibility modules as in F. Genes in rows, cell types in columns. A subset of top-correlating genes is shown (see Supplementary Note 1 for details).

Supplementary Data 12. Lists of differential regions of open chromatin (OCRs) between every differentiated cell type and the rest, in a one-versus-all manner.

Supplementary Data 13. Lists of differential regions of open chromatin (OCRs) between every differentiated cell type and neoblasts.

Supplementary Data 14. List of OCR/module associations resulting from running WGCNA on one-versus-neoblasts OCRs.

Supplementary Data 15. List of species used in orthology transferring of TFs using gimmemotifs motif2factors.

Supplementary Data 16. List of outputs of ANANSE influence between the network of every differentiated cell type and the neoblast network.

Supplementary Data 17. Top targets of five example TFs of the differential networks when comparing neoblasts to every differentiated cell type. From top row to bottom row and following colour code as in the rest of the manuscript: early epidermal progenitors, epidermis, phagocytes, basal/goblet cells, muscle cells, neurons, parenchymal cells, protonephridia cells, and secretory cells.

Supplementary Data 18. Heatmap showing presence/absence of putative predicted interaction between the top influential TFs of each fate and the targets with an identified gene symbol in the literature.

Supplementary Data 19. Table of the putative predicted interactions between the top influential TFs of each fate and the targets with an identified gene symbol in the literature.

Supplementary Data 20. Differential Gene Expression data and results for the reanalysis of pax2/5/8-1 and soxP-3 knockdowns.

Supplementary Data 21. Differential Gene Expression data and results for the reanalysis of p53 knockdown.

Supplementary Data 22. Data and results from re-analysing the planarian anteroposterior transcriptomics data.

Supplementary Data 23. Differential Gene Expression data and results for the reanalysis of *coe* knockdown.

Supplementary Data 24. Data and results for the reanalysis of *prep* knockdown.

Supplementary Data 25. List of influential TFs and their associated set of co-influence.

Supplementary Data 26. Differential Gene Expression data and results for the reanalysis of *alx3-1* knockdown.

Supplementary Data 27. Whole mount in situ hybridisation count data for control and *alx3-1* knockdown.

Supplementary Data 28. Cell type annotation of Seurat clusters of the *hnf4(RNAi)* knock-down scRNA-seq dataset, including number of cells from each sample for each cluster.

Supplementary Data 29. List of gene markers used to calculate the neoblast gene score and the phagocyte gene score.

Supplementary Data 30. List of Differentially Expressed Genes (DEGs) for all broad cell types when comparing broad cell types of control and *hnf4*i samples.

Supplementary Data 31. Survival and phenotype data for the double knockdowns of *hnf4*, *hnf4+gfp*, *hnf4+nkx2-2*, and *hnf4+foxF-1*.

Supplementary Data 32. Differential Gene Expression data and results for the double knockdowns of *hnf4*, *hnf4+gfp*, *hnf4+nkx2-2*, and *hnf4+foxF-1*.
