# Supplementary figures and images for: Multimodal single cell analyses reveal gene networks of planarian stem cell differentiation
Source: Nat Commun. 2025 Nov 27;16:10683. doi: 10.1038/s41467-025-65712-0 (PMC12660999; doi:10.1038/s41467-025-65712-0)

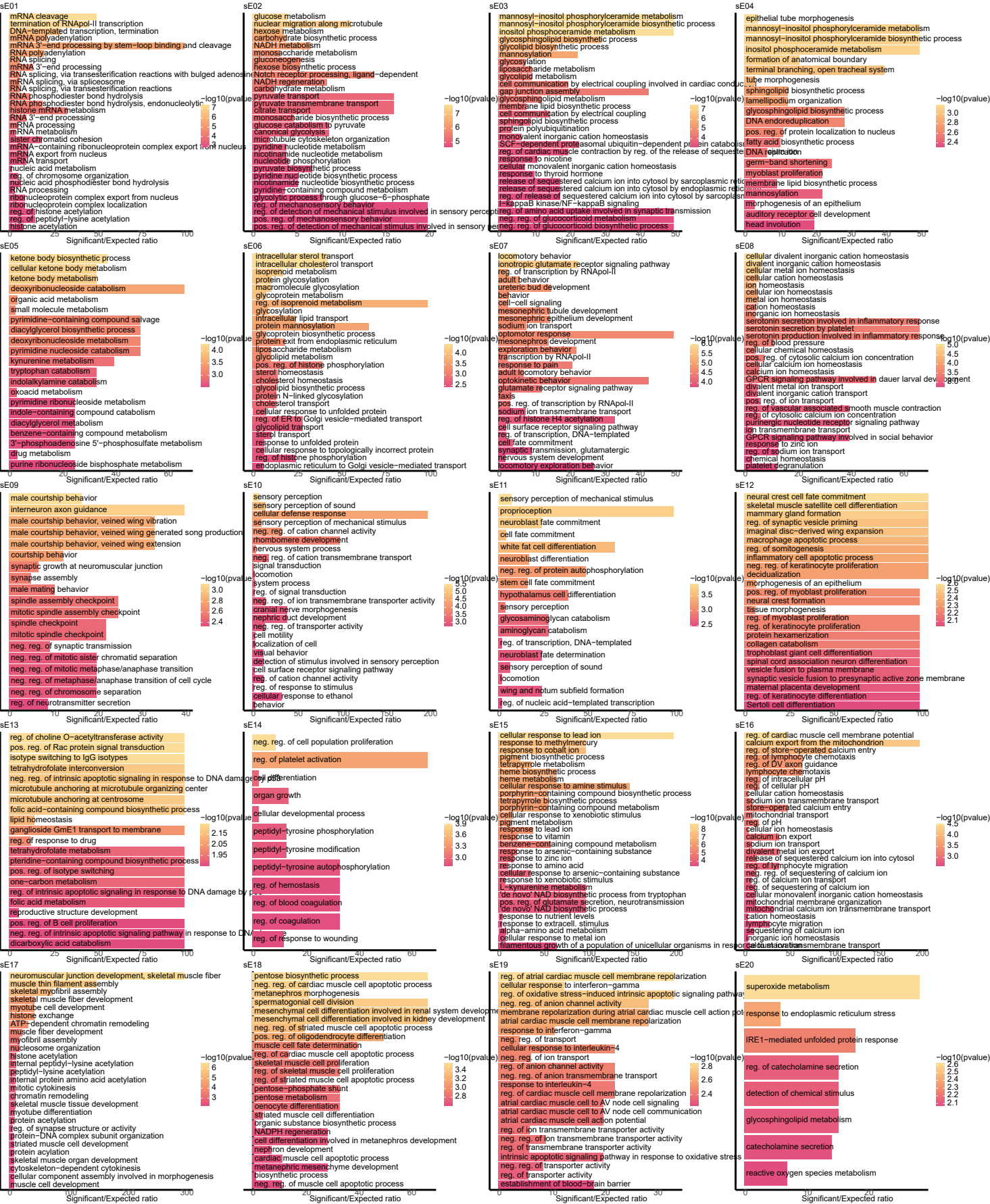

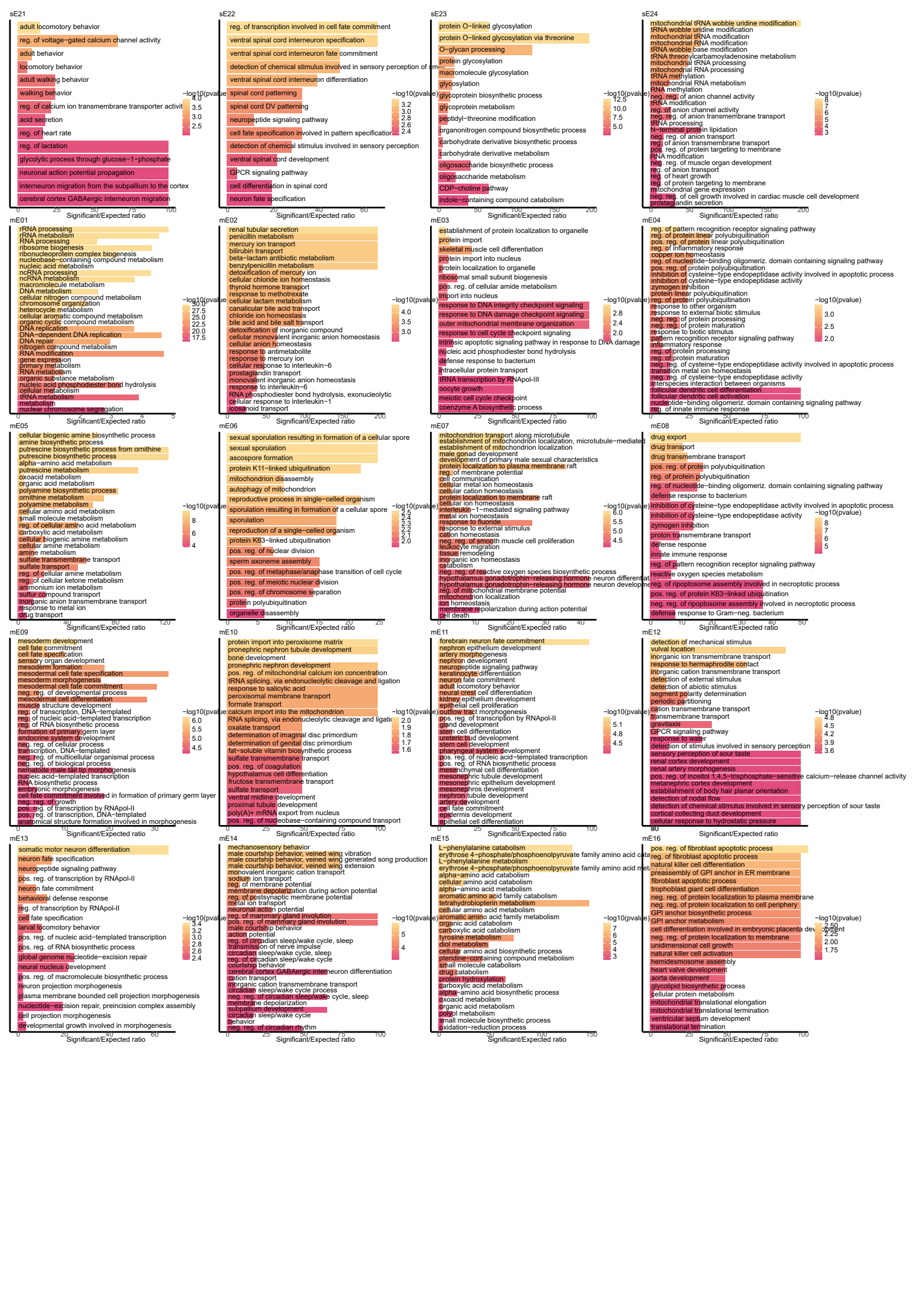

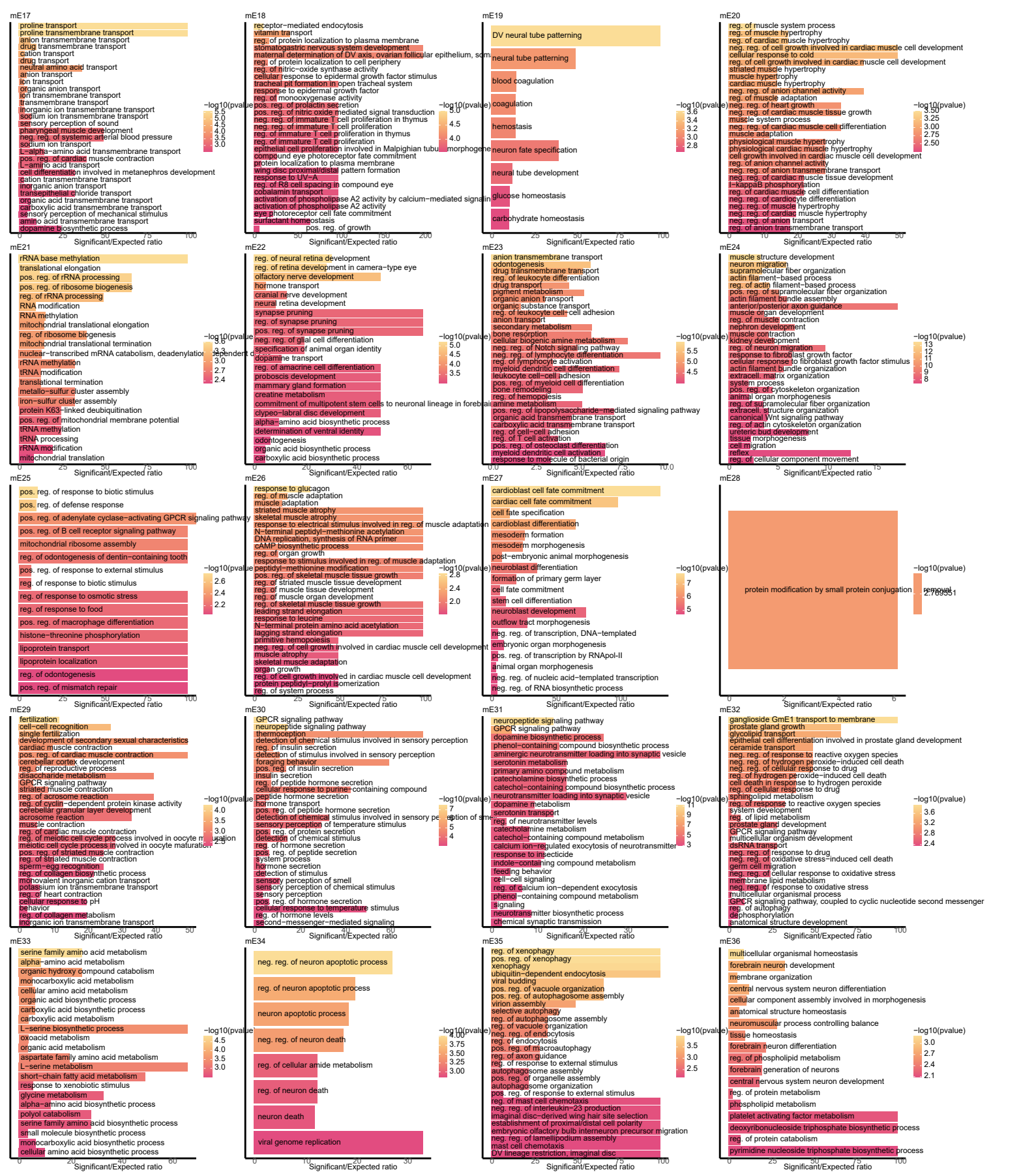

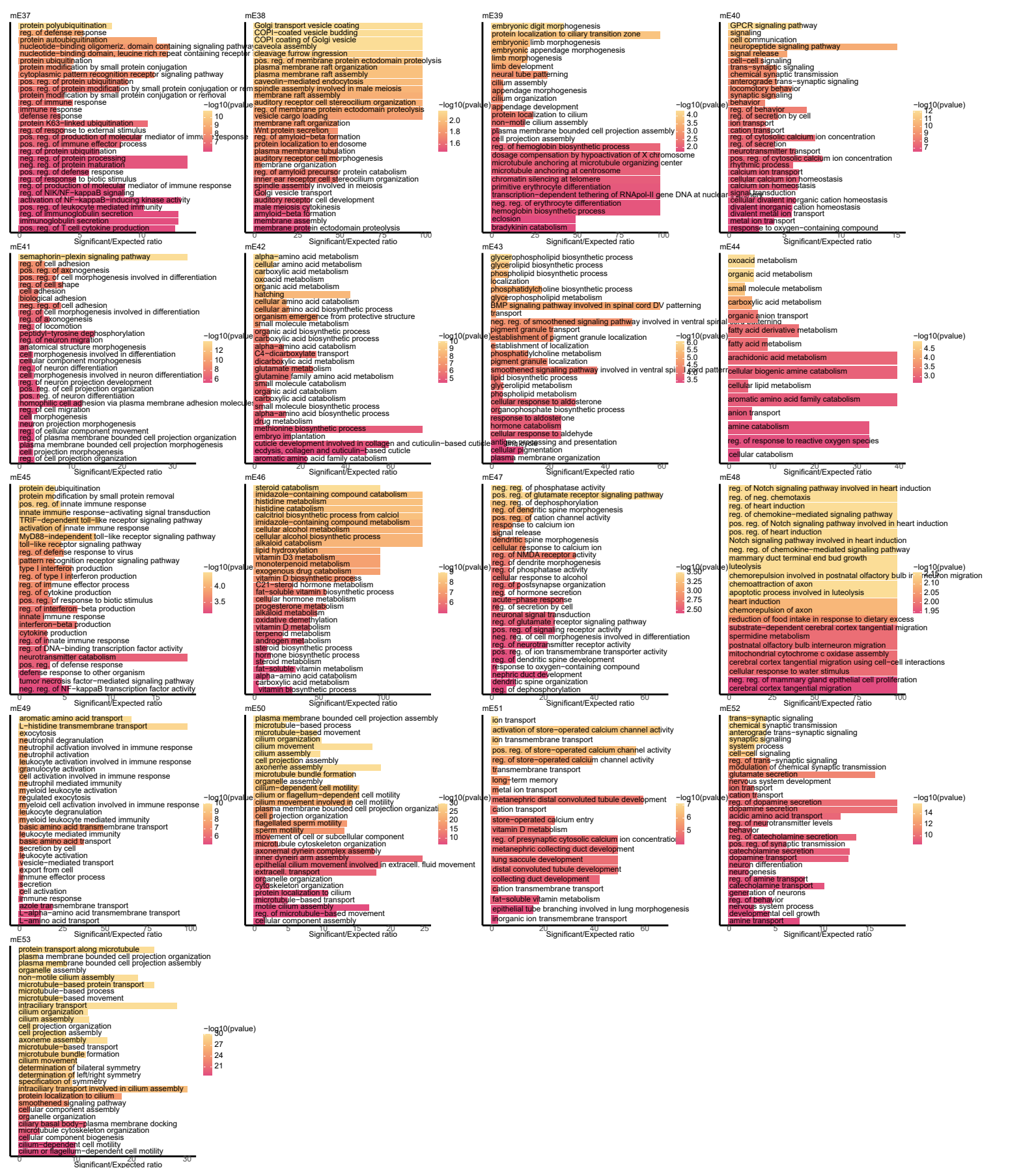

Supplement: Supplementary file 10 — Supplementary Data 7 [file 41467_2025_65712_MOESM10_ESM.pdf]

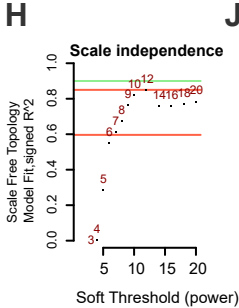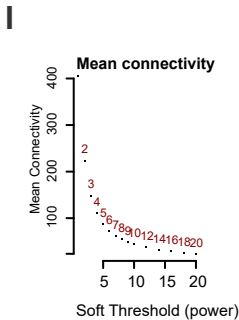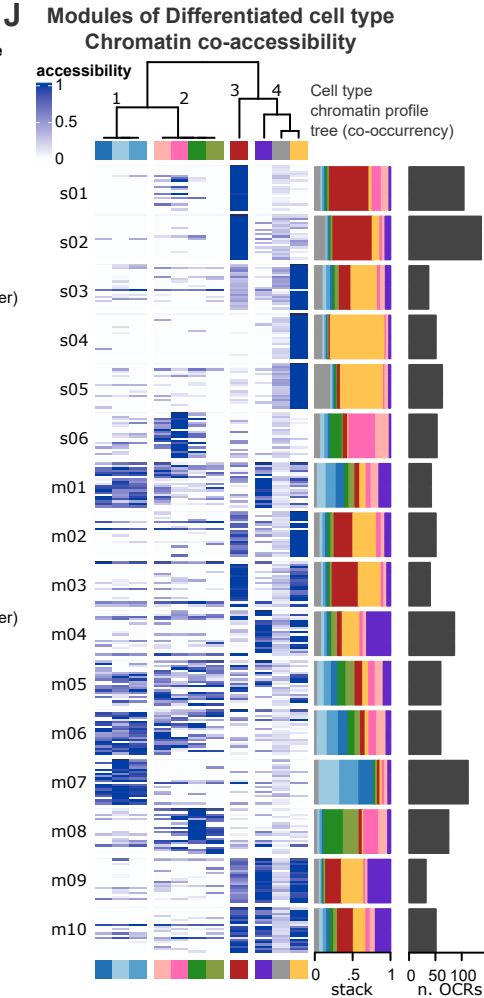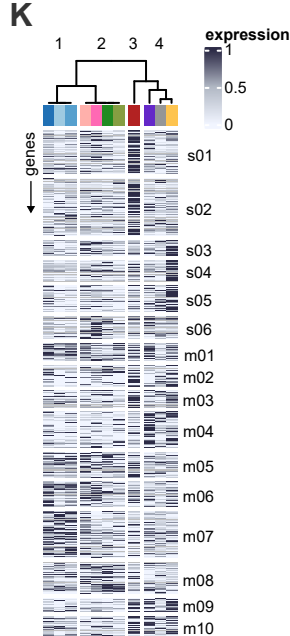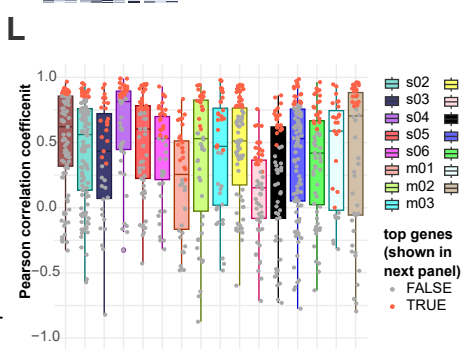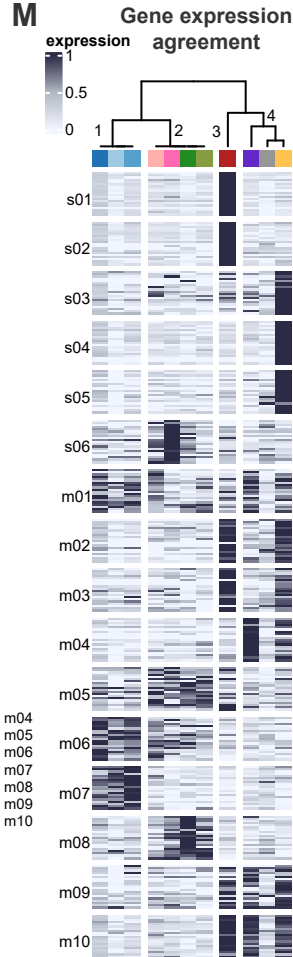

Supplement: Supplementary file 14 — Supplementary Data 11 [file 41467_2025_65712_MOESM14_ESM.pdf]

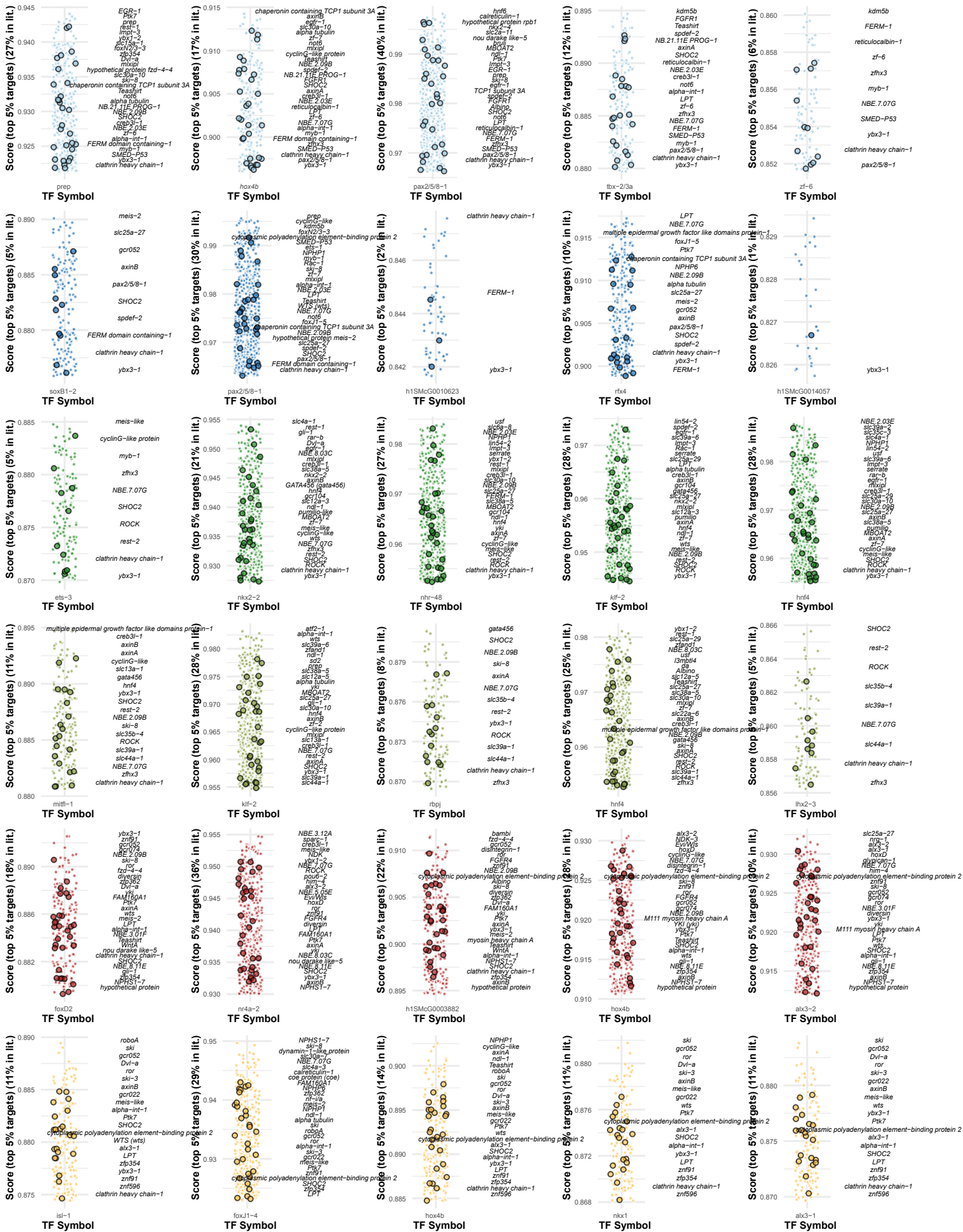

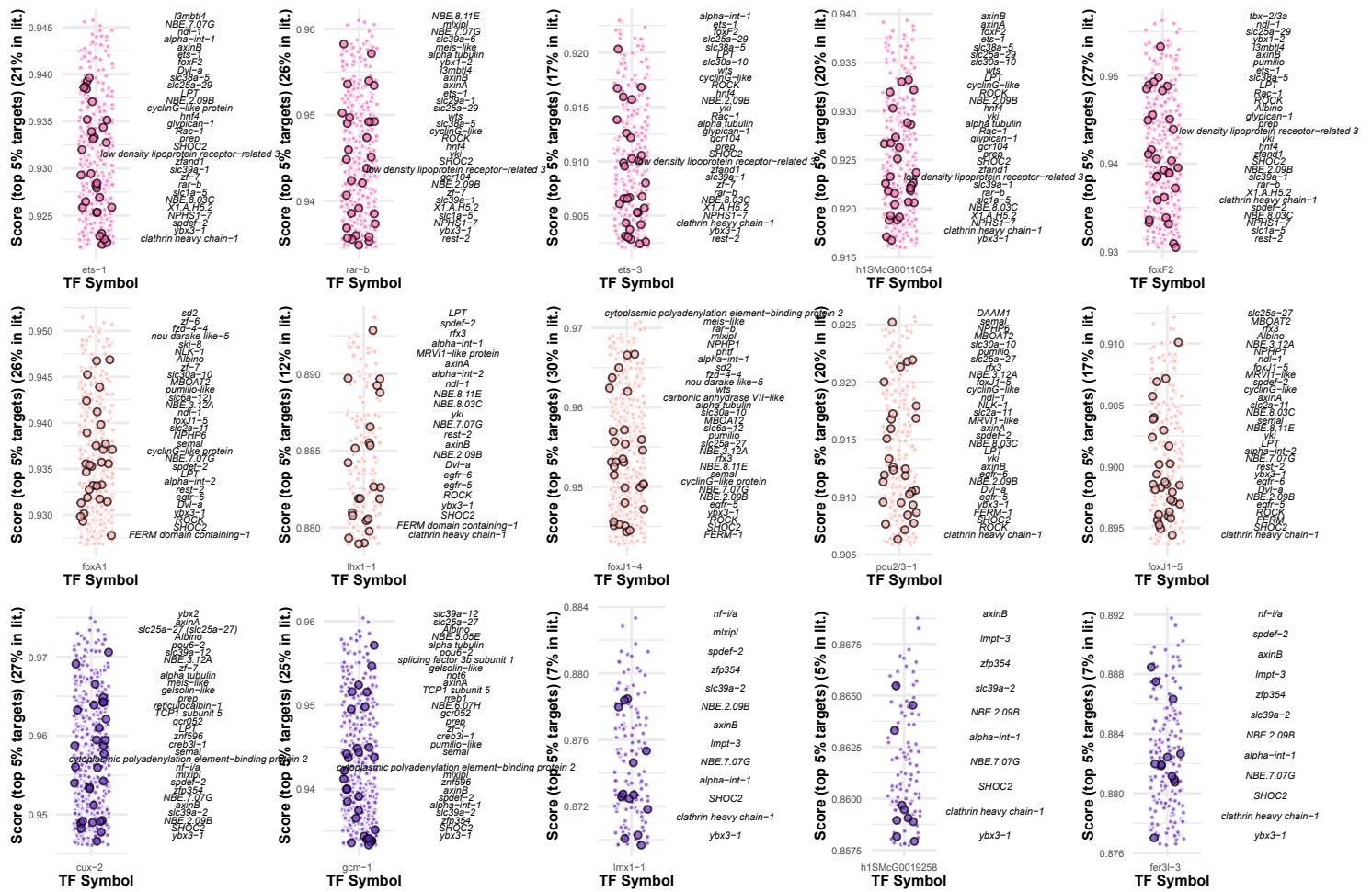

Supplement: Supplementary file 20 — Supplementary Data 17 [file 41467_2025_65712_MOESM20_ESM.pdf]

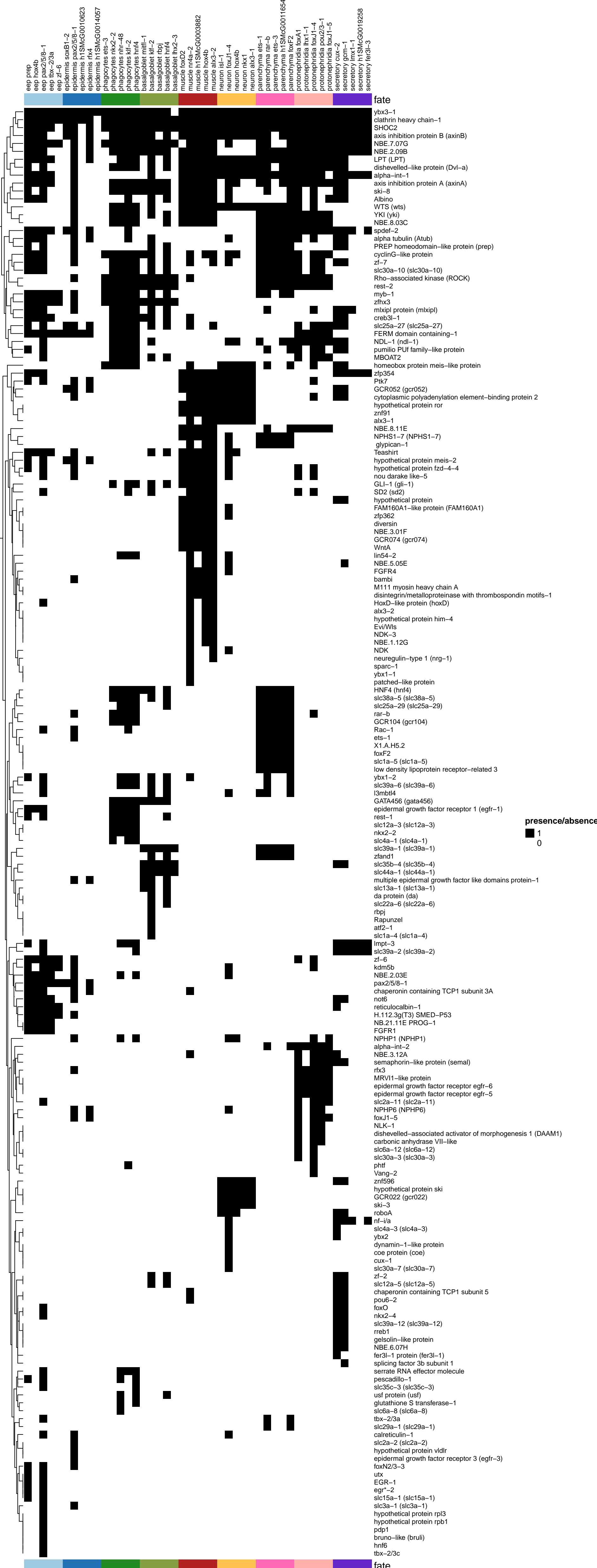

Supplement: Supplementary file 21 — Supplementary Data 18 [file 41467_2025_65712_MOESM21_ESM.pdf]
