# Supplementary material for: Multimodal single cell analyses reveal gene networks of planarian stem cell differentiation
Source: Nat Commun. 2025 Nov 27;16:10683. doi: 10.1038/s41467-025-65712-0 (PMC12660999; doi:10.1038/s41467-025-65712-0)
Supplement: Supplementary file 11 — Supplementary Data 8 [file 41467_2025_65712_MOESM11_ESM.pdf]

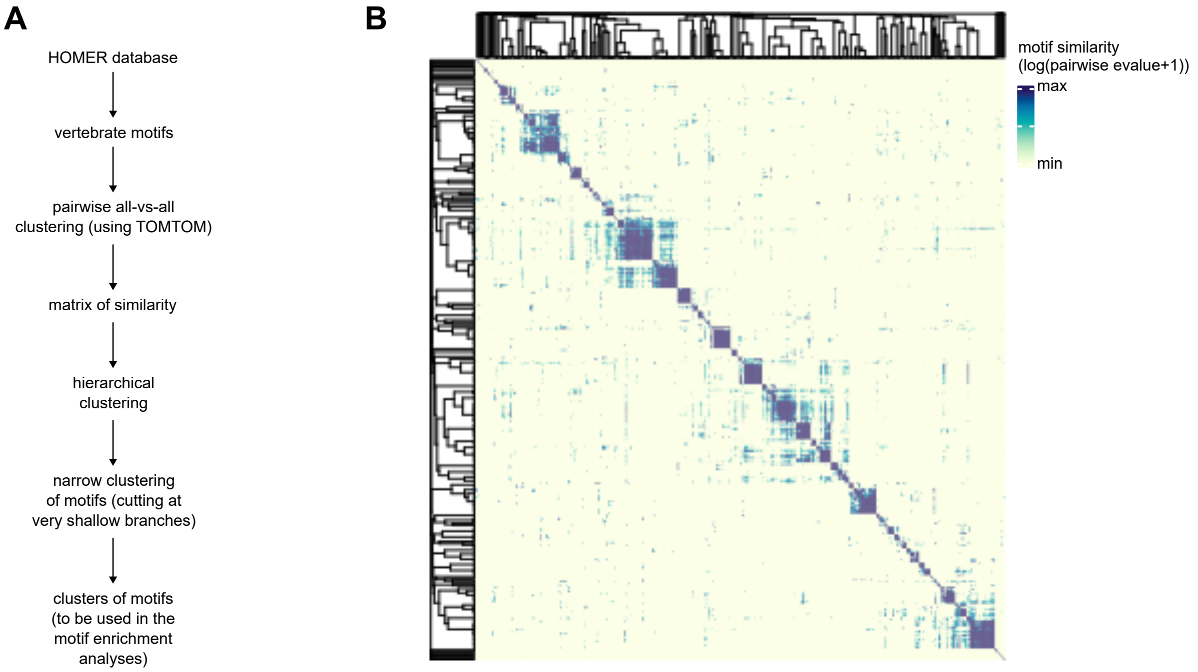
A
HOMER database
vertebrate motifs
pairwise all-vs-all clustering (using TOMTOM)
matrix of similarity
hierarchical clustering
narrow clustering of motifs (cutting at very shallow branches)
clusters of motifs (to be used in the motif enrichment analyses)
B
motif similarity (log(pairwise evalue+1))
max
min

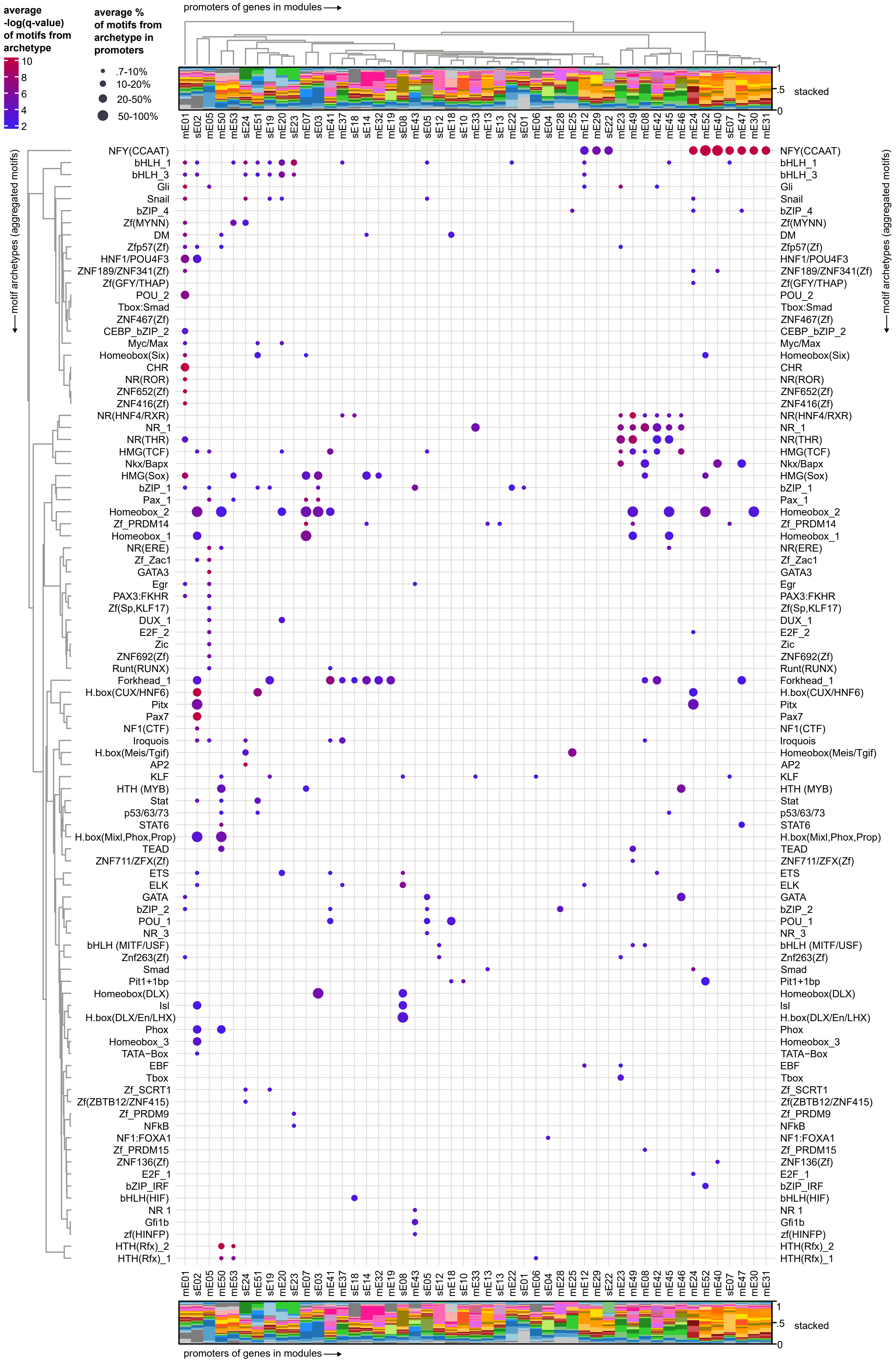
average -log(q-value) of motifs from archetype
10
8
6
4
2
average % of motifs from archetype in promoters
.7-10%
10-20%
20-50%
50-100%
promoters of genes in modules
stacked
1
.5
0
mE01 sE02 mE05 mE50 mE53 sE24 mE51 sE19 mE20 sE23 mE07 sE03 mE41 mE37 sE18 sE14 mE32 mE19 sE08 mE43 sE05 sE12 mE18 sE10 mE33 mE13 sE13 mE22 sE01 mE06 sE04 mE28 mE25 mE12 mE29 sE22 mE23 mE49 mE08 mE42 mE45 mE46 mE24 mE52 mE40 sE07 mE47 mE30 mE31
motif archetypes (aggregated motifs)
NFY(CCAAT)
bHLH_1
bHLH_3
Gli
Snail
bZIP_4
Zf(MYNN)
DM
Zfp57(Zf)
HNF1/POU4F3
ZNF189/ZNF341(Zf)
Zf(GFY/THAP)
POU_2
Tbox:Smad
ZNF467(Zf)
CEBP_bZIP_2
Myc/Max
Homeobox(Six)
CHR
NR(ROR)
ZNF652(Zf)
ZNF416(Zf)
NR(HNF4/RXR)
NR_1
NR(THR)
HMG(TCF)
Nkx/Bapx
HMG(Sox)
bZIP_1
Pax_1
Homeobox_2
Zf_PRDM14
Homeobox_1
NR(ERE)
Zf_Zac1
GATA3
Egr
PAX3:FKHR
Zf(Sp,KLF17)
DUX_1
E2F_2
Zic
ZNF692(Zf)
Runt(RUNX)
Forkhead_1
H.box(CUX/HNF6)
Pitx
Pax7
NF1(CTF)
Iroquois
H.box(Meis/Tgif)
AP2
KLF
HTH (MYB)
Stat
p53/63/73
STAT6
H.box(Mixl,Phox,Prop)
TEAD
ZNF711/ZFX(Zf)
ETS
ELK
GATA
bZIP_2
POU_1
NR_3
bHLH (MITF/USF)
Znf263(Zf)
Smad
Pit1+1bp
Homeobox(DLX)
Isl
H.box(DLX/En/LHX)
Phox
Homeobox_3
TATA−Box
EBF
Tbox
Zf_SCRT1
Zf(ZBTB12/ZNF415)
Zf_PRDM9
NFkB
NF1:FOXA1
Zf_PRDM15
ZNF136(Zf)
E2F_1
bZIP_IRF
bHLH(HIF)
NR 1
Gfi1b
zf(HINFP)
HTH(Rfx)_2
HTH(Rfx)_1
