# Supplementary material for: Multimodal single cell analyses reveal gene networks of planarian stem cell differentiation
Source: Nat Commun. 2025 Nov 27;16:10683. doi: 10.1038/s41467-025-65712-0 (PMC12660999; doi:10.1038/s41467-025-65712-0)
Supplement: Supplementary file 36 — Reporting Summary [file 41467_2025_65712_MOESM36_ESM.pdf]

nature portfolio

Corresponding author(s): Alberto Perez-Posada, Jordi Solana Garcia

Last updated by author(s): 10/03/2025

Reporting Summary

Nature Portfolio wishes to improve the reproducibility of the work that we publish. This form provides structure for consistency and transparency in reporting. For further information on Nature Portfolio policies, see our Editorial Policies and the Editorial Policy Checklist.

Statistics

For all statistical analyses, confirm that the following items are present in the figure legend, table legend, main text, or Methods section.

n/a	Confirmed	
☐	☒	The exact sample size (n) for each experimental group/condition, given as a discrete number and unit of measurement
☐	☒	A statement on whether measurements were taken from distinct samples or whether the same sample was measured repeatedly
☐	☒	The statistical test(s) used AND whether they are one- or two-sided<br>*Only common tests should be described solely by name; describe more complex techniques in the Methods section.*
☒	☐	A description of all covariates tested
☐	☒	A description of any assumptions or corrections, such as tests of normality and adjustment for multiple comparisons
☐	☒	A full description of the statistical parameters including central tendency (e.g. means) or other basic estimates (e.g. regression coefficient) AND variation (e.g. standard deviation) or associated estimates of uncertainty (e.g. confidence intervals)
☐	☒	For null hypothesis testing, the test statistic (e.g. F, t, r) with confidence intervals, effect sizes, degrees of freedom and P value noted<br>*Give P values as exact values whenever suitable.*
☒	☐	For Bayesian analysis, information on the choice of priors and Markov chain Monte Carlo settings
☒	☐	For hierarchical and complex designs, identification of the appropriate level for tests and full reporting of outcomes
☐	☒	Estimates of effect sizes (e.g. Cohen's d, Pearson's r), indicating how they were calculated

Our web collection on statistics for biologists contains articles on many of the points above.

Software and code

Policy information about availability of computer code

Data collection	Data publicly available was downloaded using fastq-dump v3.1.1<br>No software was used to collect our newly generated data.
Data analysis	Dropseqtools v2.4.0<br>cellranger-atac v2.1.0<br>R v4.0.3<br>R v4.4.1<br>Seurat v4.3.0<br>Signac v1.14.0<br>STAR 2.7.11b<br>Kallisto v0.51.1<br>Salmon v1.10.0<br>Bedtools v2.29.1<br>Bowtie2 v2.5.4<br>WGCNA v1.73<br>Igraph v2.1.3<br>ANANSE v0.5.1

For manuscripts utilizing custom algorithms or software that are central to the research but not yet described in published literature, software must be made available to editors and reviewers. We strongly encourage code deposition in a community repository (e.g. GitHub). See the Nature Portfolio guidelines for submitting code & software for further information.

Data

Policy information about availability of data

All manuscripts must include a data availability statement. This statement should provide the following information, where applicable:

- Accession codes, unique identifiers, or web links for publicly available datasets
- A description of any restrictions on data availability
- For clinical datasets or third party data, please ensure that the statement adheres to our policy

The datasets supporting the conclusions of this article are available in:
GEO (whole project, including scRNA-seq reads, scATAC-seq reads, ATAC-seq reads, bulk RNA-seq reads, as well as processed files such as .RDS Seurat objects): GSE274286.
Code, including markdowns, functions, and scripts, for all analyses: https://github.com/scbe-lab/regulatory_logic

Research involving human participants, their data, or biological material

Policy information about studies with human participants or human data. See also policy information about sex, gender (identity/presentation), and sexual orientation and race, ethnicity and racism.

Reporting on sex and gender	n/a
Reporting on race, ethnicity, or other socially relevant groupings	n/a
Population characteristics	n/a
Recruitment	n/a
Ethics oversight	n/a

Note that full information on the approval of the study protocol must also be provided in the manuscript.

Field-specific reporting

Please select the one below that is the best fit for your research. If you are not sure, read the appropriate sections before making your selection.

☒ Life sciences ☐ Behavioural & social sciences ☐ Ecological, evolutionary & environmental sciences

For a reference copy of the document with all sections, see nature.com/documents/nr-reporting-summary-flat.pdf

Life sciences study design

All studies must disclose on these points even when the disclosure is negative.

Sample size	Animals of different sizes were collected at random, effect sizes are known based on previous observations in previous publications (Garcia-Castro et al., 2021 Genome Biology; Emili et al., 2025 Science Advances).
Data exclusions	We excluded two samples from our double knockdown analysis based on PCA analysis. These samples deviated from the general trend of other replicates from their same conditions.
Replication	Knockdown experiments were performed in replicates. We did two replicates per condition for the single cell knockdwon experiment, and three replicates per condition for the double knockdown RNA-Seq experiments. Knockdown and In Situ Hybridisation experiments were performed in multiple animals, eight per condition.
Randomization	Animals were collected at random sizes, based on previous observations from previous publications (Emili et al., 2025 Science Advances).
Blinding	For phenotype observations, all samples and specimens from the same experiment were allocated to the same researcher that performed the experiment.

Reporting for specific materials, systems and methods

We require information from authors about some types of materials, experimental systems and methods used in many studies. Here, indicate whether each material, system or method listed is relevant to your study. If you are not sure if a list item applies to your research, read the appropriate section before selecting a response.

Materials & experimental systems

n/a	Involved in the study
☒	☐ Antibodies
☒	☐ Eukaryotic cell lines
☒	☐ Palaeontology and archaeology
☐	☒ Animals and other organisms
☒	☐ Clinical data
☒	☐ Dual use research of concern
☒	☐ Plants

Methods

n/a	Involved in the study
☒	☐ ChIP-seq
☒	☐ Flow cytometry
☒	☐ MRI-based neuroimaging

Animals and other research organisms

Policy information about studies involving animals; ARRIVE guidelines recommended for reporting animal research, and Sex and Gender in Research

Laboratory animals: We used planarians of the species Schmidtea mediterranea, asexual strain Berlin-1

Wild animals: *Provide details on animals observed in or captured in the field; report species and age where possible. Describe how animals were caught and transported and what happened to captive animals after the study (if killed, explain why and describe method; if released, say where and when) OR state that the study did not involve wild animals.*

Reporting on sex: Planarians used in this study are asexual, and they are invertebrates that fall outside of the UK 1986 Animal Act's legislation and the EU Directive 2010/63/EU of the European Parliament and of the Council of 22 September 2010 on the protection of animals for scientific purposes.

Field-collected samples: *For laboratory work with field-collected samples, describe all relevant parameters such as housing, maintenance, temperature, photoperiod and end-of-experiment protocol OR state that the study did not involve samples collected from the field.*

Ethics oversight: No ethical approval or guidance was needed. Planarians are invertebrates that fall outside of the UK 1986 Animal Act's legislation and the EU Directive 2010/63/EU of the European Parliament and of the Council of 22 September 2010 on the protection of animals for scientific purposes.

Note that full information on the approval of the study protocol must also be provided in the manuscript.

Plants

Seed stocks: *Report on the source of all seed stocks or other plant material used. If applicable, state the seed stock centre and catalogue number. If plant specimens were collected from the field, describe the collection location, date and sampling procedures.*

Novel plant genotypes: *Describe the methods by which all novel plant genotypes were produced. This includes those generated by transgenic approaches, gene editing, chemical/radiation-based mutagenesis and hybridization. For transgenic lines, describe the transformation method, the number of independent lines analyzed and the generation upon which experiments were performed. For gene-edited lines, describe the editor used, the endogenous sequence targeted for editing, the targeting guide RNA sequence (if applicable) and how the editor was applied.*

Authentication: *Describe any authentication procedures for each seed stock used or novel genotype generated. Describe any experiments used to assess the effect of a mutation and, where applicable, how potential secondary effects (e.g. second site T-DNA insertions, mosiacism, off-target gene editing) were examined.*
