# Supplementary material for: Multimodal single cell analyses reveal gene networks of planarian stem cell differentiation
Source: Nat Commun. 2025 Nov 27;16:10683. doi: 10.1038/s41467-025-65712-0 (PMC12660999; doi:10.1038/s41467-025-65712-0)

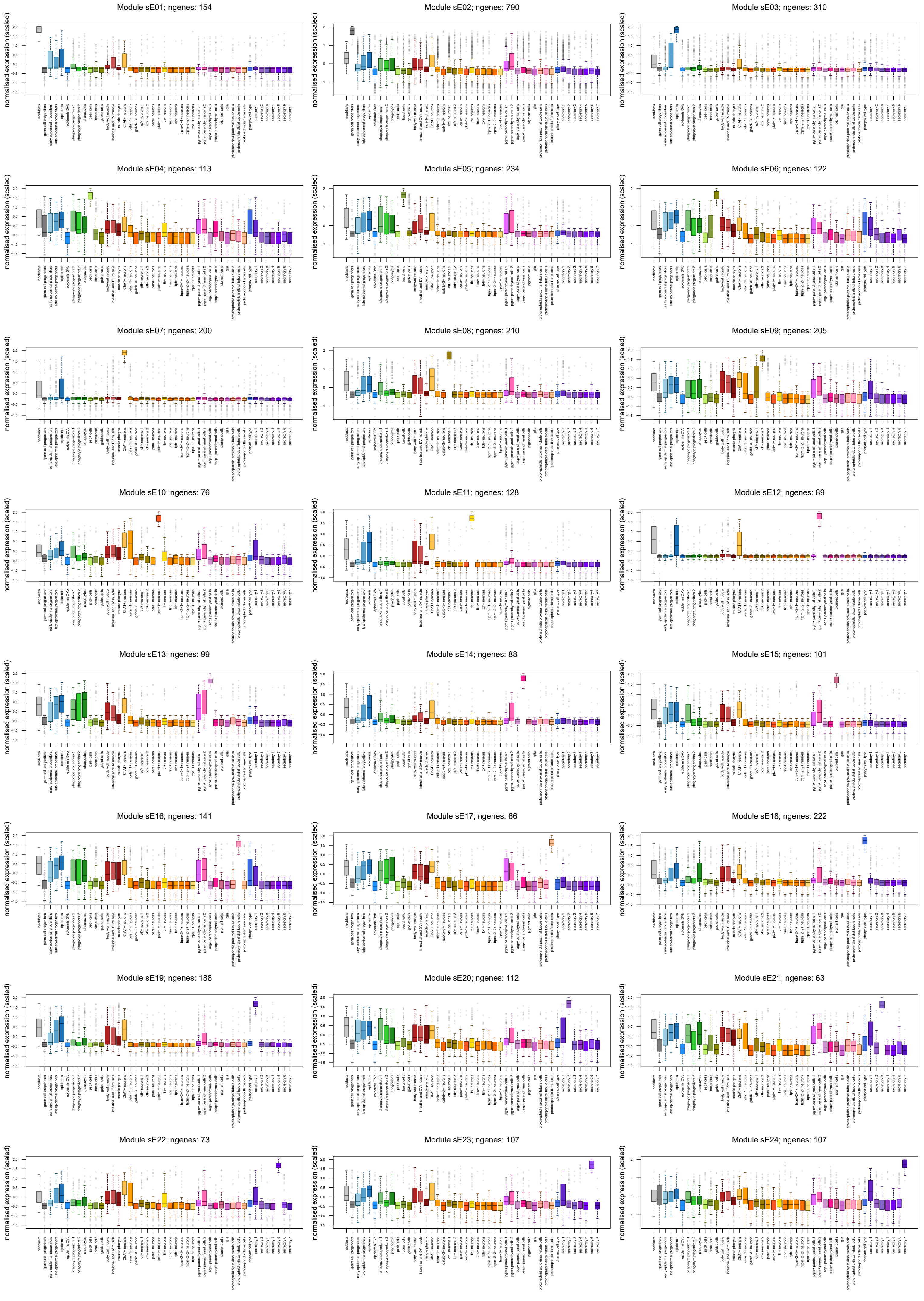
Module sE01; ngenes: 154
Module sE02; ngenes: 790
Module sE03; ngenes: 310
Module sE04; ngenes: 113
Module sE05; ngenes: 234
Module sE06; ngenes: 122
Module sE07; ngenes: 200
Module sE08; ngenes: 210
Module sE09; ngenes: 205
Module sE10; ngenes: 76
Module sE11; ngenes: 128
Module sE12; ngenes: 89
Module sE13; ngenes: 99
Module sE14; ngenes: 88
Module sE15; ngenes: 101
Module sE16; ngenes: 141
Module sE17; ngenes: 66
Module sE18; ngenes: 222
Module sE19; ngenes: 188
Module sE20; ngenes: 112
Module sE21; ngenes: 63
Module sE22; ngenes: 73
Module sE23; ngenes: 107
Module sE24; ngenes: 107
normalised expression (scaled)

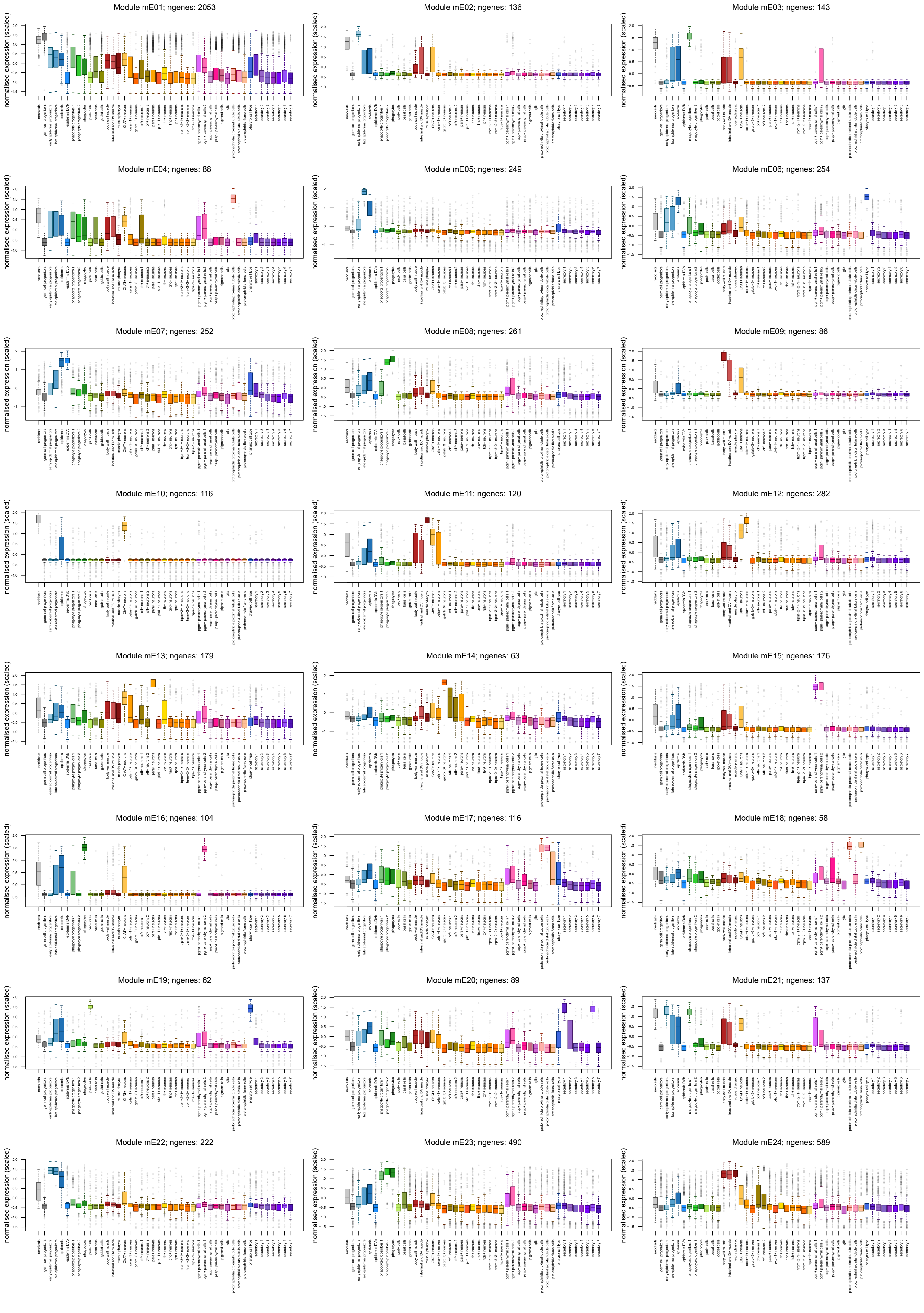
Module mE01; ngenes: 2053
Module mE02; ngenes: 136
Module mE03; ngenes: 143
Module mE04; ngenes: 88
Module mE05; ngenes: 249
Module mE06; ngenes: 254
Module mE07; ngenes: 252
Module mE08; ngenes: 261
Module mE09; ngenes: 86
Module mE10; ngenes: 116
Module mE11; ngenes: 120
Module mE12; ngenes: 282
Module mE13; ngenes: 179
Module mE14; ngenes: 63
Module mE15; ngenes: 176
Module mE16; ngenes: 104
Module mE17; ngenes: 116
Module mE18; ngenes: 58
Module mE19; ngenes: 62
Module mE20; ngenes: 89
Module mE21; ngenes: 137
Module mE22; ngenes: 222
Module mE23; ngenes: 490
Module mE24; ngenes: 589
normalised expression (scaled)

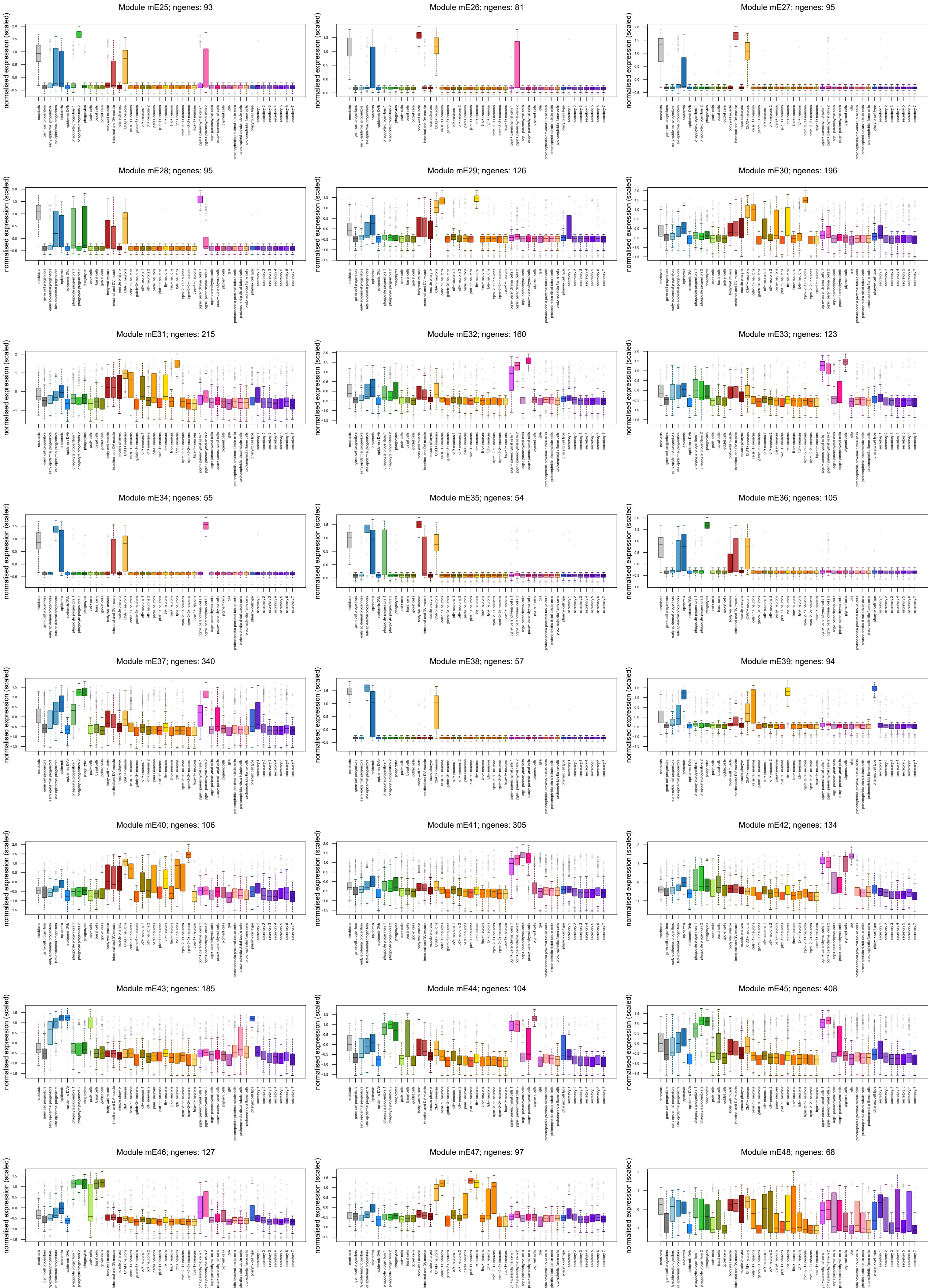
Module mE25; ngenes: 93
Module mE26; ngenes: 81
Module mE27; ngenes: 95
Module mE28; ngenes: 95
Module mE29; ngenes: 126
Module mE30; ngenes: 196
Module mE31; ngenes: 215
Module mE32; ngenes: 160
Module mE33; ngenes: 123
Module mE34; ngenes: 55
Module mE35; ngenes: 54
Module mE36; ngenes: 105
Module mE37; ngenes: 340
Module mE38; ngenes: 57
Module mE39; ngenes: 94
Module mE40; ngenes: 106
Module mE41; ngenes: 305
Module mE42; ngenes: 134
Module mE43; ngenes: 185
Module mE44; ngenes: 104
Module mE45; ngenes: 408
Module mE46; ngenes: 127
Module mE47; ngenes: 97
Module mE48; ngenes: 68
normalised expression (scaled)

Module mE49; ngenes: 216

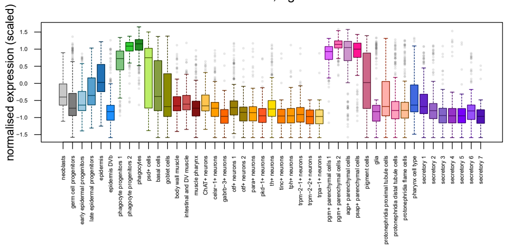

Module mE50; ngenes: 464

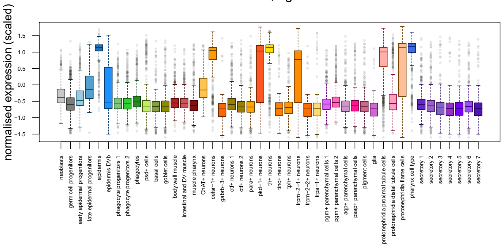

Module mE51; ngenes: 258

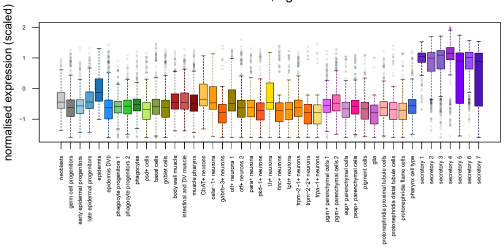

Module mE52; ngenes: 262

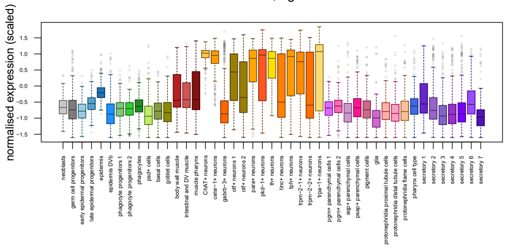

Module mE53; ngenes: 419

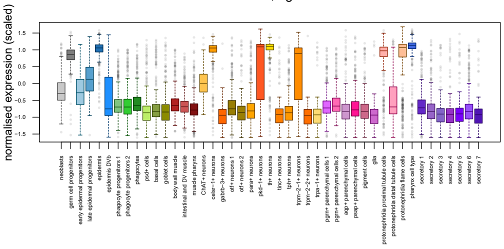

Supplement: Supplementary file 8 — Supplementary Data 5 [file 41467_2025_65712_MOESM8_ESM.pdf]
